# Antibiotic Treatment of Severe Exacerbations of Chronic Obstructive Pulmonary Disease with Procalcitonin: A Randomized Noninferiority Trial

**DOI:** 10.1371/journal.pone.0118241

**Published:** 2015-03-11

**Authors:** Alessia Verduri, Fabrizio Luppi, Roberto D’Amico, Sara Balduzzi, Roberto Vicini, Anna Liverani, Valentina Ruggieri, Mario Plebani, Maria Pia Foschino Barbaro, Antonio Spanevello, Giorgio Walter Canonica, Alberto Papi, Leonardo Michele Fabbri, Bianca Beghè, on behalf of the FARM58J2XH Study Group

**Affiliations:** 1 Department of Oncology, Haematology and Respiratory Diseases, University of Modena & Reggio Emilia, Modena, Italy; 2 Statistics Unit, Department of Diagnostic and Clinical Medicine and Public Health, University of Modena & Reggio Emilia, Modena, Italy; 3 Department of Laboratory Medicine, University of Padova, Padova, Italy; 4 Department of Medical and Surgical Sciences, University of Foggia, Foggia, Italy; 5 Department of Clinical and Experimental Medicine, Division of Pulmonary Rehabilitation, Fondazione S. Maugeri (Tradate), University of Insubria, Varese, Italy; 6 Department of Respiratory & Allergic Diseases, University of Genova, Genova, Italy; 7 Department of Respiratory Diseases, University of Ferrara, Ferrara, Italy; The National Institute for Health Innovation, NEW ZEALAND

## Abstract

**Background:**

The duration of antibiotic treatment of exacerbations of COPD (ECOPD) is controversial. Serum procalcitonin (PCT) is a biomarker of bacterial infection used to identify the cause of ECOPD.

**Methods and Findings:**

We investigated whether a PCT-guided plan would allow a shorter duration of antibiotic treatment in patients with severe ECOPD. For this multicenter, randomized, non-inferiority trial, we enrolled 184 patients hospitalized with ECOPD from 18 hospitals in Italy. Patients were assigned to receive antibiotics for 10 days (standard group) or for either 3 or 10 days (PCT group). The primary outcome was the rate of ECOPD at 6 months. Having planned to recruit 400 patients, we randomized only 183: 93 in the PCT group and 90 in the standard group. Thus, the completed study was underpowered. The ECOPD rate at 6 months between PCT-guided and standard antibiotic treatment was not significant (% difference, 4.04; 90% confidence interval [CI], −7.23 to 15.31), but the CI included the non-inferiority margin of 15. In the PCT-guided group, about 50% of patients were treated for 3 days, and there was no difference in primary or secondary outcomes compared to patients treated for 10 days.

**Conclusions:**

Although the primary and secondary clinical outcomes were no different for patients treated for 3 or 10 days in the PCT group, the conclusion that antibiotics can be safely stopped after 3 days in patients with low serum PCT cannot be substantiated statistically. Thus, the results of this study are inconclusive regarding the noninferiority of the PCT-guided plan compared to the standard antibiotic treatment. The study was funded by Agenzia Italiana del Farmaco (AIFA-FARM58J2XH). Clinical trial registered with www.clinicaltrials.gov (NCT01125098).

**Trial Registration:**

ClinicalTrials.gov NCT01125098

## Introduction

Exacerbations of chronic obstructive pulmonary disease (ECOPD) constitute 2.4% of all acute hospital admissions in England [1] and are associated with a mortality rate as high as 40% in the first year following hospitalization in patients needing mechanical support for acute ECOPD [2].

The most common causes of ECOPD are viral and/or bacterial respiratory tract infections and air pollution. However, a precise cause cannot be identified in a large number of patients [1–4].

Long-term antibiotic treatment (e.g., 12 months of 250 mg azithromycin daily) prevents ECOPD and hospitalization in patients with severe COPD [5,6], possibly by reducing the bacterial load and/or bronchial inflammation in the airways. However, it may also increase bacterial resistance, cardiovascular mortality due to prolongation of the heart rate-corrected QT (QTc) interval [7], and hearing loss. Thus, long-term antibiotic treatment is not recommended by most recent international guidelines [8].

The current approach to treatment of ECOPD due to bacterial infections is antibiotic treatment, even though the precise role of bacterial infections and antibiotic treatment in individual episodes of severe ECOPD remains controversial [9,10]. Identification of the bacterial etiology of ECOPD is often unclear, and the decision to treat with antibiotics is usually empirical; antibiotics are, of course, useful only for treating bacterial infections. Taking into account the limited evidence available, i.e., that from only one properly designed randomized clinical trial [11], current international guidelines recommend antibiotics only in ECOPD with (i) three cardinal symptoms, i.e., increased dyspnea, sputum volume, and sputum purulence (type 1) [11] or (ii) two cardinal symptoms, with one of the two being sputum purulence (type 2), and/or (iii) respiratory failure [11] with an arbitrary duration of 5–10 days [8,12].

Previous studies investigated the role of biomarkers of bacterial infection [13–15], particularly procalcitonin (PCT), in guiding antibiotic treatment in respiratory infections, i.e., pneumonia and ECOPD [16–20]. PCT is a prototype of a “hormokine” mediator that is released in bacterial infections but not in viral infections or noninfectious stimuli [21]. Indeed, PCT-guided antibiotic treatment may reduce the use of antibiotics in patients hospitalized for ECOPD [17,19,20]. However, none of those studies consisted only of patients with Anthonisen’s type 1 ECOPD [11] or respiratory failure, for whom guidelines recommend antibiotic treatment [8,12], and none investigated the role of PCT in reducing the duration of antibiotic treatment.

Antibiotic treatment for up to 10 days is supported by a single clinical trial published 28 years ago_[11]; in fact, both the previous (3–10 days) [22] and current (5–10 days) recommendations [8,12] have been given only a D level of evidence, i.e., panel consensus judgment. Also, although GOLD guidelines only mention PCT as a biomarker of bacterial infection_ [8], a recent *JAMA* clinical evidence synopsis [23] does not recommend its use.

For all these reasons, we decided to investigate whether antibiotic treatment could be safely stopped according to a PCT-guided 3-day treatment plan in patients hospitalized with ECOPD.

## Methods

### Study Design and Participants

The protocol for this trial and supporting CONSORT checklist are available as supporting information; see S7 CONSORT Checklist and S8 Protocol.

In a randomized, multicenter, open, controlled, parallel-group, noninferiority trial involving 18 university/city hospital pulmonary departments, we compared patients hospitalized with severe ECOPD who were receiving the standard 10-day course of antibiotic therapy recommended by the 2005 GOLD guidelines [22] with those receiving a 3- or 10-day antibiotic course guided by a PCT plan. According to current recommendations [22, 24], because all patients recruited for the study required hospitalization, the exacerbations were considered severe. Apart from the usual clinical investigations, which included routine blood tests, arterial blood gases, ECG, and chest X-ray, no other specific clinical investigation was used to assess the severity of exacerbations. Patients were recruited and followed up between 26 January 2007 and 25 July 2011.

Study participants were male or female, ≥18 years of age, current or former smokers, and diagnosed with COPD stages I-IV as defined by GOLD guidelines available at the time the study was designed, with protocol deviation [22]. Patients were hospitalized for severe ECOPD requiring antibiotic treatment, i.e., type 1 exacerbation (increased dyspnea, sputum volume, and sputum purulence verified by the attending clinician) according to Anthonisen [11], and/or characterized by respiratory failure. ECOPD was defined as “an acute event characterized by a worsening of the patient’s respiratory symptoms that is beyond normal day-to-day variations and leads to a change in medication” [22,24].

Exclusion criteria included bronchial asthma, unstable concomitant disease (cardiovascular, renal, hepatic, gastrointestinal, neurological, metabolic, musculoskeletal, neoplastic, respiratory or other disease), pregnancy and breastfeeding, clinically significant laboratory abnormalities suggestive of unstable concomitant disease, survival for 1 year unlikely, and inability to give written consent. Antibiotic pretreatment before hospital admission and radiographic signs of pneumonia did not preclude eligibility for the study. All patients underwent chest X-ray at admission. Only one patient recruited in the standard group had community-acquired pneumonia, and remained in the study group.

Patients’ clinical data, comorbidities, and routine blood test results were recorded at the time of recruitment. According to the protocol, all patients had to have a record of lung function testing confirming COPD (see below) or spirometric tests in the hospital according to guidelines [25]. All patients included in the analysis had hospital spirometry. Patients were included in the study if they had a FEV_1_/FVC <0.7 and FEV_1_ <80% predicted [8,22]. In addition, all patients underwent arterial blood gas analysis at admission, on Day 1, and on Day 2. Respiratory failure was defined as PaO_2_ <60 mm Hg with or without PaCO_2_ >50 mm Hg when breathing room air [8,22].

The study (which conformed to the Declaration of Helsinki) was approved by the ethics committees of each of the 18 participating centers. Names of members of the ethics committee/institutional review board(s) that approved the study are reported in Supporting Information. All patients gave informed written consent. The trial was approved and funded by the Agenzia Italiana del Farmaco (AIFA), the Italian agency for drugs that is an official body of the Italian Ministry of Health. The title of the protocol, approval and funding were registered in the AIFA registry (http://www.agenziafarmaco.gov.it/, <http://www.agenziaitalianadelfarmaco.gov.it,farm58j2xh/> FARM58J2XH) and the protocol was posted by AIFA in the European clinical trials register (https://www.clinicaltrialsregister.eu/ctr-search/search, 2006-005354-68) on 2007/07/04. The trial was registered by us at http://www.clinicaltrial.gov/ (NCT01125098) in 2010. The reason for the late registration at http://www.clinicaltrial.gov was that we thought that registration in the https://www.clinicaltrialsregister.eu/ctr-search/search represented sufficient evidence of trial publication. We confirm that all ongoing and related trials for this drug/intervention are registered.

### Randomization and Treatment

**Fig 1:**
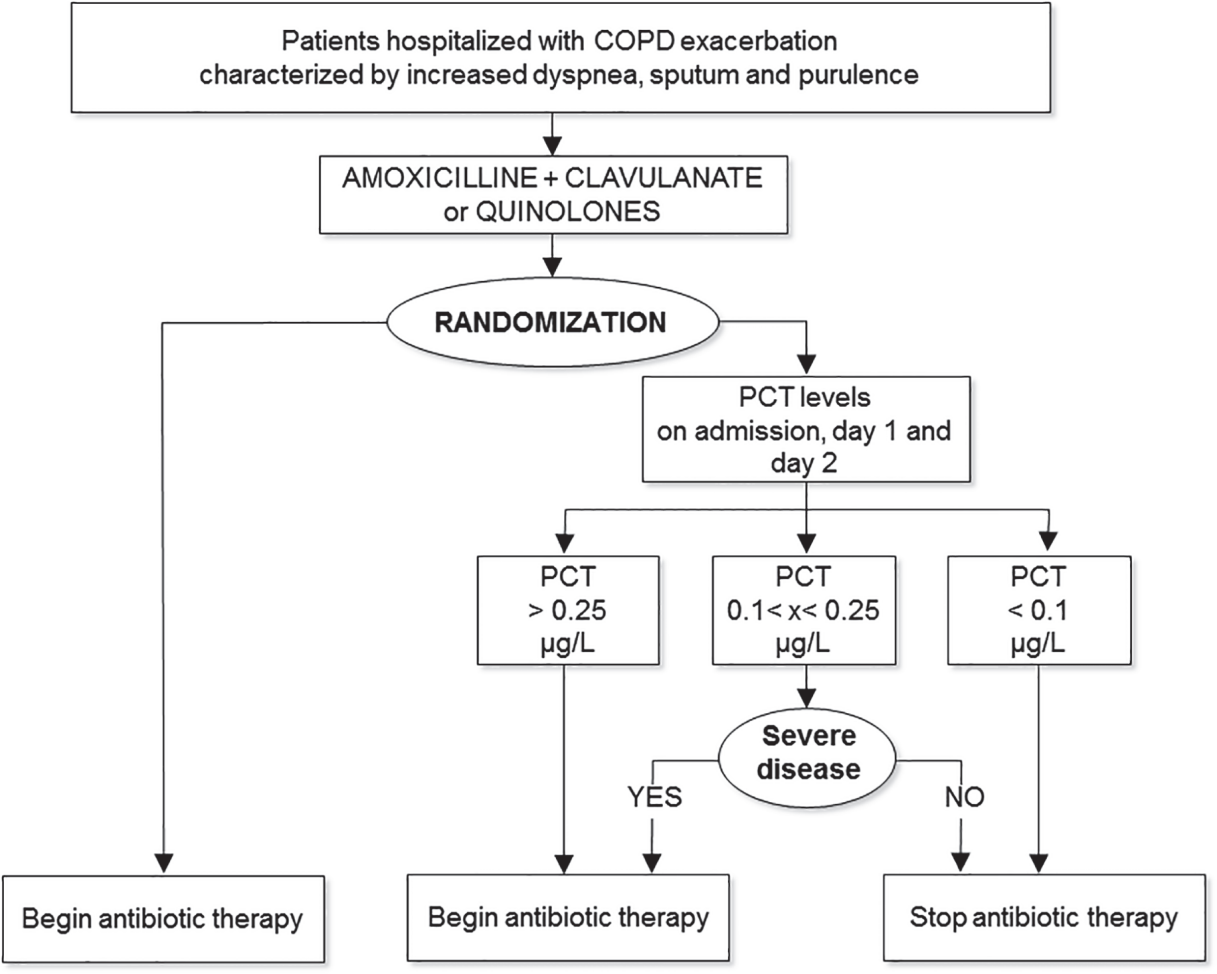
Trial protocol. Severe disease = respiratory failure or clinical instability.

The independent Clinical Trials and Methodological Unit at the University of Modena carried out centralized randomization. Eligible patients were randomly assigned to receive standard antibiotic therapy (standard group) or PCT-guided antibiotic treatment (PCT group) according to a 1:1 permuted block computer-generated scheme, stratified according to hospital. The randomization was Web-based, and only statisticians and the website administrator knew the randomization sequence. On admission, all patients received a 3-day course of antibiotics (either amoxicillin plus clavulanate or quinolones) according to 2005 international guidelines [22]. PCT levels were measured on hospital admission, on Day 1, and on Day 2. On Day 2 each eligible patient was randomly assigned to one of the two treatment plans. Patients randomized to the standard group continued antibiotic therapy for 10 days, whereas patients randomized to the PCT group either continued treatment for 10 days or stopped on Day 3, depending on their PCT levels, according to previously recommended cut-off values [17,20]. Specifically, patients continued antibiotic treatment for 10 days if one or more of the PCT values on the first 3 days of hospitalization were ≥0.25 μg/L. When PCT values were <0.25 μg/L but ≥0.1 μg/L on any occasion, antibiotic treatment was continued for 10 days, but only if patients 0were clinically unstable or had acute respiratory failure; otherwise, treatment was stopped on Day 3. If all PCT values were consistently <0.1 μg/L, treatment was stopped on Day 3 (Fig. 1). Because the final decision about maximum duration of treatment in randomized patients was left to the referring investigator, who had the option of overruling the PCT-guided plan if he or she deemed it clinically inappropriate, 4 patients with low values of PCT received treatment between 4 and 10 days. All patients were also treated with (i) systemic corticosteroids for 14 days [22], plus (ii) regular inhaled short-acting or long-acting bronchodilators.

### Outcomes and Follow-up

We prospectively followed patients during hospitalization and after discharge. Patients were clinically assessed on admission, on Days 1 and 2 after hospitalization, and on Day 10 or at discharge. Blood samples were obtained for measurement of PCT and serologic testing (*Mycoplasma pneumoniae*, *Chlamydia pneumoniae*, and *Legionella pneumophila* [Virotech ELISA IgG and IgM; Vircell ELISA IgG and IgM; Virion Serion ELISA classic IgG and IgM]). Sputum was collected for Gram staining and culture. Microbiological analysis was carried out according to Isenberg [26], and all patients had a chest X-ray. Only one patient in the standard group had radiological evidence of pneumonia, and he was negative for serological detection of bacterial infection. In addition, each patient filled out quality-of-life questionnaires (Short Form 36, baseline [BDI] and transition [*TDI*] *dyspnea indices*, and CCIQ [Chronic Cough Impact Questionnaire]) on admission [27–30]. Follow-up visits were scheduled on Day 1, Day 3, and 6 months after discharge; telephone interviews were conducted at 2, 4, and 5 months after discharge.

The primary end point of the study was the percentage of patients with at least one exacerbation within 6 months after the index exacerbation. Secondary end points included hospital readmission, admission to the intensive care unit, change in lung function (ΔFEV_1_), length of hospital stay, and death from any cause.

The outcome of the index exacerbation was considered a clinical success when the signs and symptoms associated with the exacerbation were completely resolved or improved. Treatment failure was defined as a lack of resolution, lack of attenuation of signs and symptoms, worsening of signs and symptoms, or death.

The patient’s safety was monitored at each visit, including the assessment of adverse events related to the antibiotic treatment and of clinical events such as pneumonia, pulmonary embolism, acute pulmonary edema, and any cardiovascular accident.

### Measurement of Procalcitonin

Quantitative measurements of PCT concentrations were performed in a centralized laboratory (Department of Laboratory Medicine, University of Padova, Italy), in duplicate in human serum samples (volume: 50 μL), using an automated immunofluorescent assay (Kryptor PCT; Brahms Diagnostica AG, Hennigsdorf/Berlin, Germany). The principle of the method is based on TRACE technology (time-resolved amplification of cryptate emission), which measures the signal emitted from an immunocomplex with time delay. The basis of TRACE technology is nonradiating energy transfer from a donor molecule (cryptate) to a fluorescent label (XL665) acceptor molecule; the long-life signal emitted is proportional to the concentration of the analyte to be measured (PCT) [31]. The characteristics of the test have been previously described [17,20]. Samples were couriered from each participating center to the Central Laboratory of Padova with the commitment to deliver the results of the 3-day samples within 24 hours of the previous sample.

### Statistical Analysis

Sample size was determined according to the primary outcome of the study (cumulative ECOPD rate at 6 months), calculated by dividing the number of patients with at least one exacerbation during the 6-month follow-up by the total number of patients initially randomized. Data from previous studies [20], used to estimate the frequency of the primary outcome of the present study, suggested that exacerbations within 6 months occur in approximately 50% of patients. To define the noninferiority of the PCT-guided algorithm compared to the standard guidelines-recommended plan, we settled on a 15% difference in the percentage of patients with an exacerbation within the 6-month follow-up as a clinically tolerable upper limit. We hypothesized that exacerbation rates for both treatment plans would be equal (at 50%), and we considered a difference of ≤15% to be irrelevant; then, setting β = 0.20 and α = 0.05 (one-tail), the estimated sample size was 140 patients per arm. Taking into account an estimated 30% drop-out rate of patients at the 6-month follow-up, we increased the sample size to 200 patients per arm.

Data were summarized as mean ± standard deviation (SD), or as median and interquartile range (IQR) when appropriate, for continuous variables or as number and percentage for categorical variables.

To compare PCT-guided and standard groups in terms of primary outcome, we estimated the risk difference and its relative 90% confidence interval (CI). For secondary outcomes, risks and mean differences as well as their 95% CIs were calculated for comparing binary and continuous outcomes, respectively. Hazard ratios and their 95% CIs were estimated to compare time-to-event data. Categorical data were compared with the use of the chi-square test or the Fisher exact test, when appropriate. Continuous data were compared with the use of Student’s t test. Cox proportional hazards regression analysis was used to estimate hazard ratios and 95% CIs for time-to-event data (e.g., length of hospital stay).

Statistical analyses were performed by using STATA version 12 (StataCorp LP, College Station, TX).

## Results

### Study Population

**Fig 2:**
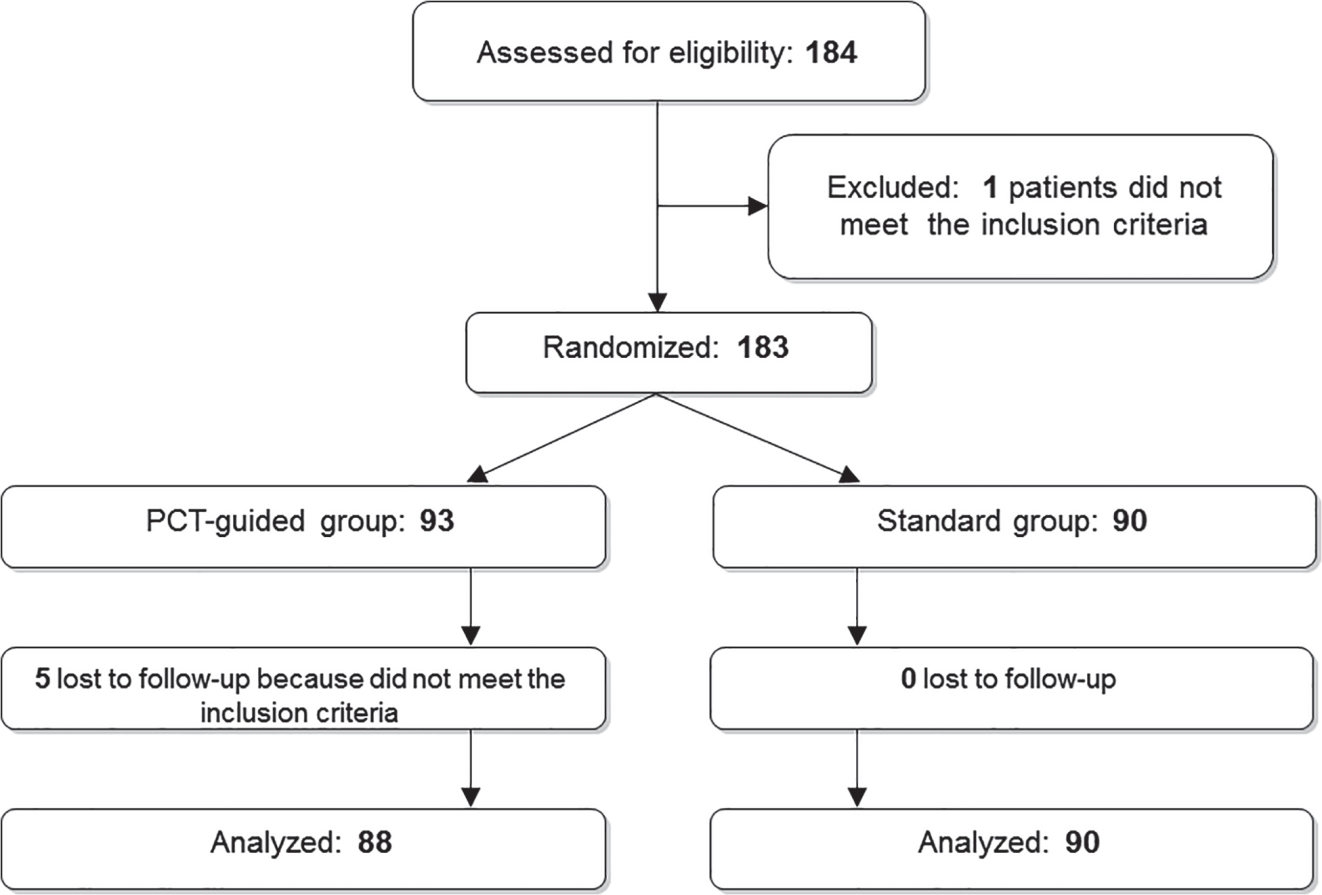
Trial profile: screening, enrollment, randomization, and follow-up.

We planned to recruit 400 patients. However, because of slow recruitment by the centers and possibly because of the very strict inclusion criteria (Anthonisen type 1 and/or respiratory failure), we were able to screen only 184 patients and randomize 183: 93 into the PCT group, and 90 into the standard group (Fig. 2). Five patients in the PCT group were not included in the analyses because they were randomized by mistake; they actually did not meet the inclusion criteria. No patient in the standard group was lost to follow-up. According to the protocol, within the PCT group, 45 patients stopped antibiotics after a 3-day course, and 43 patients continued antibiotics for 10 days. Clinical characteristics of the study participants were similar in all groups (Table 1). Overall, 18.5% of all patients received antibiotics before admission, with no difference between groups (p = 0.481), and the duration of antibiotic treatment was similar even in the subgroups (p = 0.942). Use of other medications prescribed before admission was no different between groups (Table 1). FEV_1_ (% of predicted value) was similar in the two groups (41.3% in the PCT group and 42.7% in the standard group; p = 0.597). Overall, 80% of patients (78% in the standard group and 83% in the PCT group; p = 0.385) had relevant comorbidities.

**Table 1:** Characteristics of Patients Randomized to PCT (Procalcitonin) Group (3 or 10 Days of Antibiotics) or Standard Group (10 Days of Antibiotics) at Recruitment.

Characteristic	PCT			Standard
	3 days	10 days	All PCT patients	10 days
	(n = 45)	(n = 43)	(N = 88)	(N = 90)
Sex				
Female	5/45 (11.1)	6/43 (14.0)	11/88	13/90 (14.4)
Age, median (IQR)	72 (69–78)	74 (67–78)	73.5 (68.5–78)	72.5 (65–78)
Smoking history, pack-yr	48.5 (27.5–66)	45 (30–60)	45 (30–60)	45 (30–68)
Current smokers	13/45 (28.9)	10/43 (23.3)	23/88 (26.14)	22/88 (25.0)
Duration of ECOPD, days	5 (2–7)	3 (0–6)	3.5 (2–7)	5 (2–8)
Symptoms
Cough	42/44 (95.5)	40/42 (95.2)	82/86 (95.4)	82/87 (94.3)
Increased sputum production	36/43 (83.7)	36/41 (87.8)	72/84 (85.7)	72/86 (83.7)
Sputum purulence	29/43 (67.4)	35/42 (83.3)	64/85 (75.3)	64/84 (76.2)
Dyspnea	41/44 (93.2)	41/42 (97.6)	82/86 (95.4)	87/87 (100)
Fever	11/43 (25.6)	15/42 (35.7)	26/85 (30.6)	25/86 (29.1)
Maintenance therapy at hospital admission
Inhaled β_2_-bronchodilators	8/45 (17.8)	8/43 (18.6)	16/88 (18.2)	16/90 (17.8)
Inhaled anticholinergics	22/45 (48.9)	25/43 (58.1)	47/88 (53.4)	48/90 (53.3)
Inhaled corticosteroids	4/45 (8.9)	6/43 (14.0)	10/88 (11.4)	18/90 (20.0)
Oral corticosteroids	0/45 (0)	0/43 (0)	0/88 (0)	2/90 (2.2)
Combination inhaled β_2_-bronchodilators + corticosteroids	18/45 (40.0)	23/43 (53.5)	41/88 (46.6)	35/90 (38.9)
Combination inhaled β_2_-bronchodilators + anticholinergics	3/45 (6.7)	2/43 (4.7)	5/88 (5.7)	6/90 (6.7)
Theophylline	9/45 (20.0)	12/43 (27.9)	21/88 (23.9)	22/90 (24.4)
Long-term oxygen therapy	1/45 (2.2)	3/43 (7.0)	4/88 (4.6)	3/90 (3.3)
Previous therapy with antibiotics at hospital admission	7/45 (15.6)	9/43 (20.9)	16/88 (18.2)	17/90 (18.9)
Length of hospital stay, days	6.2 ± 5.4	5.2 ± 3.0	5.7 ± 4.2	5.8 ± 3.7
Home treatment for ECOPD
Oral or parenteral corticosteroids	7/45 (15.56)	6/43 (13.95)	13/88 (14.77)	16/90 (17.78)
Inhaled steroids	0/45 (0)	2/43 (4.65)	2/88 (2.27)	5/90 (5.56)
β_2_-agonist and/or anticholinergic agents	0/45 (0)	2/43 (4.65)	2/88 (2.27)	9/90 (10.0)
Theophylline	1/45 (2.22)	2/43 (4.65)	3/88 (3.41)	2/90 (2.22)
Comorbidities
Arterial hypertension	24/45 (53.3)	22/43 (51.2)	46/88 (52.3)	51/90 (56.7)
Chronic heart failure	5/45 (11.1)	5/43 (11.6)	10/88 (11.4)	7/90 (7.8)
Peripheral artery disease	3/45 (6.7)	4/43 (9.3)	7/88 (8.0)	4/90 (4.4)
Other cardiovascular disease	11/45 (24.4)	13/43 (30.2)	24/88 (27.3)	20/90 (22.2)
Hypercholesterolemia	1/45 (2.2)	5/43 (11.6)	6/88 (6.8)	5/90 (5.6)
Diabetes	13/45 (28.9)	13/43 (30.2)	26/88 (29.6)	19/90 (21.1)
Cerebrovascular disease	2/45 (4.4)	2/43 (4.7)	4/88 (4.6)	2/90 (2.2)
Osteoporosis	0/45 (0)	1/43 (2.3)	1/88 (1.1)	3/90 (3.3)
Cancer	0/45 (0)	3/43 (7.0)	3/88 (3.4)	4/90 (4.4)
Chronic renal failure	0/45 (0)	3/43 (7.0)	3/88 (3.4)	6/90 (6.7)
Anxiety/depression	2/45 (4.4)	5/43 (11.6)	7/88 (8.0)	6/90 (6.7)
Severity of COPD
GOLD stage I	1/43 (2.3)	1/42 (2.4)	2/85 (2.4)	1/82 (1.2)
GOLD stage II	12/43 (27.9)	9/42 (21.4)	21/85 (24.7)	23/82 (28.1)
GOLD stage III	22/43 (51.2)	18/42 (42.9)	40/85 (47.1)	31/82 (37.8)
GOLD stage IV	8/43 (18.6)	14/42 (33.3)	22/85 (25.9)	27/82 (32.9)
Heart rate, beats/min	81 ± 12	83 ± 15	82 ± 13	84 ± 14
BP, mm Hg
Systolic	127 ± 14	133 ± 20	130 ± 17	133 ± 1
Diastolic	76 ± 9	76 ± 11	76 ± 10	78 ± 10
SpO_2_	93 (91–95)	91.5 (87–94)	92.5 (90–95)	93 (90–95)
pH	7.41 (7.40–7.43)	7.40 (7.36–7.42)	7.41 (7.39–7.43)	7.41 (7.38–7.44)
PaO_2_	66.0 (60.0–74.5)	60.5 (52.0–67.0)	63.0 (56.0–71.0)	66.0 (58.0–73.0)
PaCO_2_	42.0 (38.5–49.5)	44.0 (41.0–51.0)	43.0 (39.0–51.0)	42.0 (38.0–50.0)

Values are given as no. (%), mean ± SD, or median (IQR).

The rate of positive cultures from sputum was similar in the standard group (16.7%) and the PCT group (17%) (p = 0.946). Gram-negative bacteria accounted for 73% of all microorganisms recovered, and gram-positive bacteria were found in 27% of samples. In both groups, the predominant bacteria detected were *Pseudomonas* spp. (4%), *Haemophilus influenzae* (3.37%), *Streptococcus pneumoniae* (1.68%), and *Staphylococcus aureus* (1.68%). Bacterial infections were serologically diagnosed in 30 patients (*C*. *pneumoniae*, n = 13; *M*. *pneumoniae*, n = 13; *L*. *pneumophila*, n = 4), even though only one patient in the standard group had radiological evidence of pneumonia; he was negative for the serological detection of bacterial infection.

On admission, the median PCT level (IQR) was 0.12 μg/L (25th-75th centile, 0.08–0.18) in the standard group, 0.21 μg/L (0.13–0.57) in the PCT subgroup who continued antibiotics for 10 days, and 0.08 μg/L (0.06–0.12) in the PCT subgroup who stopped antibiotics after 3 days. As expected, PCT levels decreased on Day 1 and Day 2 (Table 2).

**Table 2:** Blood Levels of PCT at Days 0, 1, and 2 by Randomization Arm.

Characteristic	PCT			Standard
		3 days	10 days	All PCT patients	10 days
	(n = 45)	(n = 43)	(N = 88)*	(N = 90)
PRT levels				
Day 0 (at admission)				
n	43	43	86	86
Mean	0.09	0.46	0.27	0.55
Median	0.08	0.15	0.1	0.11
SD	0.04	1.43	1.02	3.67
Min-max	0.02–0.2	0.04–9.4	0.02–9.4	0.02–34.1
No. (%) patients with PCT values <0.1 μg/L	29 (64.44)	10 (23.26)	39 (44.32)	41 (45.56)
No. (%) patients with PCT values 0.1–0.25 μg/L	14 (31.11)	21 (48.84)	35 (39.77)	34 (37.78)
No. (%) patients with PCT values >0.25 μg/L	0 (0.00)	12 (27.91)	12 (13.64)	11 (12.22)
Missing (%)	2 (4.44)	0 (0.00)	2 (2.27)	4 (4.44)
Day 1				
n	43	41	84	84
Mean	0.07	0.34	0.20	0.46
Median	0.07	0.13	0.09	0.09
SD	0.04	0.64	0.47	2.84
Min-max	0.01–0.18	0.05–4.00	0.01–4.00	0.03–26.1
No.(%) patients with PCT values <0.1 μg/L	35 (77.78)	9 (20.93)	44 (50.00)	45 (50.00)
No.(%) patients with PCT values 0.1–0.25 μg/L	8 (17.78)	20 (46.51)	28 (31.82)	30 (33.33)
No.(%) patients with PCT values >0.25 μg/L	0 (0.00)	12 (27.91)	12 (13.64)	9 (10.00)
Missing (%)	2 (4.44)	2 (4.65)	4 (4.55)	6 (6.67)
Day 2				
n	43	40	83	88
Mean	0.08	0.28	0.17	0.27
Median	0.07	0.15	0.1	0.08
SD	0.04	0.34	0.26	1.47
Min-max	0.02–0.19	0.05–1.9	0.02–1.9	0.03–13.8
No.(%) patients with PCT values <0.1 μg/L	32 (71.11)	9 (20.93)	41 (46.59)	49 (54.44)
No.(%) patients with PCT values 0.1–0.25 μg/L	11 (24.44)	19 (44.19)	30 (34.09)	33 (36.67)
No.(%) patients with PCT values > 0.25 μg/L	0 (0.00)	12 (27.91)	12 (13.64)	6 (6.67)
Missing (%)	2 (4.44)	3 (6.98)	5 (5.68)	2 (2.22)

*Originally 91 patients in this group; three were lost to follow-up.

Sputum purulence was observed in 72% of patients (n = 128). Interestingly, PCT levels were no different between patients with sputum purulence and patients with respiratory failure without sputum purulence (8.4%, n = 15) (p = 0.894).

### Primary and Secondary Outcomes

Fifty-three patients experienced at least one exacerbation in the 6 months after hospital discharge. The number of patients with at least one exacerbation within 6 months after hospital discharge was no different between the PCT (n = 28) and standard (n = 25) groups (Table 3) (risk difference [90% CI], 4.04 (−7.23;15.31), even when the upper limit of the 90% CI (15.31) went beyond the predefined noninferiority margin of 15. The number of exacerbations was also no different between groups (Table 4).

**Table 3:** Clinical Outcome Parameters at 6-Month Follow-up in Patients with ECOPD According to Treatment Plan.

	Procalcitonin			Standard	Measure of association(CI)*
Outcome	3 days	10 days	All PCT patients	10 days	
	(n = 45)	(n = 43)	(N = 88)	(N = 90)		
Primary outcome, no. (%) of patients with at least 1 exacerbation					RD (90% CI)
	12 (26.67)	16 (37.21)	28 (31.82)	25 (27.78)	4.04 (−7.23;15.31)
Secondary outcomes					
Binary outcomes, no. (%)					RD (95% CI)
Subsequent antibiotic use for the treatment of ECOPD within 6 months	10 (22.22)	8 (18.60)	18 (20.45)	12 (13.33)	7.12 (−3.85;18.09)
Clinical success	27 (60.00)	31 (72.09)	58 (65.91)	51 (56.67)	9.24 (−5.00;23.49)
Hospital readmission for ECOPD within 6 months	4 (8.89)	9 (20.93)	13 (14.77)	8 (8.89)	5.88 (−3.58;15.35)
Hospital readmission for any cause	5 (11.11)	10 (23.26)	15 (17.05)	10 (11.11)	5.93 (−4.26;16.17)
Need for ICU stay	0 (0)	1 (2.32)	1 (1.14)	0 (0)	1.14 (−1.08;3.35)
Respiratory failure	10 (22.22)	20 (46.51)	30 (34.09)	27 (30.00)	4.09 (−9.61;17.79)
Death from any cause within 6 mo.	1 (2.22)	2 (4.65)	3 (3.41)	2 (2.22)	1.19 (−3.67;6.05)
Continuous outcomes, mean ± SD					MD (95% CI)
Change in lung function (ΔFEV_1_, liters)	0.04 ± 0.31	0.13 ± 0.29	0.08 ± 0.30	0.11 ± 0.24	−0.03 (−0.11;0.05)
Change in lung function (ΔFEV_1_, % pred.)	2.97 ± 13.12	5.5 ± 11.11	4.11 ± 12.20	4.10 ± 13.11	0.01 (−3.74;3.76)
Time-to-event outcome, median (IQR)					HR (95% CI)
Length of hospital stay, days	5 (2–6)	5.5 (4–7)	5 (4–7)	5 (4–7)	1.08 (0.74;1.60)

* Association measures and CIs were calculated by comparing the standard group and the PCT (“All PCT patients”) groups.

IQR = interquartile range; RD = risk difference; MD = mean difference; HR = hazard ratio; CI = confidence interval.

**Table 4:** Number of Exacerbations During the 6-Month Follow-up.

	PCT 3 days n = 45	PCT 10 days n = 43	All PCT N = 88	Standard N = 90	p-value
No (%) of patients with 1 exacerbation	8 (17.78)	12 (27.91)	20 (22.73)	17 (18.89)	0.798
No (%) of patients with 2 exacerbations	4 (8.89)	3 (6.98)	7 (7.95)	7 (7.78)	
No (%) of patients with 3 exacerbations	0 (0)	1 (2.33)	1 (1.14)	1 (1.11)	
Total no. of exacerbations	16	21	37	34	

**Fig 3:**
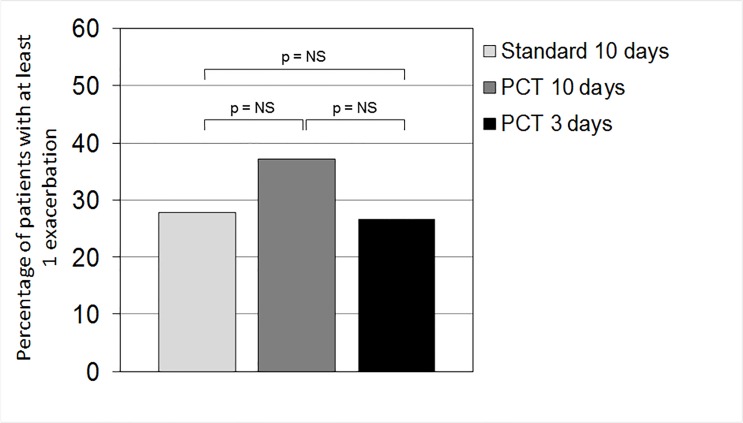
Percentage of patients with at least one exacerbation during the 6 months of follow-up in the three groups: patients who received antibiotics for 3 days (PCT 3 days) or 10 days (PCT 10 days) according to a PCT-guided plan, or for 10 days according to the standard plan. P = not significant.

Antibiotic treatment during the 6 months of follow-up was no different between groups (PCT, 20.45% vs. standard, 13.33%; risk difference [95% CI], 7.12 (−3.85;18.09]) (Table 3 and Fig. 3). Time to the next exacerbation in patients treated with antibiotics in the PCT and standard groups was also no different (hazard ratio [95% CI], 1.31[0.69;2.48]). All other clinical measures of outcome and hospitalization were similar between the two groups at the 6-month follow-up (Table 2). Similarly, the scores on quality-of-life questionnaires were no different between groups. The incidence of subsequent pneumonia was also no different between groups (p = 0.365). Few adverse events were reported: 3 deaths in the PCT group and 2 deaths in the standard group (Table 5).

**Table 5:** Adverse Events among Study Participants.

Characteristic	PCT			Standard
		3 days	10 days	All PCT patients	10 days
	(n = 45)	(n = 43)	(N = 88)	(N = 90)
No. of patients with at least one serious adverse event (SAE)	1	2	3	3
No. of patients with at least one non-SAE	-	1	1	2
Reported SAE				
Death	1	2	3	2
Cause of death*	SCLC	MI,SD		AHF,ARF
Pneumonia	-	-	-	1
Reported non-SAE				
Cough	-	-	-	1
High blood pressure level	-	-	-	1
Gastric pain	-	1**	1**	-
Fever	-	-	-	1
Vomitus	-	1**	1**	-
Dyspnea	-	-	-	1
Facial paralysis	-	-	-	1

*SCLC = small cell lung cancer; MI = myocardial infarction; SD = sudden death; AHF = acute heart failure; ARF = acute respiratory failure.

**Probably related to the study medication.

## Discussion

Antibiotic treatment of ECOPD patients is widespread, even though evidence for both the bacterial etiology and the efficacy of antibiotic treatment in ECOPD is still controversial. The recognition of clinical/biochemical characteristics that would (i) identify patients with ECOPD due to bacterial infection, (ii) select patients who could most benefit from antibiotic treatment, and (iii) determine the appropriate duration of antibiotic treatment would improve ECOPD management and reduce antibiotic use.

The results of our study failed to demonstrate the noninferiority of PCT-guided antibiotic treatment compared to standard treatment, even though the difference in the upper limit of the 90% CI (15.31) went just slightly beyond the predefined non-inferiority margin (15.31 versus 15). However, we found no significant difference in primary or secondary outcomes between the PCT and standard groups and, more important, between the patients who received antibiotics for 3 days and those who received antibiotics for 10 days. Thus, the results of this study suggest that, in a subject with low levels of PCT, the duration of antibiotic treatment might safely be reduced to 3 days.

Even if a properly designed and powered study is required for corroboration before a specific recommendation can be made, our results are consistent with the efficacy and safety of the PCT-guided antibiotic treatment plan recently suggested in two important articles, i.e., a Cochrane review [32] and an authoritative synopsis of clinical evidence [23].

Our study has several limitations. First, it was underpowered, because we planned to enroll 400 patients and eventually randomized only 183. Thus, the conclusion that antibiotics can be safely stopped after 3 days in patients with low serum PCT cannot be substantiated statistically. Second, we arbitrarily decided on a 10-day duration for the standard group even though the 2005 guidelines recommended 3–10 days [22]. This decision was made because of the severity of ECOPD, which in our study required hospitalization. Third, the calculation of the sample was based on a 50% re-exacerbation risk at 6 months according to a previous similar study [20], whereas the incidence we observed in the standard group was 27.78%. We can only speculate on this discrepancy, but we believe that the lower incidence at 6 months might have been due to two major differences in our study compared to the previous study: (1) We included only patients with either all three criteria of Anthonisen (increased dyspnea, sputum volume, and sputum purulence) or respiratory failure; and (2) we treated all patients for 10 days with full-dose inhaled bronchodilator/combination therapy and a course of oral steroids. Fourth, because we anticipated that the primary outcome (exacerbations of COPD) would be strong and easy to identify, and thus unlikely to be biased by investigator influence, we did not adopt any procedure to reduce bias during the follow-up part of the trial.

To the best of our knowledge, this study is the first randomized multicenter clinical trial testing a PCT-guided antibiotic treatment plan to include only hospitalized patients with severe ECOPD with clinical characteristics requiring antibiotic treatment according to guidelines [8,22]. Previous studies have tested the PCT-guided antibiotic treatment plan in patients hospitalized because of lower respiratory infections and/or ECOPD, but none has specifically included only patients with type 1 Anthonisen’s ECOPD and/or respiratory failure requiring antibiotic treatment [16,17,19,20]. In addition, all previous studies were designed and powered to assess the effect of a PCT plan on the prescription/exposure of patients to antibiotics, and were not designed and powered on clinical outcomes. Also, none of the previous studies investigated the role of a PCT-guided plan for deciding the duration of treatment with antibiotics. Therefore, previous studies could not provide evidence to support or modify the current guidelines regarding duration of antibiotic treatment [8].

In contrast with previous studies, our study was conducted only in patients with clinical characteristics requiring antibiotics, and thus could provide novel evidence about the role of a PCT-guided plan to decide the duration of antibiotic treatment, even though—because of lack of statistical significance regarding noninferiority—our study cannot be considered conclusive. Another strength of our study that was inconsistently present in previous studies is that, according to guidelines, all patients were given oral or intravenous corticosteroids and inhaled bronchodilators for at least 3 days. This allowed us to properly investigate the effects of antibiotics in addition to bronchodilator and steroid treatment. Surprisingly, considering the entry criteria, the prevalence of positive cultures from sputum was low (17%) compared to previously reported data (32%) [33]. We do not have a clear explanation for this discrepancy.

In conclusion, because of the low statistical power of our study, the results leave us with uncertainty about the role of PCT-guided antibiotic treatment in deciding the duration of antibiotic treatment for patients hospitalized with severe ECOPD. PCT may help to identify patients in whom a shorter duration of antibiotic treatment could be safely prescribed, but adequately powered studies are needed to affirm this possibility.

## Supporting Information

S1 CONSORT Checklist (DOC)

S1 Consent Sample informed consent form. (PDF)

S1 Protocol (PDF)

S1 Report Sample case report form. (PDF)

S1 Supporting Information Ethics committees approvals. (PDF)

S1 Table All data for primary and secondary outcomes and adverse events for all patients at all visits. (DOCX)

S2 Table List of all ethics committees’ approvals. (DOCX)

S3 Table Patient distribution by center. (PDF)
