# Supplementary material for: Antibiotic Treatment of Severe Exacerbations of Chronic Obstructive Pulmonary Disease with Procalcitonin: A Randomized Noninferiority Trial
Source: PLoS One. 2015 Mar 11;10(3):e0118241. doi: 10.1371/journal.pone.0118241 (PMC4356612; doi:10.1371/journal.pone.0118241)
Supplement: S1 Consent — (PDF) [file pone.0118241.s002.pdf]

S6 Consent

Università degli Studi di Modena e Reggio Emilia
DIPARTIMENTO INTEGRATO DI ONCOLOGIA, EMATOLOGIA E PNEUMOLOGIA
(Direttore: Prof. Pier Franco Conte)

UNIVERSITÀ DEGLI STUDI DI MODENA E REGGIO EMILIA

SERVIZIO SANITARIO REGIONALE
EMILIA-ROMAGNA
Azienda Ospedaliero - Universitaria di Modena

CLINICA DI MALATTIE DELL'APPARATO RESPIRATORIO
(Direttore: Prof. Leonardo M. Fabbri)

Modulo per il consenso informato

"CONFRONTO FRA UN REGIME TERAPEUTICO BASATO SUI LIVELLI SIERICI DI PROCALCITONINA RISPETTO AD UN REGIME TERAPEUTICO STANDARD IN PAZIENTI OSPEDALIZZATI PER RIACUTIZZAZIONE DI BRONCOPNEUMOPATIA CRONICA OSTRUTTIVA"

Io sottoscritto.. dichiaro di aver ricevuto dal dottor.................................. in data .... spiegazioni esaurienti in merito alla richiesta di partecipazione allo studio sperimentale in oggetto, secondo quanto riportato nel foglio informativo qui allegato, copia del quale mi è stata consegnata in data ....

Dichiaro di aver potuto discutere tali spiegazioni, di aver potuto porre tutte le domande che ho ritenuto necessarie e di aver ricevuto in merito risposte soddisfacenti.

Accetto dunque liberamente di partecipare alla sperimentazione, avendo compreso i rischi ed i benefici che sono implicati in questa partecipazione.

Acconsento (o non acconsento) che il dottor .... comunichi al mio medico di medicina generale dottor .... quanto a me spiegato sul significato della ricerca cui prenderò parte.

Sono stato informato che solo il personale medico autorizzato e le autorità regolatorie potranno avere accesso ai miei dati clinici, in ogni caso tutelati dal segreto professionale.

Sono stato inoltre informato del mio diritto ad avere libero accesso alla documentazione relativa alla sperimentazione ed alla valutazione espressa dal Comitato Etico.

Data _______ Firma del paziente ___________________________

Data _______ Firma del medico che ha informato il paziente ____________________

Policlinico, Via del Pozzo, 71 41100 Modena
Tel. +39-059-4222198-4224198 Fax. +39-059-4224231
e-mail: fabbri.leonardo@unimo.it
www.pneumologiamo.it

UNIVERSITÀ DEGLI STUDI
DI MODENA E REGGIO EMILIA

Università degli Studi di Modena e Reggio Emilia
DIPARTIMENTO INTEGRATO DI ONCOLOGIA,
EMATOLOGIA E PNEUMOLOGIA
(Direttore: Prof. Pier Franco Conte)

SERVIZIO SANITARIO REGIONALE
EMILIA-ROMAGNA
Azienda Ospedaliero-Universitaria di Modena

CLINICA DI MALATTIE DELL'APPARATO RESPIRATORIO
(Direttore: Prof. Leonardo M. Fabbri)

SCHEDA INFORMATIVA PER IL PAZIENTE

Struttura interessata: Clinica di Malattie dell'Apparato Respiratorio, Policlinico di Modena

Titolo della sperimentazione e numero di protocollo: "CONFRONTO FRA UN REGIME TERAPEUTICO BASATO SUI LIVELLI SIERICI DI PROCALCITONINA RISPETTO AD UN REGIME TERAPEUTICO STANDARD IN PAZIENTI OSPEDALIZZATI PER RIACUTIZZAZIONE DI BRONCOPNEUMOPATIA CRONICA OSTRUTTIVA"

Gentile Signora/e,

in questa Clinica universitaria intendiamo svolgere una ricerca medico-scientifica che si propone di migliorare la terapia dei pazienti ricoverati per una riacutizzazione di Broncopneumopatia Cronica Ostruttiva.

Per svolgere questa ricerca avremmo bisogno della Sua collaborazione. Prima che Lei prenda la decisione di accettare o rifiutare La preghiamo di leggere con attenzione quanto segue. La preghiamo di chiederci chiarimenti qualora non siano chiare le nostre spiegazioni.

Vogliamo informarLa che se decide di non partecipare allo studio riceverà comunque tutte le terapie normali previste per la Sua patologia ed i medici continueranno a seguirLa con la dovuta attenzione assistenziale.

Per facilitare la Sua decisione riassumiamo, nei punti seguenti, le informazioni che crediamo La possano riguardare direttamente e Le possano dunque essere utili nella decisione.

QUALI SONO LE TERAPIE ATTUALMENTE DISPONIBILI PER LA PATOLOGIA DA CUI LEI È AFFETTO ?

Poiché una potenziale causa di riacutizzazione della broncopneumopatia cronica ostruttiva (BPCO) è rappresentata dalle infezioni batteriche, che si caratterizzano di solito per un aumento del volume del catarro, per un colore giallastro dello stesso, e per una maggiore fatica a respirare, i pazienti affetti da questa malattia vengono molto spesso trattati con farmaci antibiotici, talvolta senza che ve ne sia la reale necessità con conseguente rischio di insorgenza di resistenze e soprattutto con il rischio di effetti collaterali determinati da tale trattamento antibiotico.

CHE COSA SI PROPONE QUESTO STUDIO?

Policlinico, Via del Pozzo, 71 41100 Modena
Tel. +39-059-4222198-4224198 Fax. +39-059-4224231
e-mail: fabbri.leonardo@unimo.it
www.pneumologiamo.it

Questo studio si propone quindi di valutare se il dosaggio di un ormone (la procalcitonina) nel sangue periferico sia in grado di guidare la durata della terapia antibiotica. Questa informazione è molto utile in quanto tale terapia si può associare ad effetti collaterali, ad allergie al farmaco ed a rischio di insorgenza di resistenza dei batteri.

PERCHÉ LE PROPONIAMO DI PARTECIPARE A QUESTO STUDIO?

Le proponiamo di partecipare a questo studio in quanto riteniamo che i criteri per stabilire l'efficacia della terapia antibiotica in pazienti affetti da riacutizzazione di BPCO non siano ottimali data la povertà di studi che li hanno definiti. Le verranno comunque somministrati 3 giorni di antibiotico, al fine di evitare la mancata somministrazione di tali farmaci in caso di necessità. Le verranno prelevati 3 campioni di sangue nei primi tre giorni per dosare l'ormone procalcitonina. Al termine del terzo giorno La assegneremo a uno di 2 gruppi in modo casuale: 1) se Lei venisse assegnato al gruppo cosiddetto di controllo, proseguirà l'antibiotico per altri 7 giorni (per un totale di 10 giorni di antibiotico). 2) Nel caso in cui Lei venisse assegnato al gruppo cosiddetto "sperimentale", la decisione se proseguire o meno il trattamento sarà basata sui livelli dell'ormone procalcitonina. Questo comporterà per Lei il vantaggio di non essere sottoposto inutilmente ad una terapia antibiotica non necessaria. In breve, se Lei venisse selezionato nel gruppo sperimentale avrà il vantaggio di vedere sospesa al più presto una terapia inutile per la sua malattia, mentre se Lei dovesse continuare a necessitare di tale terapia, questa Le verrà somministrata In modo usuale Ci teniamo a precisare che il medico potrà comunque decidere a propria discrezione cosa sia meglio per Lei, sulla base dell'andamento clinico della Sua malattia, anche in modo indipendente dai livelli di ormone procalcitonina.

Noi informeremo il Suo Medico Curante della Sua partecipazione allo studio e Lei sarà in ogni momento libero di chiedere informazioni a noi o a Lui/Lei al fine di evitare interferenze con eventuali altri farmaci che potrebbero esserLe stati prescritti. Nel caso Lei non ritenesse opportuno informare il curante Le chiediamo, prima di assumere qualunque altro farmaco, di comunicarlo al medico sperimentatore.

INDAGINI CUI SARÀ SOTTOPOSTO DURANTE LO STUDIO

Qualora Lei decidesse di partecipare allo studio, verrà seguito nell'ambito del protocollo dalla nostra Clinica per 6 mesi; al termine di tale studio Lei potrà continuare ad essere visitato presso la nostra Clinica ambulatorialmente. Nell'ambito dello studio, durante il ricovero Le saranno effettuati 4 prelievi di sangue per il dosaggio dell'ormone procalcitonina e sarà sottoposto ad altri esami a cui vengono sottoposti tutti i pazienti ricoverati per la Sua malattia.

Durante i 6 mesi Le chiederemo di tornare per una visita di controllo 4 volte: 10 giorni dopo il Suo ingresso in ospedale, e successivamente dopo 1, 3 e 6 mesi. Durante queste visite Le chiederemo di rispondere ad alcune domande, Le faremo eseguire le prove di funzionalità respiratoria, Le chiederemo un campione del Suo catarro e di eseguire un prelievo di sangue. Inoltre La contatteremo telefonicamente mensilmente, per informarci sulla Sua salute.

RISCHI DERIVANTI DALLA PARTECIPAZIONE ALLO STUDIO E TUTELA ASSICURATIVA PER IL PAZIENTE
La partecipazione allo Studio non comporta per Lei alcun rischio perché lo studio segue le linee guida per il trattamento della Sua malattia.

INTERRUZIONE DELLO STUDIO
Lei potrà ritirarsi dallo studio in ogni momento senza dover dare spiegazione alcuna.
I medici potranno in qualunque momento interrompere la sperimentazione e comunque, in questa ipotesi, gliene forniremo le motivazioni.

RISERVATEZZA DEI DATI PERSONALI
Ai sensi del decreto legislativo 196 del 30 giugno 2003 "Codice in materia di protezione dei dati personali" il paziente va informato che i suoi dati personali verranno raccolti ed archiviati elettronicamente e saranno utilizzati esclusivamente per scopi di ricerca scientifica.
Qualunque informazione raccolta durante la sperimentazione ed in particolare i dati personali saranno ritenuti di natura strettamente confidenziale e saranno trasferiti allo sponsor in maniera assolutamente anonima.
Il paziente avrà il diritto di conoscere quali informazioni saranno memorizzate e potrà aggiornare o modificare i dati erronei.
L'accesso a tali dati sarà protetto dallo sperimentatore (indicare quindi il titolare del trattamento dei dati).
Organismi governativi e personale medico addetto al monitoraggio ed alla verifica delle procedure potranno accedere all'archivio ma nell'assoluto rispetto delle disposizioni di cui decreto legislativo 196 del 30 giugno 2003 "Codice in materia di protezione dei dati personali"
Con la firma del modulo di consenso informato il paziente autorizza l'eventuale accesso di questi soggetti ai dati della sperimentazione.
I risultati dello studio potranno essere oggetto di pubblicazione ma l'identità del paziente sarà sempre segreta.

INFORMAZIONI CIRCA I RISULTATI DELLO STUDIO
Se Lei è interessato e lo richiede, alla fine della sperimentazione Le saranno comunicati i risultati dello studio in generale ed in particolare di quelli che La riguardano.

Per domande potrà contattare in ogni momento lo Sperimentatore Professor Leonardo Fabbri (telefono 059 422 2198) o il personale che partecipa allo studio (Dott. Luppi, tel. 059 422 5182; Dott.ssa Franco 059 422 2198).

La informiamo infine che questo protocollo sperimentale è stato redatto in conformità alle Norme di Buona Pratica Clinica ed è stato approvato dal Comitato Etico Provinciale di Modena

UNIVERSITÀ DEGLI STUDI
DI MODENA E REGGIO EMILIA

Università degli Studi di Modena e Reggio Emilia
DIPARTIMENTO INTEGRATO DI ONCOLOGIA,
EMATOLOGIA E PNEUMOLOGIA
(Direttore: Prof. Pier Franco Conte)

SERVIZIO SANITARIO REGIONALE
EMILIA-ROMAGNA
Azienda Ospedaliero - Universitaria di Modena

CLINICA DI MALATTIE DELL'APPARATO RESPIRATORIO
(Direttore: Prof. Leonardo M. Fabbri)

Nel caso Lei acconsenta a partecipare allo studio secondo le modalità sopra esposte, La preghiamo di firmare il modulo di consenso informato allegato.

Grazie della Sua collaborazione.

Prof. Leonardo M. Fabbri

Clinica di Malattie dell'Apparato Respiratorio
Tel. 059-4224227
E-mail: fabbri.leonardo@unimo.it

UNIVERSITÀ DEGLI STUDI
DI MODENA E REGGIO EMILIA

Università degli Studi di Modena e Reggio Emilia
DIPARTIMENTO INTEGRATO DI ONCOLOGIA,
EMATOLOGIA E PNEUMOLOGIA
(Direttore: Prof. Pier Franco Conte)

SERVIZIO SANITARIO REGIONALE
EMILIA ROMAGNA
Azienda Ospedaliero - Universitaria di Modena

CLINICA DI MALATTIE DELL'APPARATO RESPIRATORIO
(Direttore: Prof. Leonardo M. Fabbri)

Lettera per il medico curante

Modena, li

ALLA CORTESE ATTENZIONE DEL MEDICO CURANTE
DEL/LA SIG./RA

Egregio Collega,

Ti informiamo che il Tuo paziente, Sig./Sig.ra

____

ha dato il proprio assenso a partecipare al protocollo di ricerca istituzionale sul "CONFRONTO FRA UN REGIME TERAPEUTICO BASATO SUI LIVELLI SIERICI DI PROCALCITONINA RISPETTO AD UN REGIME TERAPEUTICO STANDARD IN PAZIENTI OSPEDALIZZATI PER RIACUTIZZAZIONE DI BRONCOPNEUMOPATIA CRONICA OSTRUTTIVA" condotto sotto l'egida dell'Università di Modena e Reggio Emilia.

Questo protocollo è stato approvato dal Comitato Etico Provinciale di Modena.

Come saprai, la broncopneumopatia cronica ostruttiva (BPCO) è una patologia causata principalmente dal fumo di sigaretta, che si caratterizza per la presenza di una progressiva e solo parzialmente reversibile ostruzione bronchiale solitamente associata a dispnea cronica, tosse ed espettorazione. Costituisce una rilevante causa di morbilità e mortalità e si caratterizza anche per la possibilità di riacutizzarsi. Le riacutizzazioni di BPCO costituiscono la prima causa di ricovero nelle Divisioni di Pneumologia sia in Italia che nel resto del mondo e sono causate principalmente da infezioni batteriche, virali e dall'inquinamento ambientale.

Le attuali linee guida consigliano di trattare la maggior parte dei pazienti affetti da riacutizzazione di BPCO anche con terapia antibiotica, nonostante il ruolo delle infezioni batteriche in tali riacutizzazioni sia tutt'altro che dimostrato. Infatti l'utilizzo di antibiotici in pazienti affetti da riacutizzazione di BPCO viene decisa sulla base di dati clinici che presentano spesso bassa sensibilità e sulla base di evidenze estremamente limitate. Alcuni marker diagnostici, fra cui la procalcitonina (pro-ct), possono essere utilizzati quale guida nel selezionare i pazienti affetti da riacutizzazione di BPCO che necessitano effettivamente di terapia antibiotica.

La pro-ct è un peptide precursore dell'ormone calcitonina, la quale è stata dosata in elevate concentrazioni in pazienti affetti da grave infezione batterica, ma che si mantiene a concentrazioni molto basse in pazienti affetti da infezione virale. La dimostrazione dell'efficacia di uno specifico *marker* biologico – quale la pro-ct – nell'identificazione della causa batterica di riacutizzazione di BPCO, potrebbe pertanto rappresentare un'importante informazione al fine di ridurre l'uso

Policlinico, Via del Pozzo, 71 41100 Modena
Tel. +39-059-4222198-4224198 Fax. +39-059-4224231
e-mail: fabbri.leonardo@unimo.it
www.pneumologiamo.it

improprio di antibiotici in corso di riacutizzazione di BPCO, riducendo inoltre i costi diretti ed indiretti degli antibiotici dovuti agli effetti collaterali da essi causati, quali la resistenza agli antibiotici, l'insorgenza di tossicità di allergia, ecc.

L'obiettivo primario dello studio è confrontare un regime di trattamento antibiotico effettuato sulla base dei livelli sierici di procalcitonina rispetto ad un regime di trattamento antibiotico di 10 giorni come consigliato dalle attuali linee guida, quando siano presenti segni di riacutizzazione infettiva di malattia (aggravamento della dispnea, di un aumento del volume dell'escreato e della sua purulenza).

Ti ringraziamo per la gentile collaborazione e rimaniamo a Tua disposizione per qualsiasi ulteriore chiarimento ritenessi utile.

Prof. Leonardo M. Fabbri

Clinica di Malattie dell'Apparato Respiratorio
Tel. 059-4222198
E-mail: fabbri.leonardo@unimo.it
