# Supplementary material for: Antibiotic Treatment of Severe Exacerbations of Chronic Obstructive Pulmonary Disease with Procalcitonin: A Randomized Noninferiority Trial
Source: PLoS One. 2015 Mar 11;10(3):e0118241. doi: 10.1371/journal.pone.0118241 (PMC4356612; doi:10.1371/journal.pone.0118241)
Supplement: S1 Protocol — (PDF) [file pone.0118241.s003.pdf]

UNIVERSITÀ DEGLI STUDI
DI MODENA E REGGIO EMILIA

Università degli Studi di Modena e Reggio Emilia
DIPARTIMENTO DI ONCOLOGIA, EMATOLOGIA E PNEUMOLOGIA
Direttore: Prof. Pier Franco Conte

SERVIZIO SANITARIO REGIONALE
EMILIA-ROMAGNA
Azienda Ospedaliero - Universitaria di Modena

CLINICA DI MALATTIE DELL'APPARATO RESPIRATORIO
Direttore: Prof. Leonardo M. Fabbri

Study Protocol

COMPARISON OF A SERUM PROCALCITONIN (PRO-CT) GUIDED TREATMENT PLAN WITH THE STANDARD GUIDELINE RECOMMENDED ANTIBIOTIC TREATMENT PLAN FOR PATIENTS HOSPITALIZED WITH A DIAGNOSIS OF EXACERBATION OF COPD

Version – September 2, 2006

INDEPENDENT PROJECT APPROVED BY AGENZIA ITALIANA DEL FARMACO ON 24/3/2006

Policlinico, Via del Pozzo, 71 41100 Modena
Tel. +39-059-4222198-4224198 Fax. +39-059-4224231
e-mail: fabbri.leonardo@unimo.it
www.pneumologiamo.it

TABLE OF CONTENTS

Author's signature page 4
List of study centers 5
Signature page for Investigators 10
List of key staff, Coordinating Center of Modena 11
STUDY SCHEDULE OF VISITS AND PROCEDURES 13
1 INTRODUCTION 14
1.1 Background information 14
1.2 Rationale 16
2 STUDY OBJECTIVES 17
2.1 Primary Objective 17
2.2 Secondary Objectives 17
3 STUDY DESIGN 17
4 SUBJECT SELECTION CRITERIA 20
4.1 Number of Subjects 20
4.2 Inclusion criteria 20
4.3 Exclusion criteria 21
4.4 Withdrawal criteria 21
5 TREATMENT OF SUBJECTS 21
5.1 Treatment Assignment and blinding 21
5.2 Treatment Compliance 21
5.3 Concomitant Medication 22
6 CLINICAL PROCEDURES 22
6.1 Central Laboratory Procedures 27
7 ENDPOINTS AND SAFETY EVALUATIONS 27
7.1 Study endpoints 27
7.2 Safety Evaluation / Adverse Event 28
8 STATISTICAL METHODOLOGY 28
8.1 Sample size 28
8.2 Data Analysis 28
8.3 Safety analysis 29
9 DIRECT ACCESS TO SOURCE DATA 30

Policlinico, Via del Pozzo, 71 41100 Modena
Tel. +39-059-4222198-4224198 Fax. +39-059-4224231
e-mail: fabbri.leonardo@unimo.it
www.pneumologiamo.it

10 ETHICS........ 30
11 STUDY MONITORING 30
12 REFERENCES 30
ANCILLARY PROJECTS 33

Policlinico, Via del Pozzo, 71 41100 Modena
Tel. +39-059-4222198-4224198 Fax. +39-059-4224231
e-mail: fabbri.leonardo@unimo.it
www.pneumologiamo.it

UNIVERSITÀ DEGLI STUDI DI MODENA E REGGIO EMILIA

Università degli Studi di Modena e Reggio Emilia
DIPARTIMENTO DI ONCOLOGIA, EMATOLOGIA E PNEUMOLOGIA
Direttore: Prof. Pier Franco Conte

SERVIZIO SANITARIO REGIONALE
EMILIA-ROMAGNA
Azienda Ospedaliero-Universitaria di Modena

CLINICA DI MALATTIE DELL'APPARATO RESPIRATORIO
Direttore: Prof. Leonardo M. Fabbri

Author's signature page

Prof. L. Fabbri ________________________________ ____02/09/06_______

Signature Date

Policlinico, Via del Pozzo, 71 41100 Modena
Tel. +39-059-4222198-4224198 Fax. +39-059-4224231
e-mail: fabbri.leonardo@unimo.it
www.pneumologiamo.it

List of study centers

1. Coordinating center

Prof. Leonardo M. Fabbri
Clinica di Malattie dell'Apparato Respiratorio
Università degli Studi di Modena e Reggio Emilia
Via del Pozzo 71, 41100 MODENA
Tel. +39-0594222198, -4198, -4227
Fax : +39-0594224231
Cell. +39-3482945522
Email: fabbri.leonardo@unimo.it

2. Dr. Stefano Calabro
Ospedale di Bassano del Grappa
Divisione di Pneumologia,
Via Dei Lotti, 40
36061 Bassano del Grappa
Tel. 0424 888515
Fax: 0424 888827
Cell. 3484742903
Email: stefano.calabro@tin.it

3. Prof. Giorgio Walter Canonica
Servizio Allergologia e Immunologia Clinica
Dipartimento di Medicina Interna
Università degli Studi di Genova
Viale Benedetto XV, 6
16132 Genova
Tel. 010 35 38 933 – 931
Fax: 010 35 38 904
Cell. 335-6939299
E-mail: gcanonica@qubisoft.it

4. Dr. Michele Ciccarelli
Istituto Clinico Humanitas
Via Manzoni 56
20089 Rozzano
Milan, Italy
Tel. 02 8224.4531
Email: michele.ciccarelli@humanitas.it

5. Dr. Marco Confalonieri
Struttura Complessa Pneumologia
Ospedale di Cattinara
Strada di Fiume 447
34100 Trieste
email: marco.confalonieri@aots.sanita.fvg.it

6. Prof. Nunzio Crimi
Istituto di Tisiologia e Malattie dell'Apparato Respiratorio
Via Passo Gravina, 187
95125 CATANIA
Tel. 095 759 35 35
Cell. 333-7824161
E-mail: crimi@mbox.unict.it

7. Dr. Giulio Donazzan
Ospedale Generale Regionale
Divisione di Pneumologia
Via L. Boehler, 5
39100 Bolzano
Tel. 0471-908317
Fax: 04718781
Email: gdonazzan@qubisoft.it

8. Prof.ssa Maria Pia Faschino Barbaro
Clinica di Malattie dell'Apparato Respiratorio
Università degli Studi di Foggia
Ospedale Pneumologico d'Avanzo
Viale degli Aviatori, 1
Foggia
Tel. 0881/617832
Fax: 0881/733040
Email: mfoschino@qubisoft.it

9. Prof. Serafino Marsico
Istituto di Tisiologia e Malattie dell'Apparato Respiratorio
I Facoltà di Medicina e Chirurgia
Ospedale "V. Monaldi" – Camaldoli
80131 Napoli
Tel. 081 54 53 017
Tel. 081 7062365 (segretaria)
Cell. 329-6507405

Fax: 081 54 63 812
Email: serafino.marsico@unina2.it

10. Prof. Dario Olivieri
Direttore dell'Istituto di Clinica
delle Malattie dell'Apparato Respiratorio
Ospedale "G. Rasori"
Via Rasori, 10
43100 Parma
Tel. 0521 29 05 77
Fax: 0521 29 26 15
E-mail: olivieri@unipr.it

11. Prof. Pierluigi Paggiaro
Malattie dell'Apparato Respiratorio
Università degli Studi di Pisa Ospedale Cisanello
Via Paradisa, 2
56100 Pisa
Tel. 050-995266
Fax: 050-580124
Cell: 349-4340001
Email: paggiaro@qubisoft.it

12. Prof. Alberto Papi
Sezione di Malattie dell'Apparato Respiratorio
Università degli Studi di Ferrara
Via Savonarola, 9
44100 Ferrara
Tel. 0532-210420
Fax: 0532-210297
email: ppa@unife.it

13. Prof. Massimo Pistolesi
Sezione Fisiopatologia Respiratoria
Dipartimento di Fisiopatologia Clinica
V.le Morgagni, 85
50134 Firenze
Tel. 055 41 31 83 – 42 77 516
Fax: 055 42 23 202
Cell. 329-6117036
E-mail: massimo.pistolesi@unifi.it

14. Prof. Ernesto Pozzi

Direttore
Clinica di Malattie dell'Apparato Respiratorio
IRCCS Policlinico S Matteo
Via Taramelli 5
27100 PAVIA
Tel. 0382/422232
Fax: 0382/422267
Cell. 348-6717811
Email: tisiolmm@unipv.it

15. Dr. Andrea Rossi
U.O. di Pneumologia
Ospedali Riuniti di Bergamo
Largo Barozzi, 1
24128 Bergamo
Tel. 035-269714
Fax: 035-266825
E-mail: arossi@ospedaliriuniti.bergamo.it

16. Dr. Antonio Spanevello
Divisione di Pneumologia
Fondazione S. Maugeri
Istituto Scientifico di Cassano
Via per Mercadante Km. 2
70020 Cassano delle Murge Bari
Tel. 080-7814111/7814228
Fax: 080-7814310
Cell. 349-7177858
Email: aspanevello@fsm.it

17. Dr. Luigi Zucchi
Divisione di Pneumologia
Ospedale S. Maria Nuova
V.le Risorgimento, 80
42100 Reggio Emilia
Tel. 0522-216156/216157
Fax: 0522-296182
Cell. 335-452642
Email: Luigi.Zucchi@asmn.re.it

18. Prof. Renzo ZUIN
Cattedra di Malattie Respiratorie
Divisione di Pneumologia

Via Giustiniani, 3
35128 Padova
Tel. 049 82 13 732 – 700
Fax: 049 82 13 701
Cell. 340-2520314
E-mail: divpneumo@tiscalinet.it

Signature page for Investigators

STUDY CENTRE :

Hospital : ……………………………………..
……………………………………..
……………………………………..
……………………………………..

Principal Investigator: Prof./Dr: ……………………………..……

I have read this protocol and I agree to conduct the study according to this protocol, to the ethical principles for Medical Research Involving Human Subjects adopted by the Helsinki Declaration and to GCP. Any changes in procedure will only be made if necessary to protect the safety, rights or welfare of subjects.

I agree to conduct in person or to supervise the study. I agree to ensure that all who assist me in the conduct of the study have access to the study protocol and are aware of their obligations.

Investigator's Signature: ________________________ Date : |____|____|____|

Policlinico, Via del Pozzo, 71 41100 Modena
Tel. +39-059-4222198-4224198 Fax. +39-059-4224231
e-mail: fabbri.leonardo@unimo.it
www.pneumologiamo.it

List of key staff, Coordinating Center of Modena

- Professor Leonardo M. Fabbri, Clinica di Malattie dell'Apparato Respiratorio, Università degli Studi di Modena and Reggio Emilia, Via del Pozzo, 71, Modena, Italy.
  Phone 059 422 2198-4198, fax 059 422 4231

- Professore Luca Richeldi, Clinica di Malattie dell'Apparato Respiratorio, Università degli Studi di Modena and Reggio Emilia, Via del Pozzo, 71, Modena, Italy.
  Phone 059 422 2655, fax 059 422 4231

- Dottor Fabrizio Luppi, Clinica di Malattie dell'Apparato Respiratorio, Università degli Studi di Modena and Reggio Emilia, Via del Pozzo, 71, Modena, Italy.
  Phone 059 422 5182, fax 059 422 4231

- Dr.ssa Francesca Franco, Clinica di Malattie dell'Apparato Respiratorio, Università degli Studi di Modena and Reggio Emilia, Via del Pozzo, 71, Modena, Italy.
  Phone 059 422 2198, fax 059 422 4231

- Dr.ssa Maria Rosaria De Carlo, Clinica di Malattie dell'Apparato Respiratorio, Università degli Studi di Modena and Reggio Emilia, Via del Pozzo, 71, Modena, Italy.
  Phone 059 422 2198, fax 059 422 4231

- Margherita Mosconi, CROM, phone 045 8222830, mobile 335 7698380, margherita.mosconi@crom.it

- Dr. Bruno D'Agostino, Ricercatore presso la Seconda Università degli Studi di Napoli (SUN) Facoltà di Medicina e Chirurgia, Dipartimento di Medicina Sperimentale, Sez. di Farmacologia "L.Donatelli", Via Costantinopoli 16, 80138 Napoli, phone: 0815667651 or 0815667533, Mobile:3472601620, Email: bruno.dagostino@unina2.it

- Alberto Mantovani, Professor, Scientific Director, Istituto Clinico Humanitas, Via Manzoni 56, 20089 Rozzano, Milan, Italy, phone +39- 02 8224.2444, (PA Antonella Palmero and secretariat : +39- 02 8224.2445/2446), Fax +39- 02 8224.5101, E-mail alberto.mantovani@humanitas.it

- Prof. Mario Plebani, Laboratorio Analisi, Policlinico Universitario di Padova, Via Giustiniani, 2, 35128 Padova, phone 049-8212792, fax 049-663240, Email: mario.plebani@unipd.it

Policlinico, Via del Pozzo, 71 41100 Modena
Tel. +39-059-4222198-4224198 Fax. +39-059-4224231
e-mail: fabbri.leonardo@unimo.it
www.pneumologiamo.it

- Protocol Summary

Exacerbations of chronic obstructive pulmonary disease (COPD) are the first cause of admission to pulmonary departments in Italy and worldwide. Guidelines recommend treating most patients hospitalized for exacerbations of COPD with antibiotics, even if the role of bacterial infections is often uncertain and the effect of antibiotics small. The recommendation to use antibiotics is guided by clinical signs and symptoms that have an insufficient diagnostic accuracy, whereas serum biomarkers, eg procalcitonin (pro-ct), may guide the selection of COPD patients who need antibiotic treatment. The main aim of our study is to investigate whether antibiotics can be safely stopped after 3 days or continued for 10 days according to a pro-ct-guided algorithm in patients hospitalized for exacerbations of COPD for whom guidelines recommend 3-10 days antibiotic treatment based on the presence of increased dyspnoea, sputum and purulence. The study is designed to assess the non inferiority of the pro-ct guided plan as compared to the standard guideline recommended plan. The pro-ct guided withholding of antibiotics is viewed as an experimental intervention associated with less antibiotic-associated complications, eg antibiotic resistance and drug-related side-effect and lower costs. The proposed study is a prospective, randomised controlled, single-blinded intervention trial comparing the standard with a pro-ct guided antibiotic treatment plan. Patients will be recruited from the Pulmonary Departments of 18 University or City Hospitals in Italy, starting October 2006 and continuing until April 1, 2008. Results expected by end of 2008. The 200 patients randomised to the standard non pro-ct guided antibiotic treatment plan will continue antibiotics for 10 days, whereas the 200 patients randomised to the pro-ct-guided antibiotic treatment plan, will continue for 10 days or stop antibiotics on day 3 depending on pro-ct levels measured at admission, day 1 and day 2. Serum pro-ct will be measured in a central laboratory. Patients will be examined at admission, discharge, 10 days, 1, 3 and 6 months. A telephone interview will be obtained at 2, 4 and 5 months. The primary outcome of the study will be the rate of exacerbations. Secondary outcomes will be hospital readmission, admission to ICU, change in FEV1, length of the hospital stay, and death. The sample size was estimated according to the primary outcome of the study. This study will be guided by a Steering Committee and monitored by an independent Safety Committee.

Policlinico, Via del Pozzo, 71 41100 Modena
Tel. +39-059-4222198-4224198 Fax. +39-059-4224231
e-mail: fabbri.leonardo@unimo.it
www.pneumologiamo.it

STUDY SCHEDULE OF VISITS AND PROCEDURES

	Hospitalization						
	**Visit 1**	**Visit 2**	**Visit 3**	**Visit 4**	**Visit 5**	**Visit 6**	**Visit 7**
	**Day 0**	**Day 1**	**Day 2 Randomisation**	**At hospital discharge e/o day 10**	**Day 30**	**Day 90**	**Day 180**
**Informed Consent**	x						
**Medical history**	x						
**Physical examination**	x	x	x	x	x	x	x
**Vital signs**	x	x	x	x	x	x	x
**Body temperature**	x	x	x	x			
**Concomitant medication**	x	x	x	x	x	x	x
**Pulmonary x-ray**	x						
**Spirometry**	x			x	x	x	x
**Sputum collection**	x			x	x		x
**ECG**	x						
**Inclusion / exclusion criteria**	x						
**Questionnaire for quality of life**	x			x	x	x	x
**Pharmaco-economy questionnaire**	x			x	x	x	x
**Haematology**	x		x (if abnormal at day 0)	x		x	
**Blood chemistry**	x		X (if abnormal at day 0)	x		x	
**CRP, SAA and RANTES**	x	x	x	x	x	x	x
**PTX3 and IL-1 decoy**	x			x		x	
**Pro-ct levels**	x	x	x	x	x	x	x
**Arterial blood gases**	x	x	x	x	x	x	x
**Treatment compliance**	x	x	x	x			
**Adverse event**	x	x	x	x	x	x	x

Policlinico, Via del Pozzo, 71 41100 Modena
Tel. +39-059-4222198-4224198 Fax. +39-059-4224231
e-mail: fabbri.leonardo@unimo.it
www.pneumologiamo.it

1 INTRODUCTION

1.1 Background information

Chronic obstructive pulmonary disease (COPD) is a chronic disease caused mainly by cigarette smoking that is characterized by progressive and poorly reversible airflow limitation that is usually associated with chronic dyspnoea, cough and sputum (1,2). COPD is a leading cause of morbidity and mortality worldwide and results in an economic and social burden that is both substantial and increasing (3).

COPD often exacerbates. COPD exacerbations are defined as a change in the patient's chronic respiratory symptoms sufficient to warrant a change in management (1,2). COPD exacerbations are a frequent cause of hospital admission and severely affect quality of life and prognosis. Mortality rates during hospitalizations are around 10% and during the year following hospitalization may be as high as 40% (4).

In Italy the 2001 mortality for COPD was 17.403, a number stable in the last 10 years (5).

The 2002 hospital admission caused by COPD (DRG 88) were 120.188, the majority (97.473) in patients > 65 years. If added to patients admitted to hospital for acute respiratory failure and pulmonary oedema (DRG 87) and of respiratory failure requiring mechanical ventilation (DRG 475), there would be >150.000 hospital admissions caused COPD exacerbation (6). The total estimated cost of DRG 88 and of the portion of DRG 87 and 475 that can be attributed to COPD exacerbation is > than € 500.000.000/year (6,7).

The main causes of COPD exacerbation are believed to the bacterial and viral infections and/or air pollution. More than 50% of COPD exacerbation are associated with viral infection, the majority of which are due to rhinovirus (4,8). The lower airways of 25 to 50% of COPD patients are colonized by bacteria, especially non capsulated Haemophilus influenzae, Streptococcus pneumoniae, and Moraxella catarrahalis (9). The predominant bacteria recovered in the lower airways of patients with mild exacerbation are H. influenzae, S. pneumoniae and M. catarrahalis, whereas in severe COPD requiring mechanical ventilation the predominant bacteria are gram negative bacilli, and P. aeruginosa is more frequent (4). Although bacteria can be isolated from the sputum of the majority of the patients with exacerbations of COPD, whether this finding represents colonization or infection remains controversial. The uncertain role of bacterial infections is also suggested by the limited evidence that antibiotics are effective in the treatment of COPD exacerbations. The strongest evidence available remains one randomized controlled trial published in 1987 that showed that the beneficial effect of antibiotics is limited to COPD patients who presented with an increase in all three of cardinal symptoms, i.e. dyspnoea, sputum volume, sputum purulence afterward named the "Anthonisen's criteria" (9). There was also some benefit in those patients with an increase in only two of these cardinal symptoms. A study on non-hospitalized patients with exacerbation of COPD showed a relationship between the purulence of the sputum and the presence of bacteria, suggesting that these patients should be treated with antibiotic if they also have at least one of the other two cardinal symptoms (dyspnoea or sputum volume) (10). However, these criteria for exacerbation of COPD have not been validated in other interventional trails and in fact the

Policlinico, Via del Pozzo, 71 41100 Modena
Tel. +39-059-4222198-4224198 Fax. +39-059-4224231
e-mail: fabbri.leonardo@unimo.it
www.pneumologiamo.it

overall sensitivity and specificity of signs and symptoms for exacerbations of COPD requiring antibiotic therapy is poor (10). Thus exacerbations of COPD are often treated with antibiotics without evidence of clinically relevant bacterial disease. Based on the current available albeit limited evidence, guidelines (1,2,11) recommended antibiotics for:

- patients with exacerbation of COPD with all 3 cardinal symptoms (increase of dyspnoea, and sputum volume and sputum purulence)
- patients with exacerbation of COPD with 2 of the 3 cardinal symptoms, if increased purulence of sputum is one of the 2 symptoms
- patients with a severe exacerbation of COPD that requires invasive mechanical ventilation (invasive and non-invasive).

Regarding hospitalized patients, in addition to the treatment with bronchodilators and systemic steroids, guidelines strongly recommend antibiotics treatment for most exacerbations of COPD even if their effect has not been properly investigated.

In fact, of 9 placebo-controlled trials included in one meta-analysis, 5 did not show a significant benefit in the group treated with antibiotics, and 4 were conducted in hospitalized patients. The overall results favored antibiotics, but the benefit was clinically marginal in all studies. Although inpatients seemed to benefit more from antibiotics than outpatients, extrapolation of the marginal effect seen in this meta-analysis (12), to properly diagnosed and characterized patients is not possible. Nonetheless, use of antibiotics in patients hospitalized with COPD exacerbation remains usual practice, although this treatment is not at all based on evidence.

In particular whether antibiotic treatment is effective in patients with COPD exacerbations characterized according to above described clinical criteria (dyspnoea, sputum and purulence) has never been examined in a properly designed and powered prospective study. The only properly designed and conducted randomized placebo controlled study was performed in patient admitted to the intensive care unit because of an exacerbation of COPD associated with acute respiratory failure requiring mechanical ventilation (13), and showed that treatment with antibiotics (oral moxafloxacin for 10 days) reduced the number of deaths, the duration of mechanical ventilation and hospital stay. However, the positive results obtained in this study can not be extrapolated to all patients hospitalized for COPD exacerbations, firstly because only one part of these patients are managed in the Intensive Care Unit and require mechanical ventilation, secondly because symptoms and particularly purulent sputum were not considered in the inclusion criteria.

In conclusion, the few data available and current concern about use of antibiotics and their contribution to selection of resistant bacteria, show that a properly designed and powered study is needed to investigate the role of antibiotics in the management of these patients. To limit antibiotic use, rapid and accurate differentiation of clinically relevant bacterial lower respiratory tract infections from other, mostly viral or non-infections, causes is pivotal. After obtaining a patient's medical history, physical examination, laboratory test, and chest radiograph, the clinician is often left with diagnostic uncertainty, because signs and symptoms of bacterial and viral infections widely overlap (14).

Policlinico, Via del Pozzo, 71 41100 Modena
Tel. +39-059-4222198-4224198 Fax. +39-059-4224231
e-mail: fabbri.leonardo@unimo.it
www.pneumologiamo.it

Also, bacteria can be isolated from sputum in up 50% of patients with acute exacerbation of COPD, but whether this finding represents colonization or infection in controversial (14), also because viruses can also be isolated in a similar proportion, and in up to 25% of patients with COPD exacerbation there is neither evidence of bacteria nor of viruses (14,15).

In conclusion the prescription of antibiotics in COPD exacerbation is guided by clinical signs and symptoms with very poor scientific evidence.

The majority of routinely used clinical parameters have insufficient diagnostic predictive value to identify patients with bacterial infection requiring antibiotics, whereas some biomarkers, e.g. C-reactive protein (CRP) and procalcitonin (pro-ct), might provide a more adequate discrimination between patients with and without bacterial respiratory infections requiring antibiotic therapy (16).

1.2 Rationale

Pro-ct is a precursor peptide from the hormone calcitonin. After translation from calcitonin-messenger RNA (mRNA), pro-ct is cleaved enzymatically into smaller peptides (17). Circulating amounts of calcitonin precursor, particularly pro-ct, are increased in severe bacterial infections, but remains fairly low in viral infection and non-specific inflammatory diseases. Previous studies have established the superior diagnostic accuracy of pro-ct in severe infection, and pro-ct guidance substantially reduces antibiotic use in lower respiratory tract infection without compromising clinical outcomes (16). According to a recently completed study:

- the large majority of patients with COPD exacerbations are treated with antibiotics
- the Anthonisen's criteria marginally reduce the use of antibiotics
- a pro-CT guided antibiotic treatment almost halves the use of antibiotics.

C reactive protein also increases during bacterial infection and COPD exacerbations but, at variance with pro-ct:

- its increase is delayed (17)
- is blunted in steroid treated patients with bacterial infections (18)
- is less sensitive in the early phase and less specific in the late phase of a bacterial infection, thus less adequate to guide the initiation and duration of antibiotics (19)

which limits its clinical usefulness.

Other biomarkers (interleukin (IL)-6, IL-8, neopterin, TNFα, or soluble triggering receptor expressed on myeloid cells have not been adequately examined in COPD patients.

The validation of a specific biomarker of bacterial respiratory infection such as pro-ct would provide a valuable information to the Italian Agency of Drug to reduce the inappropriate use of antibiotics for exacerbation of COPD, and would help the Italian Ministry of Health to reduce both the direct cost and the indirect costs of antibiotics due to side effects (resistance, diarrhea, allergy, etc).

Policlinico, Via del Pozzo, 71 41100 Modena
Tel. +39-059-4222198-4224198 Fax. +39-059-4224231
e-mail: fabbri.leonardo@unimo.it
www.pneumologiamo.it

2 STUDY OBJECTIVES

2.1 Primary Objective

The main objective of study is to investigate whether antibiotics can be safely stopped at 3 days or continued for 10 days according to a pro-ct-guided plan in patients hospitalized for exacerbation of COPD requiring antibiotic treatment and associated with increased dyspnoea, increased sputum volume and sputum purulence.

The study is designed to assess the non inferiority of the pro-ct guided plan as compared to the standard 10 days antibiotic treatment plan recommended by guidelines. All patients will receive 3 days of antibiotic treatment regardless of the randomization arm, as recommended by guidelines.

2.2 Secondary Objectives

Secondary objectives are:

1. to estimate the direct and indirect cost of hospitalized exacerbation of COPD and the cost/benefits of experimental pro-ct guided antibiotic treatment plan;
2. to determine whether the pro-ct guided continuation of antibiotic treatment for 10 days is superior to the standard non pro-ct guided antibiotic treatment of 10 days because pro-ct identifies patients whose exacerbation are caused by bacterial infection;
3. to determine the rate of antibiotic-associated side effects (i.e. frequency of gastrointestinal symptoms like diarrhea or nausea, allergy, other drug-specific adverse reaction);
4. to estimate, through pro-ct, the proportion of exacerbation of COPD defined with the "Anthonisen's" clinical criteria that need to start antibiotics in the first place
5. to investigate the heterogeneity of COPD exacerbation, as the symptoms of COPD exacerbation (increasing dyspnoea and cough and sputum) are non specific, and they may be often due to other diseases (acute heart failure, pneumonia, pulmonary thromboembolism)
6. to compare the predictive value of recommended clinical criteria with traditional (eg. CRP, leukocyte, fever, etc.) and novel (acute phase proteins, prohormones) biomarkers (Ancillary project 2)
7. to determine the relationship between serum pro-ct and sputum bacterial/viral load/etiology (Ancillary project 3).

3 STUDY DESIGN

This is a prospective, randomized, controlled, single-blinded interventional trail comparing the standard antibiotic therapy recommended by current guidelines (1,2), with a pro-ct guided antibiotic treatment for patient with COPD exacerbation admitted to the respiratory units of 18 sites (University or City Hospital).

Policlinico, Via del Pozzo, 71 41100 Modena
Tel. +39-059-4222198-4224198 Fax. +39-059-4224231
e-mail: fabbri.leonardo@unimo.it
www.pneumologiamo.it

Only COPD patients hospitalized because of an exacerbation characterized by increased dyspnoea, sputum and purulence, who according to guideline require treatment with antibiotic, will be recruited starting October 2006 and continuing until April 2008.

The eligible patients will be randomly assigned to the standard or to the pro-ct guided antibiotic treatment plan according to a 1:1 computer-generated scheme. Randomization will be stratified according to hospital.

Initially all patients will start antibiotics at admission to hospital and pro-ct will be measured at admission, day 1 and 2. All pro-ct samples will be sent by courier on day 2 to the central laboratory (Central laboratory, University of Padova), where serum pro-ct will be measured.

Patients randomized to the standard non pro-ct guided antibiotic treatment plan, will continue antibiotics for 10 days. Patients randomized to the pro-ct guided antibiotic treatment plan, will continue or stop antibiotics on day 3 depending on pro-ct levels, using cut-offs previously validated (16).

Policlinico, Via del Pozzo, 71 41100 Modena
Tel. +39-059-4222198-4224198 Fax. +39-059-4224231
e-mail: fabbri.leonardo@unimo.it
www.pneumologiamo.it

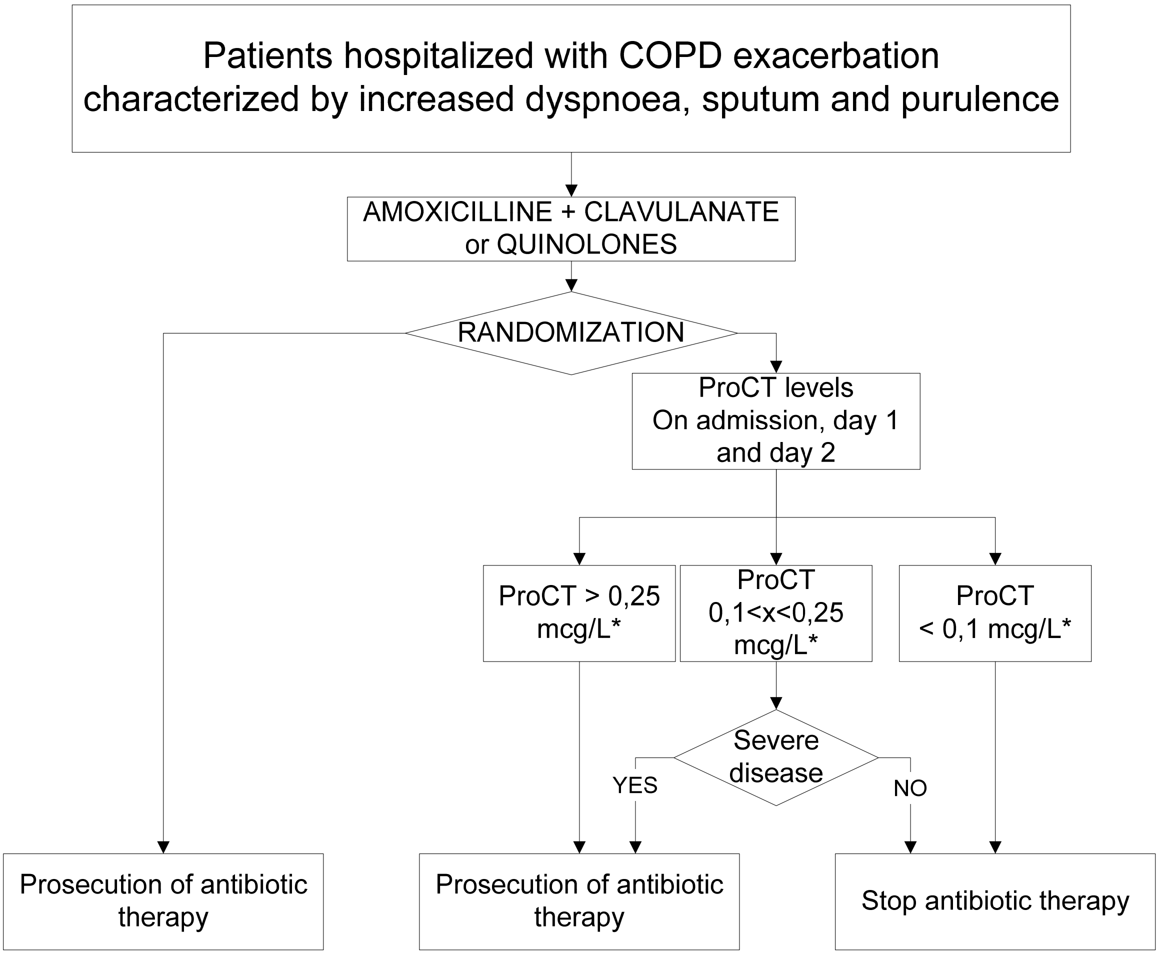

* On admission, day 1 and day 2

In particular they will:

- continue antibiotics for 10 days if a single pro-ct value is 0.25 mcg/L or greater, that will be judged suggestive for the presence of bacterial infection
- continue antibiotics from day 3 to day 10 if 1 or more pro-ct values is/are 0.1 to 0.25 mcg/L, indicating possible bacterial infection, PLUS acute respiratory failure or clinical instability
- stop antibiotics on day 3, if 1 ore more pro-ct values is/are between 0.1 to 0.25 mcg/L, indicating possible bacterial infection, BUT the patient has not acute respiratory failure or clinical instability

Policlinico, Via del Pozzo, 71 41100 Modena
Tel. +39-059-4222198-4224198 Fax. +39-059-4224231
e-mail: fabbri.leonardo@unimo.it
www.pneumologiamo.it

- stop antibiotics if all pro-ct values is/are < 0.1 mcg/L or if there is a consistent trend to normalization of pro-ct with the last value < 0.1 mcg/L, that will be judged suggestive for absence of bacterial infection.

The final decision on the antibiotic treatment will be left to the investigator according to guidelines (1,2). Likewise, other diagnostic procedures, concomitant therapeutic regimen or change of regimen and final decision to initiate antibiotic treatment will be, in all cases, left to the discretion of investigator. In the pro-ct group all investigator will be advised to follow the antibiotic treatment algorithm based on the pro-ct levels. Thereafter, patients will be prospectively followed up during hospitalization and after hospital discharge.

4 SUBJECT SELECTION CRITERIA

4.1 Number of Subjects

All patients admitted to the participating hospital respiratory units for exacerbation of COPD will be potential subjects. The Investigators will define and classify the severity of COPD according to the Global Initiative for Chronic Obstructive Lung Disease guidelines (http://www.goldcopd.com).

A total of 400 patients (200 for arm) hospitalized for COPD exacerbation will be enrolled in the study. For the sample size see chapter 8.1.

4.2 Inclusion criteria

- Male and female patients who give written informed consent
- Age: adults >18 years old
- COPD diagnosis according to GOLD guidelines: FEV1/FVC <70% with FEV1<80% of predicted. If a COPD diagnosis is not evident from previous spirometric exams of the patient, a new spirometry will be obtained within 3 days of admission and a diagnosis of COPD will be made if all of the following conditions are true: 1) FEV1/FVC <70% with FEV1 <80% of predicted; 2) history of cigarette smoking; 3) exclusion of an asthma diagnosis. At the end of the study (i.e. at 6 months) a new spirometric exam will be obtained from these patients to confirm the new diagnosis of COPD made at admission.
- Diagnosis of COPD exacerbation:
    - defined as acute-onset dyspnoea and/or cough associated with increased purulent sputum production (Anthonisen's criteria)
    - requiring, according to guidelines (GOLD 2005), treatment with antibiotic
    - requiring hospitalization

Policlinico, Via del Pozzo, 71 41100 Modena
Tel. +39-059-4222198-4224198 Fax. +39-059-4224231
e-mail: fabbri.leonardo@unimo.it
www.pneumologiamo.it

4.3 Exclusion criteria

- Female subjects: pregnant, lactating mother or lack of efficient contraception in a subject with child-bearing potential (e.g. contraceptive methods other than oral contraceptives, IUD, tubal ligature)
- Diagnosis of asthma
- Coexisting medical conditions: unstable concomitant cardiovascular, renal, hepatic, gastrointestinal, neurological, endocrine, metabolic, musculo-skeletal, neoplastic, respiratory or other clinically significant disease (patients with stable and well controlled hypertension or diabetes maybe included in the study)
- Clinical significant laboratory abnormalities indicating unstable concomitant disease
- Patients in whom survival for at least 1 year is unlikely
- Inability to give informed consent

4.4 Withdrawal criteria

Patients have the right to withdraw from the study at any time for any reason, including personal reasons. The Investigators have also the right to withdraw patients from the study in the event of intercurrent illness that necessitates pharmacological treatment with a disallowed drug, in case of an adverse event, treatment failure, protocol violations, poor compliance, pregnancy or any other reason.

5 TREATMENT OF SUBJECTS

5.1 Treatment Assignment and blinding

On day 2 each eligible patient will be randomly assigned to one of the two treatment plans.

The randomization will be performed via website. The allocation schedule will be computer generated with random numbers and prepared before the study begins by an independent randomization center, which will not reveal the allocation sequence to the Investigators. After baseline assessments, the Investigators will connect to the web site and, in case of eligibility, patients will be allocated to one of the arms. The randomization will be stratified by centre.

The Investigators will not be aware of randomization sequence before allocation of the study group. Every patient's assignment will occur at the time of admission to the clinical department by the Investigators and antibiotics will be given according to the protocol.

5.2 Treatment Compliance

Compliance will be assessed on the basis of capsule counts/medical record.

Policlinico, Via del Pozzo, 71 41100 Modena
Tel. +39-059-4222198-4224198 Fax. +39-059-4224231
e-mail: fabbri.leonardo@unimo.it
www.pneumologiamo.it

5.3 Concomitant Medication

Starting at admission each patient will also receive inhaled bronchodilators and either oral or intravenous glucocorticosteroids for 14 days according to guidelines (1). These 14 days of gluococorticosteroids will include any days of glucocorticoids taken by the patient at home before admission. Additional use, in case of need, of short acting bronchodilators will also be allowed and recorded throughout all the study.

Patients can be enrolled even if antibiotic treatment was started before hospital admission.

The selection of antibiotic (amoxycilline clavulanate or quinolones), its way of administration and dose will be decided by the Investigators according to guidelines (1).

The patients will remain hospitalized for at least 3 days afterwards they will be discharged according to guidelines (1). At discharge, all patients will be prescribed regular treatment according to guidelines (1).

Treatment will be monitored and recorded regularly at each visit.

6 CLINICAL PROCEDURES

VISIT 1 – DAY 0 - HOSPITALIZATION

When the patient arrives in the hospital with a diagnosis of COPD exacerbation, he/she will be examined by the investigator. The inclusion/exclusion criteria will be examined and if the patient satisfies these criteria the investigator will obtain written informed consent to participate in the study. The procedures for Visit 1 will follow.

The Visit 1 procedures will include:

- Revision of inclusion/exclusion criteria
- Written informed consent
- medical history
- physical examination
- measurement of vital signs and body temperature
- information on concomitant medication
- blood sampling (10 ml) for:
  - haematological and chemical analysis (local laboratory)
  - pro-calcitonin (central laboratory, University of Padua)
  - C reactive protein (CRP), serum amyloid (SAA), RANTES (central laboratory, University of Padua)
  - PTX3 and IL-1 decoy (central laboratory, University of Milan, Istituto Clinico Humanitas – ANCILLARY PROJECT 2)

Policlinico, Via del Pozzo, 71 41100 Modena
Tel. +39-059-4222198-4224198 Fax. +39-059-4224231
e-mail: fabbri.leonardo@unimo.it
www.pneumologiamo.it

- chest radiography
- respiratory symptoms using previously validated questionnaires (24-26)
- Pharmacoeconomy questionnaire
- spirometry performed according to guidelines (22)
- sputum collection: 1.5 ml of sputum produced by the patient will be obtained. ½ of this sample will be sent to the local laboratory for bacteriology and intracellular pathogens examination. Each center will explicitly ask its local laboratory to recognize 10^{10} CFU/ml. ¼ of the sputum sample will be stored at -80°C and sent to the University of Ferrara for respiratory viruses RT-PCR analysis (ANCILLARY PROJECT 3). The final ¼ of the sample will be used for inflammatory cell counts, slide preparation and the supernatants will be stored at -80°C for immunoistochemistry. Each center will be responsible for preparing a sputum slide whose reading will be centralized at the University of Ferrara. The presence of blood in the sputum does not invalidate the sample.
- arterial blood gas analysis
- ECG
- Treatment compliance monitoring
- Adverse events monitoring (after antibiotic treatment administration)

VISIT 2 – DAY 1

The Visit 2 procedures will include:

- physical examination
- measurement of vital signs and body temperature
- information on concomitant medication
- blood sampling (3 ml) for:
  - pro-calcitonin (central laboratory, University of Padua)
  - CRP, SAA, RANTES (central laboratory, University of Padua)
- arterial blood gas analysis
- treatment compliance monitoring
- adverse events monitoring (including any episodes of pulmonary tromboembolism, cardiovascular events including acute onset pulmonary edema, and pneumonia)

VISIT 3 – DAY 2

The Visit 3 procedures will include:

Policlinico, Via del Pozzo, 71 41100 Modena
Tel. +39-059-4222198-4224198 Fax. +39-059-4224231
e-mail: fabbri.leonardo@unimo.it
www.pneumologiamo.it

- physical examination
- measurement of vital signs and body temperature
- information on concomitant medication
- blood sampling (5 ml) for:
  - haematological and chemical analysis (local laboratory), only those parameters (if any) which were abnormal at admission
  - pro-calcitonin (central laboratory, University of Padua). At the end of day 2 patients will be randomized to one of the two treatment arms. The 3 pro-CT samples (day 0, 1, 2) of the patients randomized to the pro-CT arm will be sent by courier to the central laboratory of Padua on day 2. The central lab will return the pro-CT dosage to the center the following day (day 3) by noon.
  - CRP, SAA, RANTES (central laboratory, University of Padua)
- arterial blood gas analysis
- treatment compliance monitoring
- adverse events monitoring (including any episodes of pulmonary tromboembolism, cardiovascular events including acute onset pulmonary edema, and pneumonia)

VISIT 4 – AT DISCHARGE AND/OR AT DAY 10

The Visit 4 procedures will include:

- physical examination
- measurement of vital signs and body temperature
- information on concomitant medication
- blood sampling (10 ml) for:
  - haematological and chemical analysis (local laboratory)
  - pro-calcitonin (central laboratory, University of Padua)
  - CRP, SAA, RANTES (central laboratory, University of Padua)
  - PTX3 and IL-1 decoy (central laboratory, University of Milan, Istituto Clinico Humanitas – ANCILLARY PROJECT 2)
- respiratory symptoms using previously validated questionnaires (24-26)
- Pharmacoeconomy questionnaire
- spirometry performed according to guidelines (22)
- sputum collection: 1.5 ml of spontaneous or induced sputum will be obtained. ½ of this sample will be sent to the local laboratory for bacteriology and intracellular pathogens

Policlinico, Via del Pozzo, 71 41100 Modena
Tel. +39-059-4222198-4224198 Fax. +39-059-4224231
e-mail: fabbri.leonardo@unimo.it
www.pneumologiamo.it

examination. Each center will explicitly ask its local laboratory to recognize 10^{10} CFU/ml. ¼ of the sputum sample will be stored at -80°C and sent to the University of Ferrara for respiratory viruses RT-PCR analysis (ANCILLARY PROJECT 3). The final ¼ of the sample will be used for inflammatory cell counts, slide preparation and the supernatants will be stored at -80°C for immunoistochemistry. Each center will be responsible for preparing a sputum slide whose reading will be centralized at the University of Ferrara. The presence of blood in the sputum does not invalidate the sample.

- arterial blood gas analysis
- Treatment compliance monitoring
- Adverse events monitoring (including any episodes of pulmonary tromboembolism, cardiovascular events including acute onset pulmonary edema, and pneumonia)

VISIT 5 – DAY 30

The Visit 5 procedures will include:

- physical examination
- measurement of vital signs
- information on concomitant medication
- blood sampling (3 ml) for:
  - pro-calcitonin (central laboratory, University of Padua)
  - CRP, SAA, RANTES (central laboratory, University of Padua)
- respiratory symptoms using previously validated questionnaires (24-26)
- Pharmacoeconomy questionnaire
- spirometry performed according to guidelines (22)
- sputum collection: 1.5 ml of spontaneous or induced sputum will be obtained. ½ of this sample will be sent to the local laboratory for bacteriology and intracellular pathogens examination. Each center will explicitly ask its local laboratory to recognize 10^{10} CFU/ml. ¼ of the sputum sample will be stored at -80°C and sent to the University of Ferrara for respiratory viruses RT-PCR analysis (ANCILLARY PROJECT 3). The final ¼ of the sample will be used for inflammatory cell counts, slide preparation and the supernatants will be stored at -80°C for immunoistochemistry. Each center will be responsible for preparing a sputum slide whose reading will be centralized at the University of Ferrara. The presence of blood in the sputum does not invalidate the sample.
- arterial blood gas analysis
- Adverse events (including any exacerbation of COPD since the last visit, any hospitalization and/or admission to an Intensive Care Unit, any episodes of pulmonary tromboembolism,

cardiovascular events including acute onset pulmonary edema, pneumonia occurred since the last visit)

VISIT 6 – AT DAY 90

The Visit 6 procedures will include:

- physical examination
- measurement of vital signs
- information on concomitant medication
- blood sampling (10 ml) for:
  - haematological and chemical analysis (local laboratory)
  - pro-calcitonin (central laboratory, University of Padua)
  - CRP, SAA, RANTES (central laboratory, University of Padua)
  - PTX3 and IL-1 decoy (central laboratory, University of Milan, Istituto Clinico Humanitas – ANCILLARY PROJECT 2)
- respiratory symptoms using previously validated questionnaires (24-26)
- Pharmacoeconomy questionnaire
- spirometry performed according to guidelines (22)
- arterial blood gas analysis
- Adverse events (including any exacerbation of COPD since the last visit, any hospitalization and/or admission to an Intensive Care Unit, any episodes of pulmonary tromboembolism, cardiovascular events including acute onset pulmonary edema, pneumonia occurred since the last visit)

VISIT 7 – AT DAY 180

The Visit 7 procedures will include:

- physical examination
- measurement of vital signs
- information on concomitant medication
- blood sampling (3 ml) for:
  - pro-calcitonin (central laboratory, University of Padua)
  - CRP, SAA, RANTES (central laboratory, University of Padua)
- respiratory symptoms using previously validated questionnaires (24-26)

Policlinico, Via del Pozzo, 71 41100 Modena
Tel. +39-059-4222198-4224198 Fax. +39-059-4224231
e-mail: fabbri.leonardo@unimo.it
www.pneumologiamo.it

- Pharmacoeconomy questionnaire
- spirometry performed according to guidelines (22)
- sputum collection: 1.5 ml of spontaneous or induced sputum will be obtained. ½ of this sample will be sent to the local laboratory for bacteriology and intracellular pathogens examination. Each center will explicitly ask its local laboratory to recognize 10^{10} CFU/ml. ¼ of the sputum sample will be stored at -80°C and sent to the University of Ferrara for respiratory viruses RT-PCR analysis (ANCILLARY PROJECT 3). The final ¼ of the sample will be used for inflammatory cell counts, slide preparation and the supernatants will be stored at -80°C for immunoistochemistry. Each center will be responsible for preparing a sputum slide whose reading will be centralized at the University of Ferrara. The presence of blood in the sputum does not invalidate the sample.
- arterial blood gas analysis
- Adverse events (including any exacerbation of COPD since the last visit, any hospitalization and/or admission to an Intensive Care Unit, any episodes of pulmonary tromboembolism, cardiovascular events including acute onset pulmonary edema, pneumonia occurred since the last visit)

In addition to the above procedures, all patients will be contacted by telephone at months 2, 4 and 5 to obtain information on clinical status, treatment monitoring, adverse events monitoring and pharmacoeconomy.

6.1 Central Laboratory Procedures

All procedures will be detailed in the Study Procedures Handbook, which will be distributed to all Investigators before the beginning of the study.

7 ENDPOINTS AND SAFETY EVALUATIONS

7.1 Study endpoints

The primary endpoint of the study will be the rate of exacerbation at 6 months.

Secondary endpoints will be:

- Hospital readmission
- Admission to ICU (Intensive Care Unit)
- Change in FEV1
- Length of the Hospital stay

Policlinico, Via del Pozzo, 71 41100 Modena
Tel. +39-059-4222198-4224198 Fax. +39-059-4224231
e-mail: fabbri.leonardo@unimo.it
www.pneumologiamo.it

- Number of deaths from any cause during follow up

7.2 Safety Evaluation / Adverse Event

The Investigator will evaluate the patients for any possible adverse event at each visit, using a standard clinical criteria in particular for pneumonia, thromboembolism, acute pulmonary edema and other cardiovascular events. The Investigator will also question the patients about other possible adverse event.

8 STATISTICAL METHODOLOGY

8.1 Sample size

The sample size has been estimated according to the primary outcome of the study, i.e. the exacerbation rate at 6 months calculated by dividing the number of patients experiencing at least one exacerbation over a period of 6 months by the total number of patient initially randomized.

The rate of COPD exacerbation/treatment failure has been chosen as primary endpoint because it is clinically relevant for the patients and because this is where differences between the 2 groups, if any, may occur.

To estimate the frequency of this primary outcome data from a previous study have been used. These data suggest that exacerbation occur in about 50% of patient within 6 months (10).

To define non-inferiority with regard to the primary outcome, we settled on a 15% difference in the percentage of patients with an exacerbation within 6 months of follow up as clinically tolerable upper limit. We hypothesized that the exacerbation rates for both treatment plans are equal (at 50%), and that a difference of 15% or less would be unimportant, setting β=0.20 and α=0.05 (one tail) the estimated simple size is 140 patients per arm.

Formally, the null hypothesis is that the event rate for the standard treatment plan is 15% lower than the pro-ct guided plan, and the study has a power of 80% to reject this null hypothesis.

Similarly, the likelihood is 80% than the 95% confidence interval for the difference in event rates will exclude a 15% difference in favor of the standard treatment.

Taking into account an estimated 30% drop-out of patients at 6 months follow up, we increased the sample size up to 200 patient per arm.

Sample size has been estimated by using power and precision, which is a computer program for statistical power analysis and confidence intervals.

8.2 Data Analysis

For each single patient, all study data obtained by interview, clinical and laboratory tests and reviewing of the medical records will be reported in a Case Report Form (CRF) that will be collected at the end of study by the external Clinical Research Organization (CROM Srl, Verona)

Policlinico, Via del Pozzo, 71 41100 Modena
Tel. +39-059-4222198-4224198 Fax. +39-059-4224231
e-mail: fabbri.leonardo@unimo.it
www.pneumologiamo.it

and sent to the Clinical Research Office of the Department of Modena where it will be entered into a database. The use of this database will be restricted to scientific purposes.

A data and safety monitoring board, consisting of a multidisciplinary panel chaired by Dr. Roberto D'Amico will review the database regularly and perform monitoring functions offering recommendations to the Safety and Efficacy Data Monitoring Committee about study continuation.

The study will be terminated prematurely if a disadvantage become evident in one treatment arm assessed. Because in non-inferiority trials intention to treat and per protocol analyses may both falsely favour the conclusion (6), we will analyze our data in both ways. Means, standard deviation, median, proportion, hazard rate and statistical test for significance will be calculated, along with 95% confidence interval. The t test will be used for continuous variables, whereas the chi square test will be used for discrete variables. The association between treatment and outcomes will be estimated by calculating odd ratios. Logistic regression models will be fitted to the data to adjust for potential unbalances of prognostic factors. In addition, COX's regression models will be fitted at 6 months to account for the precise event times and patients lost to follow up. In order to avoid potential bias due to differences in subgroup characteristics, we will randomize patients within the pre-specified strata at the time of study entry.

This study will be guided by a Steering Committee consisting of clinical experts in COPD, infectious diseases, and clinical pharmacology (L. Fabbri, Chair, A. Papi, L. Richeldi, R. D'Amico, B. Muller) and monitored by an independent Safety Committee (P. Geppetti, Chair, C. Giuntini, S. Johnston) that will review the results of one interim analysis of the primary efficacy endpoint and cumulative data on serious adverse events.

An external professional Clinical Research Organization (CROM Srl, Verona) will be responsible for the training of the personnel of the individual centers, regular monitoring (7 visits throughout the study) and quality control of the procedures and data collection.

8.3 Safety analysis

The assessment of safety will be based mainly on the frequency of adverse events and on the number of laboratory values that fall outside of pre-determined ranges. Other safety data (e.g. electrocardiogram, vital signs, special tests) will be considered as appropriate.

Adverse events will be listed summarizing the number and percentage of patients experiencing adverse event. Any other information collected (e.g. severity or relationship to the study medication) will be listed as appropriate.

Laboratory data will be presented by summary statistics of raw data and change from baseline values (means, medians, standard deviations, ranges).

Policlinico, Via del Pozzo, 71 41100 Modena
Tel. +39-059-4222198-4224198 Fax. +39-059-4224231
e-mail: fabbri.leonardo@unimo.it
www.pneumologiamo.it

UNIVERSITÀ DEGLI STUDI DI MODENA E REGGIO EMILIA

SERVIZIO SANITARIO REGIONALE EMILIA-ROMAGNA Azienda Ospedaliero-Universitaria di Modena

9 DIRECT ACCESS TO SOURCE DATA

Crom Srl is asked to verify that the data required by the protocol are accurately reported on the CRF, by direct reference to the source documents, which may include original recordings, laboratory reports, patient notes, etc.

The patient must have consented to their medical records being viewed by authorized personnel and by regulatory authorities. This information is included in the informed consent.

10 ETHICS

The study will be submitted for approval to the local ethic committee for human studies at each institution and a written informed consent will be obtained from all participants. The technology required to perform the study is available in all Centers as part of routine daily clinical work.

11 STUDY MONITORING

During the course of the study, Crom assigned Clinical Monitor will conduct regular site visits to the investigational facilities for the purpose of monitoring various aspects of the study.

12 REFERENCES

1. Global Initiative for Chronic Obstructive Lung Disease. Update 1 July 2005. A collaborative project of the National Health L, and Blood Institute, NIH and the World Health Organisation., ed. www.goldcopd.com: National Institutes of Health, National Heart Lund and Blood Institute, Bethesda, Md, 2005. 2005.
2. Celli BR, MacNee W. Standards for the diagnosis and treatment of patients with COPD: a summary of the ATS/ERS position paper. Eur Respir J 2004;23:932-46.
3. Chapman KR, Mannino DM, Soriano JB, et al. Epidemiology and costs of chronic obstructive pulmonary disease. Eur Respir J 2006;27:188-207.
4. Papi A, Luppi F, Franco F, et al. Pathophysiology of exacerbations of chronic obstructive pulmonary disease. Proc Am Thorac Soc. 2006; In press.
5. ISTAT. http://www.istat.it/dati/dataset/20051107_00/
6. Ministero della Salute.http://www.ministerosalute.it/programmazione/sdo/ric_informazioni/default.jsp
7. Lucioni C, Donner C, De Benedetto F, et al. I costi della broncopneumopatia cronica ostruttiva: la fase prospettica dello Studio ICE (Italian Costs for Exacerbations in COPD). PharmacoEconomics 2005;7:119-134.
8. Seemungal TA, Wedzicha JA. Viral infections in obstructive airway diseases. Curr Opin Pulm Med 2003;9:111-6.
9. Anthonisen NR, Manfreda J, Warren CP et al. Antibiotic therapy in exacerbations of chronic obstructive pulmonary disease. Ann Intern Med. 1987; 106:196-204.

Policlinico, Via del Pozzo, 71 41100 Modena
Tel. +39-059-4222198-4224198 Fax. +39-059-4224231
e-mail: fabbri.leonardo@unimo.it
www.pneumologiamo.it

10. Stolz D, Christ-Crain M, Bingisser R, Leuppi J, Miedinger D, Mueller C, Huber P, Muller B, Tamm, M. Procalcitonin for guidance of antibiotic therapy in acute exacerbations of COPD - A Randomised trial. 2006. Am J Respir Cr Care Med, 2006, submitted.
11. Woodhead M, Blasi F, Ewig S, et al. Guidelines for the management of adult lower respiratory tract infections. Eur Respir J 2005;26:1138-80.
12. Saint S, Bent S, Vittinghoff E, et al. Antibiotics in chronic obstructive pulmonary disease exacerbations. A meta-analysis. Jama 1995;273:957-60.
13. Nouira S, Marghli S, Belghith M, et al. Once daily oral ofloxacin in chronic obstructive pulmonary disease exacerbation requiring mechanical ventilation: a randomised placebo-controlled trial. Lancet 2001;358:2020-5.
14. Papi A, Bellettato C, Braccioni F, et al. Infections and airway inflammation in chronic obstructive pulmonary disease severe exacerbations. Am J Respir Crit Care Med 2006; submitted and provisionally accepted.
15. Seemungal T, Harper-Owen R, Bhowmik A, et al. Respiratory viruses, symptoms, and inflammatory markers in acute exacerbations and stable chronic obstructive pulmonary disease. Am J Respir Crit Care Med 2001;164:1618-23.
16. Christ-Crain M, Jaccard-Stolz D, Bingisser R, et al. Effect of procalcitonin-guided treatment on antibiotic use and outcome in lower respiratory tract infections: cluster-randomised, single-blinded intervention trial. Lancet 2004;363:600-7.
17. Christ-Carin M, Muller B. Procalcitonin in bacterial infections--hype, hope, more or less? Swiss Med Wkly 2005;135:451-60.
18. Muller B, Peri G, Doni A, et al. High circulating levels of the IL-1 type II decoy receptor in critically ill patients with sepsis: association of high decoy receptor levels with glucocorticoid administration. J Leukoc Biol 2002;72:643-9.
19. Simon L, Gauvin F, Amre DK, et al. Serum procalcitonin and C-reactive protein levels as markers of bacterial infection: a systematic review and meta-analysis. Clin Infect Dis 2004; 39:206-17.
20. Hurst JR, Wedzicha JA. Chronic obstructive pulmonary disease: the clinical management of an acute exacerbation. Postgrad Med J 2004;80:497-505.
21. Soto FJ, Varkey B. Evidence-based approach to acute exacerbations of COPD. Curr Opin Pulm Med 2003;9:117-24.
22. Sterk PJ, Fabbri LM, Quanjer PH, et al. Airway responsiveness. Standardized challenge testing with pharmacological, physical and sensitizing stimuli in adults. Report Working Party Standardization of Lung Function Tests, European Community for Steel and Coal. Official Statement of the European Respiratory Society. Eur Respir J Suppl 1993;16:53-83.
23. Pocock SJ. The pros and cons of noninferiority trials. Fundam Clin Pharmacol 2003;17:483-90.
24. Brazier JE, Harper R, Jones NM, O'Cathain A, Thomas KJ, Usherwood T, Westlake L. Validating the SF-36 health survey questionnaire: new outcome measure for primary care. BMJ 1992; 305:160-4.
25. Baiardini I, Braido F, Fassio O, Tarantini F, Pasquali M, Tarchino F, Berlendis A, Canonica GW. A new tool to assess and monitor the burden of chronic cough on quality of life: Chronic Cough Impact Questionnaire. Allergy 2005;60:482-8.

Policlinico, Via del Pozzo, 71 41100 Modena
Tel. +39-059-4222198-4224198 Fax. +39-059-4224231
e-mail: fabbri.leonardo@unimo.it
www.pneumologiamo.it

26. Braido F, Baiardini I, Tarantini F, Fassio O, Balestracci S, Pasquali M, Tarchino F, Canonica W. Chronic cough and QoL in allergic and respiratory diseases measured by a new specific validated tool-CCIQ. J Investig Allergol Clin Immunol 2006;16:110-116.

Policlinico, Via del Pozzo, 71 41100 Modena
Tel. +39-059-4222198-4224198 Fax. +39-059-4224231
e-mail: fabbri.leonardo@unimo.it
www.pneumologiamo.it

ANCILLARY PROJECTS

Ancillary 1)

Cost-effectiveness analysis of hospitalised COPD exacerbations.

We aim to assess a procalcitonin-based therapeutic strategy to reduce antibiotic use in acute exacerbation of COPD.

The reference for this pharmacoeconomic study is a clinical, comparative, controlled, randomised, openlabel, and multicentric trial (300 patients), comparing routine use of antimicrobial therapy (Group A) with procalcitonin guided antimicrobial treatment (Group B) for acute exacerbation of COPD. We will define each unit of effectiveness as each patient successfully recovered from hospitalised COPD exacerbation. For the analysis, the average cost-effectiveness ratio will be calculated for each alternative in the study that allows to know which therapeutic option is most efficient, i.e., better clinical results with smaller associated costs.

We will consider direct medical costs including direct non-medical costs and indirect costs derived from leaves or deaths. A sensitivity analysis will be performed on the parameters that provide more weight for the results.

References

1. Ministero della Salute. Osservatorio Nazionale sull'Impiego dei Medicinali. L'uso dei farmaci in Italia. Rapporto nazionale anno 2005. Roma: Ministero della Salute 2005.
2. IMS. *Indicatori farmaceutici 2001*. Milano: IMS 2002.
3. IMS. *Indicatori farmaceutici 2001*. Milano: IMS 2003.
4. *Sistema Statistico Nazionale. Indicatori demografici*. Roma: ISTAT dicembre 2001.
5. Drummond MF, O'Brien BJ, Stoddart GL, Torrance GW. Methods for the economic evaluation of health care programmes. 2nd Edition. Oxford: Oxford University Press 1997.
6. OEMF. L'Informatore farmaceutico. Supplemento n. 11. Milano: OEMF 2001
7. IPASVI. Nomenclatore tariffario 2002 prestazioni di assistenza infermieristica. Roma: IPASVI 2002.
8. Ministero della Sanità. Decreto ministeriale n. 150 del 22 Luglio 1996. Prestazioni di assistenza specialistica ambulatoriale erogabili nell'ambito del Servizio sanitario nazionale e relative tariffe. Supplemento ordinario alla "Gazzetta Ufficiale" n. 216 del 14 settembre 1996 - Serie generale. Roma: Istituto Poligrafico e Zecca dello Stato 1996.

Policlinico, Via del Pozzo, 71 41100 Modena
Tel. +39-059-4222198-4224198 Fax. +39-059-4224231
e-mail: fabbri.leonardo@unimo.it
www.pneumologiamo.it

9. Ministero della Sanità. Decreto ministeriale n. 178 del 30 giugno 1997. Aggiornamento delle tariffe delle prestazioni di assistenza ospedaliera, di cui al decreto ministeriale 14 dicembre 1994. Supplemento ordinario alla "Gazzetta Ufficiale" n. 209 dell'8 settembre 1997 - Serie generale. Roma: Istituto Poligrafico e Zecca dello Stato 1997.
10. Ospedale Maggiore di Milano. Rapporto di gestione 1999: 34. Milano: Ospedale Maggiore di Milano 2001.
11. Banca d'Italia. Supplementi al Bollettino Statistico. Note metodologiche e informazioni statistiche. I bilanci delle famiglie italiane nell'anno 2000. Roma: Banca d'Italia 2002;6:46;54.
12. Briggs AH, Gray AM. Handling uncertainty when performing economic evaluation of healthcare interventions. Health Technol Assess 1999;3:1-134.

Ancillary 2)

Inflammatory markers and exacerbation aetiology and severity.

The aim of the project is to identify novel markers of immunity and/or of inflammatory response able to discriminate whether a patients is at risk of infection disease or complication related to infection disease. We will focus our attention on recently identified molecules called long pentraxin (PTX3). An original assay to quantify this molecules has been validated in recent studies (1). PTX3 is structurally related to C-reactive protein that play a non-redudant role in innate immune response against lung pathogens, fungi and bacteria (i.e. Klebsiella pneumoniae, etc). Furthermore, PTX3 plays a regulatory role in innate immune response and in inflammatory cascade. Increased level of PTX3 have been described in patients with infections including pulmonary infections. We will also measure levels of interlukin-1 (IL-1) decoy receptor. IL-1 decoy receptor is essential for the negative regulation of IL-1 system that can be used as marker of endogenous anti-inflammatory pathways (2). PTX3 and IL-1 decoy receptor will be measured first in a subgroup of subjects enrolled in the study (50 subjects) and than the analysis will be extended to all the study population. Serum amyloid A, a protein of the acute-phase inflammatory response, and additional biomarkers (such as C reactive protein and RANTES) will be also measured in plasma samples and relationship with exacerbation aetiology and/or severity analysed. C -reactive protein (CRP) and serum amyloid A (SAA) are members of a family of proteins that are major components of the acute-phase

inflammatory response. They are synthesised in the liver in response to infection, inflammation, injury or stress, and therefore they increase in chronic obstructive pulmonary disease. Serum levels of these proteins are used as markers of disease activity and to monitor the response to therapy thanks to their dynamic range and short half-life. *CRP* and *SAA* are measured by using a specific immunonephelometric methods automatized on nephelometer BNII (Dade Behring, Italy).

References

1) Latini R, Maggioni AP, Peri G, et al. Lipid Assessment Trial Italian Network (LATIN) Investigators. Prognostic significance of the long pentraxin PTX3 in acute myocardial infarction. Circulation. 2004 Oct 19;110(16):2349-54.

2) Muller B, Peri G, Doni A, et al. High circulating levels of the IL-1 type II decoy receptor in critically ill patients with sepsis: association of high decoy receptor levels with glucocorticoid administration. J Leukoc Biol. 2002;72:643-9.

Ancillary 3)

Relationship between COPD exacerbation's etiology and procalcitonin serum levels.

The aim of this project is to validate the rationale for the use of serum procalcitonin to guide the requirement for antibiotic treatment in COPD exacerbations. Given the fact that a great proportion of COPD patients are colonized by bacteria in stable conditions, and since an increased sputum bacterial load is expected in COPD exacerbation associated to bacterial infection, we will analyze the relationship between bacterial load (quantitative bacteriology) and serum procalcitonin levels to evaluate whether a direct relationship is present between the severity of airway infection and serum levels of procalcitonin. We will also analyze serum procalcitonin levels (and changes from stable conditions) in COPD exacerbation not associated to bacterial infection (viral infection and non infective exacerbations). Bacterial isolation and identification will be performed with the use of standard techniques.

Policlinico, Via del Pozzo, 71 41100 Modena
Tel. +39-059-4222198-4224198 Fax. +39-059-4224231
e-mail: fabbri.leonardo@unimo.it
www.pneumologiamo.it

Colony forming units (CFU)/mL will be then calculated. (1). A panel of RT-PCRs will be used to screen sputum samples for respiratory virus and atypical bacteria detection (2).

References

1) Barrow GI. Feltham RK. Cowan and Steel's manual for the identification of medical bacteria, 3rd ed. Cambridge, UK: Cambridge University Press; 1993.
2) Papi A, Bellettato C, Braccioni F, et al. Infections and airway inflammation in chronic obstructive pulmonary disease severe exacerbations. Am J Respir Crit Care Med 2006; In press.

Policlinico, Via del Pozzo, 71 41100 Modena
Tel. +39-059-4222198-4224198 Fax. +39-059-4224231
e-mail: fabbri.leonardo@unimo.it
www.pneumologiamo.it
