# Supplementary material for: Antibiotic Treatment of Severe Exacerbations of Chronic Obstructive Pulmonary Disease with Procalcitonin: A Randomized Noninferiority Trial
Source: PLoS One. 2015 Mar 11;10(3):e0118241. doi: 10.1371/journal.pone.0118241 (PMC4356612; doi:10.1371/journal.pone.0118241)
Supplement: S1 Report — (PDF) [file pone.0118241.s004.pdf]

CONFIDENTIAL

Case Report Form

TITLE

COMPARISON OF A SERUM PROCALCITONIN (PRO-CT) GUIDED TREATMENT PLAN WITH THE STANDARD GUIDELINE RECOMMENDED ANTIBIOTIC TREATMENT PLAN FOR PATIENTS HOSPITALIZED WITH A DIAGNOSIS OF EXACERBATION OF COPD

Centre No.: |___|___|___|

Patient's initials: |___|___| |___|___|___|
Family name Name

Screening No.: |___|___|___|
Chronological patient no.

Randomisation No.: |___|___|___|
Randomisation no.

FINAL VERSION: SEPTEMBER 27, 2006

INSTRUCTIONS

Please complete each form; date and sign the Investigator's Statement at the end of each visit as indicated in a legible manner.
The use of a black ball point pen is recommended.
If a change is necessary to any entry, cross it out with a single line, write in the correction and give reason for the change. Initial and date the correction.
Do not leave any questions unanswered. Please write the explanation beside any blank spaces.

Flow chart

	Hospitalization						
	Visit 1	Visit 2	Visit 3	Visit 4	Visit 5	Visit 6	Visit 7
	Day 0	Day 1	Day 2 Randomisation	At hospital discharge e/o day 10	Day 30	Day 90	Day 180
**Informed Consent**	x						
**Medical history**	x						
**Physical examination**	x	x	x	x	x	x	x
**Vital signs**	x	x	x	x	x	x	x
**Body temperature**	x	x	x	x			
**Medication**	x	x	x	x	x	x	x
**Pulmonary x-ray**	x						
**Spirometry**	x			x	x	x	x
**Sputum collection**	x			x	x		x
**ECG**	x						
**Inclusion / exclusion criteria**	x						
**Questionnaire for quality of life**	x			x	x	x	x
**Pharmaco-economy questionnaire**	x			x	x	x	x
**Haematology**	x		x (if abnormal at day 0)	x		x	
**Blood chemistry**	x		x (if abnormal at day 0)	x		x	
**CRP, SAA and RANTES**	x	x	x	x	x	x	x
**PTX3 and IL-1 decoy**	x			x		x	
**Pro-ct levels**	x	x	x	x	x	x	x
**Arterial blood gases**	x	x	x	x	x	x	x
**Treatment compliance**	x	x	x	x			
**Adverse event**	x	x	x	x	x	x	x

Study Design

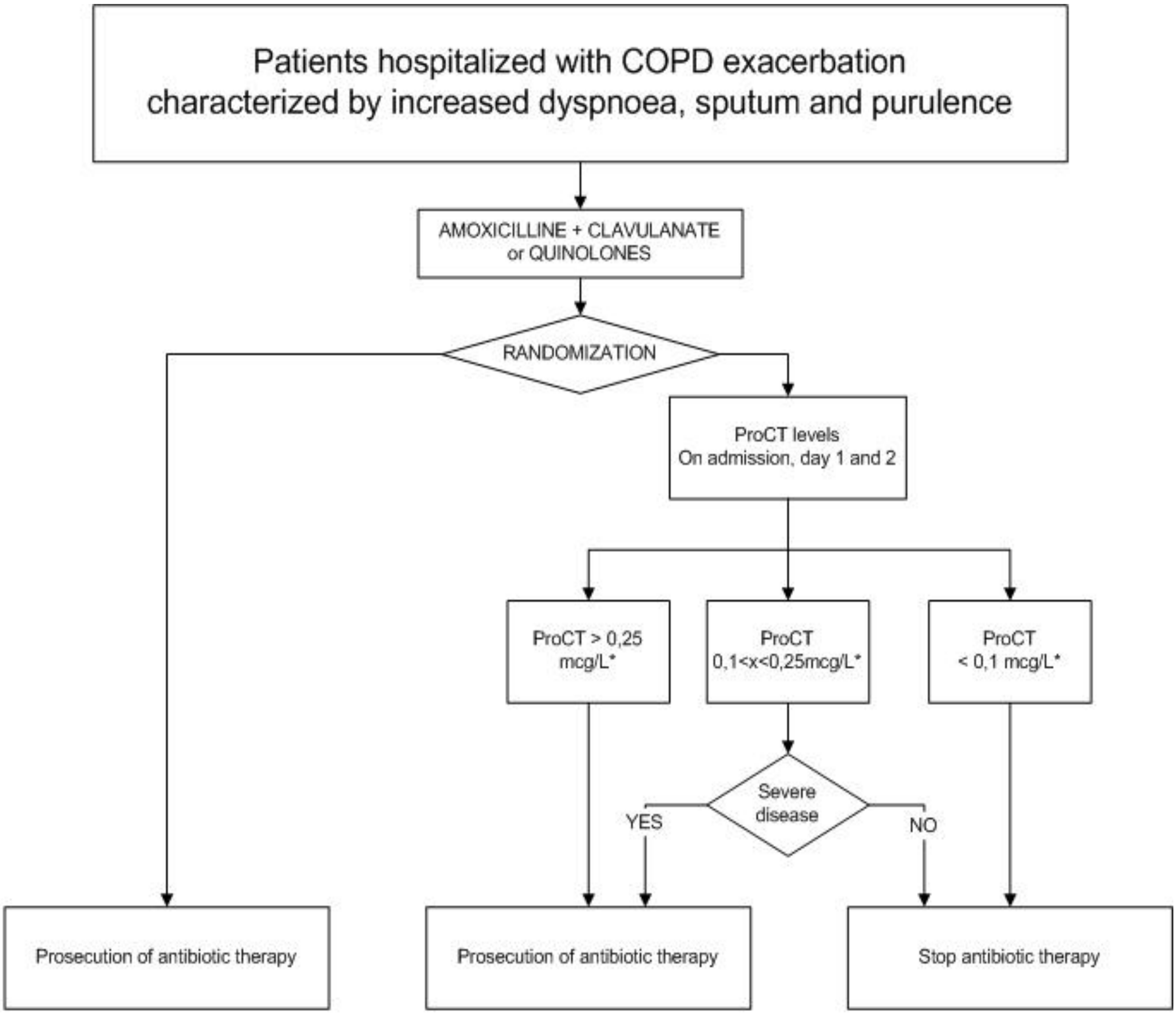

* On admission, day 1 and day 2

VISIT 1 – Day 0

Visit date |___|___||___|___||___|___| at |___|___| |___|___|
D D M M Y Y HOUR MINUTES

Participation agreement attestation:

I the undersigned, Doctor ……………………….., certify that the patient's written informed consent form has been read, understood**,** approved and signed by the patient after having received information.

Date of written informed consent signature: |___|___| |___|___| |___|___|
D D M M Y Y

Investigator's Signature: . . . .

The informed consent form must be signed before any procedure related to the study.

Centre No. |___|___|___| Screen. No. |___|___|___| Randomisation No. |___|___|___| Pat. Init. |___|___| |___|___|___|
Family name Name

VISIT 1 – Day 0

Visit date |___|___||___|___||___|___| at |___|___| |___|___|
D D M M Y Y HOUR MINUTES

INCLUSION CRITERIA	Yes	No
**1.** Male and female patients who give written informed consent	❑	❑*
**2.** Age: adults > 18 years old	❑	❑*
**3.** Diagnosis of COPD according to GOLD guidelines: FEV1/FVC < 70% with FEV1 < 80% of predicted	❑	❑*
**4.** History of cigarette smoking	❑	❑*
**5.** Diagnosis of COPD exacerbation: defined as acute-onset dyspnoea and/or cough associated with purulent sputum production (Anthonisen's criteria); requiring, according to guidelines (GOLD 2005), treatment with antibiotic; requiring hospitalisation	❑	❑*

Any NO* leads to immediate exclusion from the study.

Fill in the **"Investigator's statement"** at the end of visit 1 and the **"Study Conclusion Summary/Wihtdrawal/Drop-out Form"**.

EXCLUSION CRITERIA	Yes	No	NA
**1.** Female subjects: pregnant, lactating mother or lack of efficient contraception in a subject with child-bearing potential (e.g. contraceptive methods other than oral contraceptives, IUD, tubal ligature)	❑*	❑	❑
**2.** Diagnosis of asthma	❑*	❑	
**3.** Coexisting medical conditions: unstable concomitant cardiovascular, renal, hepatic, gastrointestinal, neurological, endocrine, metabolic, musculo-skeletal, neoplastic, respiratory or other clinically significant disease (Patients with stable and well controlled hypertension or diabetes maybe included in the study)	❑*	❑	
**4.** Clinical significant laboratory abnormalities indicating unstable concomitant disease	❑*	❑	
**5.** Patients in whom survival for at least 1 year is unlikely	❑*	❑	
**6.** Inability to give informed consent	❑*	❑	

Any YES* leads to immediate exclusion from the study.

Fill in the **"Investigator's statement"** at the end of visit 1 and the **"Study Conclusion Summary/Wihtdrawal/Drop-out Form"**.

VISIT 1 – Day 0

Visit date |___|___||___|___||___|___| at |___|___| |___|___|
D D M M Y Y HOUR MINUTES

DEMOGRAPHIC DATA

Age |___|___| years old

Date of Birth |___|___||___|___||___|___|
D D M M Y Y

Sex:
1) ❑ Male
2) ❑ Female

Height (cm) |___|___|___|.|___|

Weight (Kg) |___|___|___|.|___|

<u>If the patient is a female</u>

Is she of childbearing potential?
1) ❑ No
2) ❑ Yes

1) If no:

1) Post menopausal (two years after the last period) ❑
2) Surgically sterilised ❑
3) Other: ❑

Specify: ……………………………………………………………….………

2) If yes:

1) Tubal Ligature ❑
2) Using oral oestro-progestatives ❑
3) Using intra uterine-device (IUD) ❑
4) Other: ❑

Specify: ……………………………………………………………….………

Warning: female of childbearing potential must use an effective contraceptive method. If not, the patient cannot be randomised

Centre No. |___|___|___| Screen. No. |___|___|___| Randomisation No. |___|___|___| Pat. Init. |___|___| |___|___|___|
Family name Name

VISIT 1 – Day 0

Visit date |___|___||___|___||___|___| at |___|___| |___|___|
D D M M Y Y HOUR MINUTES

MEDICAL AND SURGICAL HISTORY AND CONCOMITANT DISEASES

Does the patient have a relevant medical and/or surgical history, and/or relevant concomitant diseases? (including clinically significant laboratory values)

❑ No ❑ Yes*

If yes, fill in this table.

**Medical or surgical history and/or concomitant diseases**	**Start Date** DD / MM / YY	**End date** DD / MM / YY	**On going**
1. COPD	|___|___| |___|___| |___|___|	|___|___| |___|___| |___|___|	❑
2.	|___|___| |___|___| |___|___|	|___|___| |___|___| |___|___|	❑
3.	|___|___| |___|___| |___|___|	|___|___| |___|___| |___|___|	❑
4.	|___|___| |___|___| |___|___|	|___|___| |___|___| |___|___|	❑
5.	|___|___| |___|___| |___|___|	|___|___| |___|___| |___|___|	❑
6.	|___|___| |___|___| |___|___|	|___|___| |___|___| |___|___|	❑
7.	|___|___| |___|___| |___|___|	|___|___| |___|___| |___|___|	❑
8.	|___|___| |___|___| |___|___|	|___|___| |___|___| |___|___|	❑
9.	|___|___| |___|___| |___|___|	|___|___| |___|___| |___|___|	❑
10.	|___|___| |___|___| |___|___|	|___|___| |___|___| |___|___|	❑

Centre No. |___|___|___| Screen. No. |___|___|___| Randomisation No. |___|___|___| Pat. Init. |___|___| |___|___|___|
Family name Name

VISIT 1 – Day 0

Visit date |___|___||___|___||___|___| at |___|___| |___|___|
D D M M Y Y HOUR MINUTES

MEDICATION FOR CONDITIONS OTHER THAN COPD

Is the patient under ANY medication for any medical condition other than COPD? ❑ Yes* ❑ No

If YES please complete the following table, specifying ALL medication taken by the patient for ANY medical condition <u>except</u> COPD, at the time of enrolment in the study

**Trade name**	**Indication**	**Posology**		**Route**	**Administration (DD/MM/YY)**	
		Dose/day	Unit		Start date	End date (or ONGOING)

MEDICATION FOR STABLE COPD

Is the patient under pharmacologic treatment for stable COPD? ❑ Yes* ❑ No

If YES please complete the following table, specifying any medication taken by the patient for stable COPD, at the time of enrolment in the study

**Trade name**	**Indication**	**Posology**		**Route**	**Administration (DD/MM/YY)**	
		Dose/day	Unit		Start date	End date (or ONGOING)

Centre No. |___|___|___| Screen. No. |___|___|___| Randomisation No. |___|___|___| Pat. Init. |___|___| |___|___|___|
Family name Name

VISIT 1 – Day 0

Visit date |___|___||___|___||___|___| at |___|___| |___|___|
D D M M Y Y HOUR MINUTES

HOME MEDICATION FOR THE CURRENT COPD EXACERBATION

Did the patient take ANY medication for the current COPD exacerbation <u>before</u> hospital admission *(for example, any antibiotics taken at home before hospital admission)*? ❑ Yes***** ❑ No

****If YES please complete the following table, specifying any medication taken by the patient for the current COPD exacerbation at home (before hospital admission)***

**Trade name**	**Indication**	**Posology**		**Route**	**Administration (DD/MM/YY)**	
		Dose/day	Unit		Start date	End date (or ONGOING)

HISTORY OF CURRENT COPD EXACERBATION

Please indicate when the first sign/symptom of the current COPD exacerbation began (DD/MM/YY):

|___|___| |___|___| |___|___|

What were the characteristics of the current COPD exacerbation? Please indicate:

**Sign/symptom**		**Comments:**
1. Fever	❑ Yes<br>❑ No	
2. Dyspnoea	❑ Yes<br>❑ No	
3. Cough	❑ Yes<br>❑ No	
4. Sputum	❑ Yes<br>❑ No	
5. Sputum purulence	❑ Yes<br>❑ No	
6. Wheezing	❑ Yes<br>❑ No	
7. Other (specify)	❑ Yes<br>❑ No	

VISIT 1 – Day 0

Visit date |___|___||___|___||___|___| at |___|___| |___|___|
D D M M Y Y HOUR MINUTES

PHYSICAL EXAMINATION

Physical examination	Status	Comments: if "abnormal" specify:
Cardiovascular system	❑ Abnormal<br>❑ Normal<br>❑ Not done	
Respiratory system (except COPD)	❑ Abnormal<br>❑ Normal<br>❑ Not done	
Lymphatic system	❑ Abnormal<br>❑ Normal<br>❑ Not done	
Neurological-locomotor system	❑ Abnormal<br>❑ Normal<br>❑ Not done	
Skin	❑ Abnormal<br>❑ Normal<br>❑ Not done	
Endocrine	❑ Abnormal<br>❑ Normal<br>❑ Not done	
Ear-nose-throat system	❑ Abnormal<br>❑ Normal<br>❑ Not done	
Ophtalmological system	❑ Abnormal<br>❑ Normal<br>❑ Not done	
Gastrointestinal system	❑ Abnormal<br>❑ Normal<br>❑ Not done	
Others ……………………..	❑ Abnormal<br>❑ Normal<br>❑ Not done	

If an abnormality is clinically significant, please complete the 'Medical and surgical history and Concomitant Diseases' form on page 4.

VISIT 1 – Day 0

Visit date |___|___||___|___||___|___| at |___|___| |___|___|
D D M M Y Y HOUR MINUTES

SMOKING HABITS

Information on the patient:

Never smoked ❑

Former-smoker ❑ Start date |__|__| |__|__| |__|__| Stop date |__|__| |__|__| |__|__|

Smoker ❑ Start date |__|__| |__|__| |__|__|

For former-smokers and smokers: Number packs year |__|__|

☞ **Reminder**: 1 pack year = 20 cigarettes/day for 1 year or equivalent.
Calculation: PACK-YEARS = $\frac{\text{(number of cigarettes smoked per day)} \times \text{(years smoked)}}{20}$

VITAL SIGNS

Body temperature |___|___|.|___|°C

**SITTING BLOOD PRESSURE** (mmHg)		**SITTING HEART RATE** (bpm)
**Systolic**	**Diastolic**	
|___|___|___|	|___|___|___|	|___|___|___|

Centre No. |___|___|___| Screen. No. |___|___|___| Randomisation No. |___|___|___| Pat. Init. |___|___| |___|___|___|
Family name Name

VISIT 1 – Day 0

Visit date |___|___||___|___||___|___| at |___|___| |___|___|
D D M M Y Y HOUR MINUTES

LABORATORY TESTS

Collect blood sample for haematology and blood chemistry

(LOCAL LABORATORY)

Haematology tests * — **Clinically Significant **** (**Yes** / **No**)

Haemoglobin (mmol/L) |___|___|.|___| Yes |___| No |___|

Haematocrit (L/L) |___|.|___| Yes |___| No |___|

RBC (10^{12}/L) |___|.|___| Yes |___| No |___|

WBC (10^{9}/L) |___|___|.|___| Yes |___| No |___|

WBC (differential counts) Neutrophils (%) |___|___|.|___| Yes |___| No |___|

Eosinophils (%) |___|.|___| Yes |___| No |___|

Basophils (%) |___|.|___| Yes |___| No |___|

Monocytes (%) |___|___|.|___| Yes |___| No |___|

Lymphocytes (%) |___|___|.|___| Yes |___| No |___|

Platelet (10^{9}/L) |___|___|___| Yes |___| No |___|

Blood Chemistry tests *

Alkaline Phosphatase (U/L) |___|___|___|.|___| Yes |___| No |___|

AST (SGOT) (U/L) |___|___|.|___| Yes |___| No |___|

ALT (SGPT) (U/L) |___|___|.|___| Yes |___| No |___|

BUN/blood urea (mmol/L) |___|.|___| Yes |___| No |___|

Creatinine Kinase (U/L) |___|___|___|.|___| Yes |___| No |___|

Creatinine (µmol/L) |___|___|___|.|___| Yes |___| No |___|

Gamma-GT (U/L) |___|___|.|___| Yes |___| No |___|

Sodium (mmol/L) |___|___|___|.|___| Yes |___| No |___|

Potassium (mmol/L) |___|.|___| Yes |___| No |___|

Glucose (mmol/L) |___|.|___|___| Yes |___| No |___|

* to be filled in as soon as results are available

** For the out of range values, please specify if the abnormality is clinically or not clinically significant

Laboratory results need to be stored in the patient's file

Centre No. |___|___|___| Screen. No. |___|___|___| Randomisation No. |___|___|___| Pat. Init. |___|___| |___|___|___|
Family name Name

VISIT 1 – Day 0

Visit date |___|___||___|___||___|___| at |___|___| |___|___|
D D M M Y Y HOUR MINUTES

Pro-calcitonin (pro-ct)

Has the blood sample been taken for the pro-ct analysis?

Yes ❑ No ❑

If Yes, refer to the Study Procedures Handbook for handling and storage.

If No, please specify why: ____

Please fill in as soon as the results are available:

Pro-calcitonin (mcg/L) I___I___I.I___I___I

C-reactive protein (CRP), serum amyloid (SAA) and RANTES

Has the blood sample been taken for the CRP, SAA and RANTES analysis?

Yes ❑ No ❑

If Yes, refer to the Study Procedures Handbook for handling and storage.

If No, please specify why: ____

Please fill in as soon as the results are available:

CRP (mg/L) I___I___I.I___I___I

SAA (mg/L) I___I___I.I___I___I

RANTES (mcg/L) I___I___I.I___I___I

PTX-3 and IL1 decoy

Has the blood sample been taken for the PTX-3 and IL1 decoy analysis?

Yes ❑ No ❑

If Yes, refer to the Study Procedures Handbook for handling and storage.

If No, please specify why: ____

Please fill in as soon as the results are available:

PTX-3 (ng/ml) I___II___I___I.I___I___I

IL1 decoy (ng/ml) I___II___I___I.I___I___I

Centre No. |___|___|___| Screen. No. |___|___|___| Randomisation No. |___|___|___| Pat. Init. |___|___| |___|___|___|
Family name Name

VISIT 1 – Day 0

Visit date |___|___||___|___||___|___| at |___|___| |___|___|
D D M M Y Y HOUR MINUTES

PULMONARY X-RAY

Has a pulmonary X-ray been obtained? Yes ❑ No ❑

If YES: X ray was: Normal ❑ Abnormal ❑

If "Abnormal", please specify:

____

____

If not obtained, specify why:______________________________

QUESTIONNAIRES FOR RESPIRATORY SYMPTOMS/QUALITY OF LIFE

SF36 score: total |___|___|___|

BDI score: total |___|___|___|

TDI score: total |___|___|___|

CCIQ Score: total |___|___|___|

PHARMACOECONOMIC QUESTIONNAIRE

Please remind to fill in the pharmacoeconomic questionnaire and enclose it in the plastic envelope.

SPIROMETRY

- **SPIROMETRY:** from 3 acceptable spirometric manoeuvres record the highest FEV_1 and FVC, irrespective of the manoeuvre (curve) they come from.

date |___|___| |___|___| |___|___| at |__|__| |__|__|
HOUR MINUTES

**FVC**	|___|, |___|___|___| litre
**FEV_1**	|___|, |___|___|___| litre
**FEV_1 predicted normal value**	|___|, |___|___|___| litre [ERS standards]
**FEV_1 (% predicted normal value)**	|___|___| % of predicted value
**FEV_1/FVC (%)**	|___|___|___|, |___| %

VISIT 1 – Day 0

Visit date |___|___||___|___||___|___| at |___|___| |___|___|
D D M M Y Y HOUR MINUTES

MICROBIOLOGY

	Yes	No
Sputum collection performed:	❑	❑

If YES, specify volume of sputum collected: |___|,|___| ml

If NO, specify why ____

Results from local laboratory:

Bacteriology:

Identified microorganism #1: ____________________________ CFU/ml: |___|___|$^{|\_\_|\_\_|}$

Identified microorganism #2: ____________________________ CFU/ml: |___|___|$^{|\_\_|\_\_|}$

Identified microorganism #3: ____________________________ CFU/ml: |___|___|$^{|\_\_|\_\_|}$

Serology for Intracellular pathogens:

Has the sample for serology for intracellular pathogens been obtained? Yes ❑ No ❑

If Yes, please complete below:

Was serology positive for **Mycoplasma pneumoniae**? Yes ❑ No ❑

If YES, indicate: IgM: |___|___|,|___|___|
IgG: |___|___|,|___|___|

Was serology positive for **Chlamydia**? Yes ❑ No ❑

If YES, indicate: IgM: |___|___|,|___|___|
IgG: |___|___|,|___|___|

Was serology positive for **Legionella pneumophila**? Yes ❑ No ❑

If YES, indicate:

	POSITIVE	NEGATIVE
Legionella pneumophila IgM	❑	❑
Legionella pneumophila IgG	❑	❑

VISIT 1 – Day 0

Visit date |___|___||___|___||___|___| at |___|___| |___|___|
D D M M Y Y HOUR MINUTES

MICROBIOLOGY (continued)

Has the sample for **respiratory viruses RT-PCR analysis** been collected and stored at –80°C?

Yes ❑ No ❑

If **YES,** specify volume stored at -80°C for RT-PCR: |___|,|___||___||___| ml

Please remember to label the sample with the patient's ID number and store it at -80°C, as described in the Study Procedures Handbook.

Has the sample for **inflammatory cell counts** been collected? Yes ❑ No ❑

Were the slides prepared and stored at -20°C? Yes ❑ No ❑

If YES: how many slides were prepared? |___|___|

ARTERIAL BLOOD GASES

	**Yes**	**No**
Arterial blood gas analysis performed:	❑	❑
**If YES**: patient was under O_2 therapy?	❑	❑

If patient was under O_2 therapy please indicate:

- how many L/min: |___|,|___|
- how many hours/day: |___|___|

If arterial blood gas analysis was performed, indicate:

pH: |___|,|___|___|

PaO_2: |___|___|___| mmHg

$PaCO_2$: |___|___|___| mmHg

SO_2: |___|___|___| %

HCO_3^-: |___|___|___| mmol/L

VISIT 1 – Day 0

Visit date |___|___||___|___||___|___| at |___|___| |___|___|
D D M M Y Y HOUR MINUTES

ECG (12-lead ECG)

Has an ECG recording been obtained? Yes ❑ No ❑
If No specify why: ____
If YES, please complete the following table and file the original ECG in the patient's file

RECORDING	***COMMENTS (if any)***
Normal ❑	___________________________________
Abnormal (not clinically significant) ❑	___________________________________
Abnormal (clinically significant) ❑	___________________________________
(In case of clinically significant abnormalities, complete the 'Medical and surgical history and Concomitant Diseases' form on page 4)	___________________________________

ANTIBIOTIC TREATMENT ADMINISTRATION

- Indicate the antibiotic prescribed to the patient during the current hospitalization (choose one):

Amoxicilline + Clavulanate ❑ OR **Quinolones** ❑ (specify kind _________________)

- Indicate route of administration (choose one): per os ❑ intravenous ❑
- record dosage of administered antibiotic: _______________________________________
- Indicate start date of antibiotic (DD/MM/YY): |___|___||___|___||___|___|

HOSPITAL MEDICATION

Please record in the following table ALL medication (steroid treatment, bronchodilators, any other therapy prescribed) to the patient during the current hospitalization (except antibiotic treatment, since it was already indicated above)

**Trade name**	**Indication**	**Posology**		**Route**	**Administration (DD/MM/YY)**	
		Dose/day	Unit		Start date	End date (or ONGOING)

VISIT 1 – Day 0

Visit date |___|___||___|___||___|___| at |___|___| |___|___|
D D M M Y Y HOUR MINUTES

ADVERSE EVENTS related to antibiotic treatment

Did any adverse event related to antibiotic treatment occur on day 0?

❑ Yes
❑ No

If Yes, please complete the appropriate "adverse event" page and report it immediately to the Direzione Sanitaria of the Hospital and to CROM (see Study Procedures Handbook for instructions).

COMPLIANCE

	Yes	No
Is patient compliant:	❑	❑

If "No", please specify:

____

____

INVESTIGATOR'S STATEMENT

I confirm that the data herein is a true complete and accurate reflection of this subject participation in the study, according to the protocol procedures.

Investigator's name: ……………………………………………………………………………

Investigator's signature: ……………………………………………………………………………

Date: |___|___||___|___||___|___|
D D M M Y Y

VISIT 2 – Day 1

Visit date |___|___||___|___||___|___| at |___|___| |___|___|
D D M M Y Y HOUR MINUTES

Has the patient withdrawn prematurely from the study?

❑ Yes
❑ No

If Yes, please fill in the **"Investigator's statement"** at the end of visit 2 and the **"Study Conclusion Summary/Wihtdrawal/Drop-out Form"**.

ADVERSE EVENTS related to antibiotic treatment

Did any adverse event related to antibiotic treatment occur since the last visit?

❑ Yes
❑ No

If Yes, please complete the appropriate "adverse event" page and report it immediately to the Direzione Sanitaria of the Hospital and to CROM (see Study Procedures Handbook for instructions).

Centre No. |___|___|___| Screen. No. |___|___|___| Randomisation No. |___|___|___| Pat. Init. |___|___| |___|___|___|
Family name Name

VISIT 2 – Day 1

Visit date |___|___||___|___||___|___| at |___|___| |___|___|
D D M M Y Y HOUR MINUTES

OCCURRING EVENTS

PLEASE COMPLETE THE FOLLOWING TABLE:

Did the patient require <u>ventilatory support</u> since the last visit?<br><br><u>*If YES: please add ventilatory support information in the "medication since the last visit" section on page 18.</u>	❑ Yes*<br>❑ No	If YES, specify reason(s) for ventilatory support need:<br>___________________________<br>If YES, please indicate date when ventilatory support began since last visit (DD/MM/YY):<br>|___|___||___|___||___|___|
Was the patient admitted to an Intensive Care Unit <u>for any cause</u> since the last visit?	❑ Yes<br>❑ No	If YES, specify reason(s) for admission to ICU:<br>___________________________<br>If YES, please indicate date of admission to ICU since last visit (DD/MM/YY):<br>|___|___||___|___||___|___|
Did the patient have any episode(s) of <u>pulmonary embolism</u> since the last visit?	❑ Yes<br>❑ No	If YES indicate date of onset of pulmonary embolism since last visit (DD/MM/YY):<br>|___|___||___|___||___|___|
Did the patient have any <u>cardiovascular events, including acute onset pulmonary edema</u> since the last visit?	❑ Yes<br>❑ No	If YES, specify kind of cardiovascular event(s):<br>___________________________<br>If YES indicate date(s) of onset of new event(s) since last visit (DD/MM/YY):<br>1) |___|___||___|___||___|___| 2) |___|___||___|___||___|___|
Did the patient have any episodes of <u>pneumonia</u> since the last visit?	❑ Yes<br>❑ No	If YES indicate date of onset of pneumonia since last visit (DD/MM/YY):<br>|___|___||___|___||___|___|
Did the patient have <u>any other significant occurring event</u> since the last visit?	❑ Yes<br>❑ No	If YES, specify event(s):<br>___________________________<br>If YES indicate date(s) of onset of new event(s) since last visit (DD/MM/YY):<br>1) |___|___||___|___||___|___| 2) |___|___||___|___||___|___|

Has the patient experienced <u>at least one</u> of the above events <u>since the last visit</u> (i.e. did you answer YES to any of the above questions)?

No ❑ Yes* ❑

* If YES please fax a copy of this page to CROM as indicated in the Study Procedures Handbook.

Centre No. |___|___|___| Screen. No. |___|___|___| Randomisation No. |___|___|___| Pat. Init. |___|___| |___|___|___|
Family name Name

VISIT 2 – Day 1

Visit date |___|___||___|___||___|___| at |___|___| |___|___|
D D M M Y Y HOUR MINUTES

MEDICATION SINCE THE LAST VISIT

Has any medication been instituted, stopped and/or modified (dose change) since the last visit? ❑ No ❑ Yes

If YES please complete the following details, including only medications **instituted, stopped and/or modified (dose change)** *since the last visit.*

Trade name	Indication	Posology		Route	Administration (DD/MM/YY)	
		Dose/day	Unit		Start date	End date (or ONGOING)

VISIT 2 – Day 1

Visit date |___|___||___|___||___|___| at |___|___| |___|___|
D D M M Y Y HOUR MINUTES

PHYSICAL EXAMINATION

**Physical examination**	**Status**	**Comments:** if "abnormal" specify:
Cardiovascular system	❑ Abnormal<br>❑ Normal<br>❑ Not done	
Respiratory system<br>(except COPD)	❑ Abnormal<br>❑ Normal<br>❑ Not done	
Lymphatic system	❑ Abnormal<br>❑ Normal<br>❑ Not done	
Neurological-locomotor system	❑ Abnormal<br>❑ Normal<br>❑ Not done	
Skin	❑ Abnormal<br>❑ Normal<br>❑ Not done	
Endocrine	❑ Abnormal<br>❑ Normal<br>❑ Not done	
Ear-nose-throat system	❑ Abnormal<br>❑ Normal<br>❑ Not done	
Ophtalmological system	❑ Abnormal<br>❑ Normal<br>❑ Not done	
Gastrointestinal system	❑ Abnormal<br>❑ Normal<br>❑ Not done	
Others<br>……………………..	❑ Abnormal<br>❑ Normal<br>❑ Not done	

VITAL SIGNS

Body temperature |___|___|.|___|

**SITTING BLOOD PRESSURE** (mmHg)		**SITTING HEART RATE** (bpm)
**Systolic**	**Diastolic**	
|___|___|___|	|___|___|___|	|___|___|___|

Centre No. |___|___|___| Screen. No. |___|___|___| Randomisation No. |___|___|___| Pat. Init. |___|___| |___|___|___|
Family name Name

VISIT 2 – Day 1

Visit date |___|___||___|___||___|___| at |___|___| |___|___|
D D M M Y Y HOUR MINUTES

Pro-calcitonin (pro-ct)

Has the blood sample been taken for the pro-ct analysis?

Yes ❑ No ❑

If Yes, refer to the Study Procedures Handbook for handling and storage.

If No, please specify why: ____

Please fill in as soon as the results are available:

Pro-calcitonin (mcg/L) I___I___I.I___I___I

C-reactive protein (CRP), serum amyloid (SAA) and RANTES

Has the blood sample been taken for the CRP, SAA and RANTES analysis?

Yes ❑ No ❑

If Yes, refer to the Study Procedures Handbook for handling and storage.

If No, please specify why: ____

Please fill in as soon as the results are available:

CRP (mg/L) I___I___I.I___I___I

SAA (mg/L) I___I___I.I___I___I

RANTES (mcg/L) I___I___I.I___I___I

VISIT 2 – Day 1

Visit date |___|___||___|___||___|___| at |___|___| |___|___|
D D M M Y Y HOUR MINUTES

ARTERIAL BLOOD GASES

	**Yes**	**No**
Arterial blood gas analysis performed:	❑	❑
**If YES**: patient was under O_2 therapy?	❑	❑

If patient was under O_2 therapy please indicate:

- how many L/min: |___|,|___|
- how many hours/day: |___|___|

If arterial blood gas analysis was performed, indicate:

pH: |___|,|___|___|

$PaO_{2:}$ |___|___|___| mmHg

$PaCO_{2:}$ |___|___|___| mmHg

$SO_{2:}$ |___|___|___| %

$HCO_3^{-:}$ |___|___|___| mmol/L

COMPLIANCE

	Yes	No
Is patient compliant:	❑	❑

If "No", please specify:

____

____

INVESTIGATOR'S STATEMENT

I confirm that the data herein is a true complete and accurate reflection of this subject participation in the study, according to the protocol procedures.

Investigator's name: ……………………………………………………………………………

Investigator's signature: ……………………………………………………………………………

Date: |___|___||___|___||___|___|
D D M M Y Y

VISIT 3 – Day 2

Visit date |___|___||___|___||___|___| at |___|___| |___|___|
D D M M Y Y HOUR MINUTES

Has the patient withdrawn prematurely from the study?

❑ Yes
❑ No

If Yes, please fill in the **“Investigator's statement”** at the end of visit 3 and the **“Study Conclusion Summary/Wihtdrawal/Drop-out Form”**.

ADVERSE EVENTS related to antibiotic treatment

Did any adverse event related to antibiotic treatment occur since the last visit?

❑ Yes
❑ No

If Yes, please complete the appropriate “adverse event” page and report it immediately to the Direzione Sanitaria of the Hospital and to CROM (see Study Procedures Handbook for instructions).

VISIT 3 – Day 2

Visit date |___|___||___|___||___|___| at |___|___| |___|___|
D D M M Y Y HOUR MINUTES

OCCURRING EVENTS

PLEASE COMPLETE THE FOLLOWING TABLE:

Did the patient require <u>ventilatory support</u> since the last visit?<br><br><u>*If YES: please add ventilatory support information in the "medication since the last visit" section on page 24.</u>	❑ Yes*<br>❑ No	If YES, specify reason(s) for ventilatory support need:<br>___________________________<br>If YES, please indicate date when ventilatory support began since last visit (DD/MM/YY):<br>|___|___||___|___||___|___|
Was the patient admitted to an Intensive Care Unit <u>for any cause</u> since the last visit?	❑ Yes<br>❑ No	If YES, specify reason(s) for admission to ICU:<br>___________________________<br>If YES, please indicate date of admission to ICU since last visit (DD/MM/YY):<br>|___|___||___|___||___|___|
Did the patient have any episode(s) of <u>pulmonary embolism</u> since the last visit?	❑ Yes<br>❑ No	If YES indicate date of onset of pulmonary embolism since last visit (DD/MM/YY):<br>|___|___||___|___||___|___|
Did the patient have any <u>cardiovascular events, including acute onset pulmonary edema</u> since the last visit?	❑ Yes<br>❑ No	If YES, specify kind of cardiovascular event(s):<br>___________________________<br>If YES indicate date(s) of onset of new event(s) since last visit (DD/MM/YY):<br>1) |___|___||___|___||___|___| 2) |___|___||___|___||___|___|
Did the patient have any episodes of <u>pneumonia</u> since the last visit?	❑ Yes<br>❑ No	If YES indicate date of onset of pneumonia since last visit (DD/MM/YY):<br>|___|___||___|___||___|___|
Did the patient have <u>any other significant occurring event</u> since the last visit?	❑ Yes<br>❑ No	If YES, specify event(s):<br>___________________________<br>If YES indicate date(s) of onset of new event(s) since last visit (DD/MM/YY):<br>1) |___|___||___|___||___|___| 2) |___|___||___|___||___|___|

Has the patient experienced <u>at least one</u> of the above events <u>since the last visit</u> (i.e. did you answer YES to any of the above questions)?

No ❑ Yes* ❑

* If YES please fax a copy of this page to CROM as indicated in the Study Procedures Handbook.

Centre No. |___|___|___| Screen. No. |___|___|___| Randomisation No. |___|___|___| Pat. Init. |___|___| |___|___|___|
Family name Name

VISIT 3 – Day 2

Visit date |___|___||___|___||___|___| at |___|___| |___|___|
D D M M Y Y HOUR MINUTES

MEDICATION SINCE THE LAST VISIT

Has any medication been instituted, stopped and/or modified (dose change) since the last visit? ❑ No ❑ Yes

If YES please complete the following details, including only medications **instituted, stopped and/or modified (dose change)** *since the last visit.*

**Trade name**	**Indication**	**Posology**		**Route**	**Administration (DD/MM/YY)**	
		Dose/day	Unit		Start date	End date (or ONGOING)

Centre No. |___|___|___| Screen. No. |___|___|___| Randomisation No. |___|___|___| Pat. Init. |___|___| |___|___|___|
Family name Name

VISIT 3 – Day 2

Visit date |___|___||___|___||___|___| at |___|___| |___|___|
D D M M Y Y HOUR MINUTES

PHYSICAL EXAMINATION

Physical examination	Status	Comments: if "abnormal" specify:
Cardiovascular system	❑ Abnormal<br>❑ Normal<br>❑ Not done	
Respiratory system (except COPD)	❑ Abnormal<br>❑ Normal<br>❑ Not done	
Lymphatic system	❑ Abnormal<br>❑ Normal<br>❑ Not done	
Neurological-locomotor system	❑ Abnormal<br>❑ Normal<br>❑ Not done	
Skin	❑ Abnormal<br>❑ Normal<br>❑ Not done	
Endocrine	❑ Abnormal<br>❑ Normal<br>❑ Not done	
Ear-nose-throat system	❑ Abnormal<br>❑ Normal<br>❑ Not done	
Ophtalmological system	❑ Abnormal<br>❑ Normal<br>❑ Not done	
Gastrointestinal system	❑ Abnormal<br>❑ Normal<br>❑ Not done	
Others ……………………..	❑ Abnormal<br>❑ Normal<br>❑ Not done	

VITAL SIGNS

Body temperature |___|___|.|___|

SITTING BLOOD PRESSURE (mmHg)		SITTING HEART RATE (bpm)
**Systolic**	**Diastolic**	
|___|___|___|	|___|___|___|	|___|___|___|

VISIT 3 – Day 2

Visit date |___|___||___|___||___|___| at |___|___| |___|___|
D D M M Y Y HOUR MINUTES

LABORATORY TESTS

Collect blood sample for haematology and blood chemistry.
(LOCAL LABORATORY) - **Only those parameters (if any) which were abnormal at Visit 1**

**Haematology tests** *			**Clinically Significant **** Yes	No
Haemoglobin (mmol/L)		|___|___|.|___|	|___|	|___|
Haematocrit (L/L)		|___|.|___|	|___|	|___|
RBC (10^{12}/L)		|___|.|___|	|___|	|___|
WBC (10^{9}/L)		|___|___|.|___|	|___|	|___|
WBC (differential counts)	Neutrophils (%)	|___|___|.|___|	|___|	|___|
	Eosinophils (%)	|___|.|___|	|___|	|___|
	Basophils (%)	|___|.|___|	|___|	|___|
	Monocytes (%)	|___|___|.|___|	|___|	|___|
	Lymphocytes (%)	|___|___|.|___|	|___|	|___|
Platelet (10^{9}/L)		|___|___|___|	|___|	|___|

**Blood Chemistry tests** *			
Alkaline Phosphatase (U/L)	|___|___|___|.|___|	|___|	|___|
AST (SGOT) (U/L)	|___|___|.|___|	|___|	|___|
ALT (SGPT) (U/L)	|___|___|.|___|	|___|	|___|
BUN/blood urea (mmol/L)	|___|.|___|	|___|	|___|
Creatinine Kinase (U/L)	|___|___|___|.|___|	|___|	|___|
Creatinine (µmol/L)	|___|___|___|.|___|	|___|	|___|
Gamma-GT (U/L)	|___|___|.|___|	|___|	|___|
Sodium (mmol/L)	|___|___|___|.|___|	|___|	|___|
Potassium (mmol/L)	|___|.|___|	|___|	|___|
Glucose (mmol/L)	|___|.|___|___|	|___|	|___|

* to be filled in as soon as results are available

** For the out of range values, please specify if the abnormality is clinically or not clinically significant

Laboratory results need to be stored in the patient's file

Centre No. |___|___|___| Screen. No. |___|___|___| Randomisation No. |___|___|___| Pat. Init. |___|___| |___|___|___|
Family name Name

VISIT 3 – Day 2

Visit date |___|___||___|___||___|___| at |___|___| |___|___|
D D M M Y Y HOUR MINUTES

Pro-calcitonin (pro-ct)

Has the blood sample been taken for the pro-ct analysis?

Yes ❑ No ❑

If Yes, refer to the Study Procedures Handbook for handling and storage.

If No, please specify why: ____

Please fill in as soon as the results are available:

Pro-calcitonin (mcg/L) I___I___I.I___I___I

C-reactive protein (CRP), serum amyloid (SAA) and RANTES

Has the blood sample been taken for the CRP, SAA and RANTES analysis?

Yes ❑ No ❑

If Yes, refer to the Study Procedures Handbook for handling and storage.

If No, please specify why: ____

Please fill in as soon as the results are available:

CRP (mg/L) I___I___I.I___I___I

SAA (mg/L) I___I___I.I___I___I

RANTES (mcg/L) I___I___I.I___I___I

Centre No. |___|___|___| Screen. No. |___|___|___| Randomisation No. |___|___|___| Pat. Init. |___|___| |___|___|___|
Family name Name

VISIT 3 – Day 2

Visit date |___|___||___|___||___|___| at |___|___| |___|___|
D D M M Y Y HOUR MINUTES

ARTERIAL BLOOD GASES

	Yes	No
Arterial blood gas analysis performed:	❑	❑
**If YES**: patient was under O_2 therapy?	❑	❑

If patient was under O_2 therapy please indicate:

- how many L/min: |___|,|___|
- how many hours/day: |___|___|

If arterial blood gas analysis was performed, indicate:

pH: |___|,|___|___|

PaO_2: |___|___|___| mmHg

$PaCO_2$: |___|___|___| mmHg

SO_2: |___|___|___| %

HCO_3^-: |___|___|___| mmol/L

COMPLIANCE

	Yes	No
Is patient compliant:	❑	❑

If “No”, please specify:

____

____

VISIT 3 – Day 2

Visit date |___|___||___|___||___|___| at |___|___| |___|___|
D D M M Y Y HOUR MINUTES

RANDOMISATION
TO BE FILLED ON DAY 2

- **Can the subject be randomised?**

❑ Yes ❑ No

If Yes, indicate randomisation number: |___|___|___|

- **The patient has been randomized to (indicate ONLY ONE):**

❑ Pro-CT arm

❑ Standard treatment arm

Remember to send the 3 pro-CT samples (day 0, day 1 and day 2) to the Central Laboratory, University of Padua.
Shipping is via courier and takes place on day 2 as soon as the last pro-CT sample has been obtained.
Refer to the Study Procedures Handbook for instructions on shipment.

INVESTIGATOR'S STATEMENT

I confirm that the data herein is a true complete and accurate reflection of this subject participation in the study, according to the protocol procedures.

Investigator's name: ……………………………………………………………………………

Investigator's signature: ……………………………………………………………………………

Date: |___|___||___|___||___|___|
D D M M Y Y

VISIT 4 – At discharge and / or at day 10

Visit date |___|___||___|___||___|___| at |___|___| |___|___|
D D M M Y Y HOUR MINUTES

Has the patient withdrawn prematurely from the study?

❑ Yes
❑ No

If Yes, please fill in the **"Investigator's statement"** at the end of visit 4 and the **"Study Conclusion Summary/Wihtdrawal/Drop-out Form"**.

Has the patient been discharged from the Hospital?

❑ Yes
❑ No

If Yes, please specify the date: |___|___||___|___||___|___|
D D M M Y Y

ADVERSE EVENTS related to antibiotic treatment

Did any adverse event related to antibiotic treatment occur since the last visit?

❑ Yes
❑ No

If Yes, please complete the appropriate "adverse event" page and report it immediately to the Direzione Sanitaria of the Hospital and to CROM (see Study Procedures Handbook for instructions).

VISIT 4 – At discharge and / or at day 10

Visit date |___|___||___|___||___|___| at |___|___| |___|___|
D D M M Y Y HOUR MINUTES

OCCURRING EVENTS

PLEASE COMPLETE THE FOLLOWING TABLE:

Did the patient require ventilatory support since the last visit?<br><br>*If YES: please add ventilatory support information in the "medication since the last visit" section on page 32.	❑ Yes*<br>❑ No	If YES, specify reason(s) for ventilatory support need:<br>___________________________<br>If YES, please indicate date when ventilatory support began since last visit (DD/MM/YY):<br>|___|___||___|___||___|___|
Was the patient admitted to an Intensive Care Unit for any cause since the last visit?	❑ Yes<br>❑ No	If YES, specify reason(s) for admission to ICU:<br>___________________________<br>If YES, please indicate date of admission to ICU since last visit (DD/MM/YY):<br>|___|___||___|___||___|___|
Did the patient have any episode(s) of pulmonary embolism since the last visit?	❑ Yes<br>❑ No	If YES indicate date of onset of pulmonary embolism since last visit (DD/MM/YY):<br>|___|___||___|___||___|___|
Did the patient have any cardiovascular events, including acute onset pulmonary edema since the last visit?	❑ Yes<br>❑ No	If YES, specify kind of cardiovascular event(s):<br>___________________________<br>If YES indicate date(s) of onset of new event(s) since last visit (DD/MM/YY):<br>1) |___|___||___|___||___|___| 2) |___|___||___|___||___|___|
Did the patient have any episodes of pneumonia since the last visit?	❑ Yes<br>❑ No	If YES indicate date of onset of pneumonia since last visit (DD/MM/YY):<br>|___|___||___|___||___|___|
Did the patient have any other significant occurring event since the last visit?	❑ Yes<br>❑ No	If YES, specify event(s):<br>___________________________<br>If YES indicate date(s) of onset of new event(s) since last visit (DD/MM/YY):<br>1) |___|___||___|___||___|___| 2) |___|___||___|___||___|___|

Has the patient experienced at least one of the above events since the last visit (i.e. did you answer YES to any of the above questions)?

No ❑ Yes* ❑

* If YES please fax a copy of this page to CROM as indicated in the Study Procedures Handbook.

VISIT 4 – At discharge and / or at day 10

Visit date |___|___||___|___||___|___| at |___|___| |___|___|
D D M M Y Y HOUR MINUTES

MEDICATION SINCE THE LAST VISIT

Has any medication been instituted, stopped and/or modified (dose change) since the last visit?* ❑ No ❑ Yes**

* **please remember to include information on any change to antibiotic treatment**

*** If YES please complete the following details, including only medications* **instituted, stopped and/or modified (dose change)** *since the last visit.*

**Trade name**	**Indication**	**Posology**		**Route**	**Administration (DD/MM/YY)**	
		Dose/day	Unit		Start date	End date (or ONGOING)

MEDICATION AT HOSPITAL DISCHARGE

When the patient is discharged: please record in the following table all medication prescribed to the patient at the time of hospital discharge, including therapy for stable COPD and any other therapy the patient will take after leaving the hospital

**Trade name**	**Indication**	**Posology**		**Route**	**Administration (DD/MM/YY)**	
		Dose/day	Unit		Start date	End date (or ONGOING)

VISIT 4 – At discharge and / or at day 10

Visit date |___|___||___|___||___|___| at |___|___| |___|___|
D D M M Y Y HOUR MINUTES

PHYSICAL EXAMINATION

Physical examination	Status	Comments: if "abnormal" specify:
Cardiovascular system	❑ Abnormal<br>❑ Normal<br>❑ Not done	
Respiratory system<br>(except COPD)	❑ Abnormal<br>❑ Normal<br>❑ Not done	
Lymphatic system	❑ Abnormal<br>❑ Normal<br>❑ Not done	
Neurological-locomotor system	❑ Abnormal<br>❑ Normal<br>❑ Not done	
Skin	❑ Abnormal<br>❑ Normal<br>❑ Not done	
Endocrine	❑ Abnormal<br>❑ Normal<br>❑ Not done	
Ear-nose-throat system	❑ Abnormal<br>❑ Normal<br>❑ Not done	
Ophtalmological system	❑ Abnormal<br>❑ Normal<br>❑ Not done	
Gastrointestinal system	❑ Abnormal<br>❑ Normal<br>❑ Not done	
Others<br>……………………..	❑ Abnormal<br>❑ Normal<br>❑ Not done	

VITAL SIGNS

Body temperature |___|___|.|___|

SITTING BLOOD PRESSURE (mmHg)		SITTING HEART RATE (bpm)
**Systolic**	**Diastolic**	
|___|___|___|	|___|___|___|	|___|___|___|

Centre No. |___|___|___| Screen. No. |___|___|___| Randomisation No. |___|___|___| Pat. Init. |___|___| |___|___|___|
Family name Name

VISIT 4 – At discharge and / or at day 10

Visit date |___|___||___|___||___|___| at |___|___| |___|___|
D D M M Y Y HOUR MINUTES

LABORATORY TESTS

Collect blood sample for haematology and blood chemistry

(LOCAL LABORATORY)

**Haematology tests** *			**Clinically Significant** ** Yes	No
Haemoglobin (mmol/L)		|___|___|.|___|	|___|	|___|
Haematocrit (L/L)		|___|.|___|	|___|	|___|
RBC (10^{12}/L)		|___|.|___|	|___|	|___|
WBC (10^{9}/L)		|___|___|.|___|	|___|	|___|
WBC (differential counts)	Neutrophils (%)	|___|___|.|___|	|___|	|___|
	Eosinophils (%)	|___|.|___|	|___|	|___|
	Basophils (%)	|___|.|___|	|___|	|___|
	Monocytes (%)	|___|___|.|___|	|___|	|___|
	Lymphocytes (%)	|___|___|.|___|	|___|	|___|
Platelet (10^{9}/L)		|___|___|___|	|___|	|___|

Blood Chemistry tests *

Alkaline Phosphatase (U/L)	|___|___|___|.|___|	|___|	|___|
AST (SGOT) (U/L)	|___|___|.|___|	|___|	|___|
ALT (SGPT) (U/L)	|___|___|.|___|	|___|	|___|
BUN/blood urea (mmol/L)	|___|.|___|	|___|	|___|
Creatinine Kinase (U/L)	|___|___|___|.|___|	|___|	|___|
Creatinine (µmol/L)	|___|___|___|.|___|	|___|	|___|
Gamma-GT (U/L)	|___|___|.|___|	|___|	|___|
Sodium (mmol/L)	|___|___|___|.|___|	|___|	|___|
Potassium (mmol/L)	|___|.|___|	|___|	|___|
Glucose (mmol/L)	|___|.|___|___|	|___|	|___|

* to be filled in as soon as results are available

** For the out of range values, please specify if the abnormality is clinically or not clinically significant

Laboratory results need to be stored in the patient's file

VISIT 4 – At discharge and / or at day 10

Visit date |___|___||___|___||___|___| at |___|___| |___|___|
D D M M Y Y HOUR MINUTES

Pro-calcitonin (pro-ct)

Has the blood sample been taken for the pro-ct analysis?

Yes ❑ No ❑

If Yes, refer to the Study Procedures Handbook for handling and storage.

If No, please specify why: ____

Please fill in as soon as the results are available:

Pro-calcitonin (mcg/L) I___I___I.I___I___I

C-reactive protein (CRP), serum amyloid (SAA) and RANTES

Has the blood sample been taken for the CRP, SAA and RANTES analysis?

Yes ❑ No ❑

If Yes, refer to the Study Procedures Handbook for handling and storage.

If No, please specify why: ____

Please fill in as soon as the results are available:

CRP (mg/L) I___I___I.I___I___I

SAA (mg/L) I___I___I.I___I___I

RANTES (mcg/L) I___I___I.I___I___I

PTX-3 and IL1 decoy

Has the blood sample been taken for the PTX-3 and IL1 decoy analysis?

Yes ❑ No ❑

If Yes, refer to the Study Procedures Handbook for handling and storage.

If No, please specify why: ____

Please fill in as soon as the results are available:

PTX-3 (ng/ml) I___II___I___I.I___I___I

IL1 decoy (ng/ml) I___II___I___I.I___I___I

Centre No. |___|___|___| **Screen. No.** |___|___|___| **Randomisation No.** |___|___|___| **Pat. Init.** |___|___| |___|___|___|
Family name Name

VISIT 4 – At discharge and / or at day 10

Visit date |___|___||___|___||___|___| at |___|___| |___|___|
D D M M Y Y HOUR MINUTES

QUESTIONNAIRES FOR RESPIRATORY SYMPTOMS/QUALITY OF LIFE

SF36 score: total |___|___|___|

BDI score: total |___|___|___|

TDI score: total |___|___|___|

CCIQ Score: total |___|___|___|

PHARMACOECONOMIC QUESTIONNAIRE

Please remind to fill in the pharmacoeconomic questionnaire and enclose it in the plastic envelope.

SPIROMETRY

- **SPIROMETRY:** from 3 acceptable spirometric manoeuvres record the highest FEV_1 and FVC, irrespective of the manoeuvre (curve) they come from.

date |___|___| |___|___| |___|___| at |__|__| |__|__|
HOUR MINUTES

**FVC**	|___|, |___|___|___| litre
**FEV_1**	|___|, |___|___|___| litre
**FEV_1 predicted normal value**	|___|, |___|___|___| litre [ERS standards]
**FEV_1 (% predicted normal value)**	|___|___| % of predicted value
**FEV_1/FVC (%)**	|___|___|___|, |___| %

VISIT 4 – At discharge and / or at day 10

Visit date |___|___||___|___||___|___| at |___|___| |___|___|
D D M M Y Y HOUR MINUTES

MICROBIOLOGY

	Yes	No
Sputum collection performed:	❑	❑

If YES, specify volume of sputum collected: |___|,|___| ml

If NO, specify why ____

Results from local laboratory:

Bacteriology:

Identified microorganism #1: _________________________ CFU/ml: |___|___|$^{|\_\_|\_\_|}$

Identified microorganism #2: _________________________ CFU/ml: |___|___|$^{|\_\_|\_\_|}$

Identified microorganism #3: _________________________ CFU/ml: |___|___|$^{|\_\_|\_\_|}$

Serology for Intracellular pathogens:

Has the sample for serology for intracellular pathogens been obtained? Yes ❑ No ❑

If Yes, please complete below:

Was serology positive for **Mycoplasma pneumoniae**? Yes ❑ No ❑

If YES, indicate: IgM: |___|___|,|___|___|
IgG: |___|___|,|___|___|

Was serology positive for **Chlamydia**? Yes ❑ No ❑

If YES, indicate: IgM: |___|___|,|___|___|
IgG: |___|___|,|___|___|

Was serology positive for **Legionella pneumophila**? Yes ❑ No ❑

If YES, indicate:

	**POSITIVE**	**NEGATIVE**
Legionella pneumophila IgM	❑	❑
Legionella pneumophila IgG	❑	❑

VISIT 4 – At discharge and / or at day 10

Visit date |___|___||___|___||___|___| at |___|___| |___|___|
D D M M Y Y HOUR MINUTES

MICROBIOLOGY (continued)

Has the sample for **respiratory viruses RT-PCR analysis** been collected and stored at –80°C?

Yes ❑ No ❑

If **YES,** specify volume stored at -80°C for RT-PCR: |___|,|___||___||___| ml

Please remember to label the sample with the patient's ID number and store it at -80°C, as described in the Study Procedures Handbook.

Has the sample for **inflammatory cell counts** been collected? Yes ❑ No ❑

Were the slides prepared and stored at -20°C? Yes ❑ No ❑

If YES: how many slides were prepared? |___|___|

Centre No. |___|___|___| Screen. No. |___|___|___| Randomisation No. |___|___|___| Pat. Init. |___|___| |___|___|___|
Family name Name

VISIT 4 – At discharge and / or at day 10

Visit date |___|___||___|___||___|___| at |___|___| |___|___|
D D M M Y Y HOUR MINUTES

ARTERIAL BLOOD GASES

	**Yes**	**No**
Arterial blood gas analysis performed:	❑	❑
**If YES**: patient was under O_2 therapy?	❑	❑

If patient was under O_2 therapy please indicate:

- how many L/min: |___|,|___|
- how many hours/day: |___|___|

If arterial blood gas analysis was performed, indicate:

pH: |___|,|___|___|

$PaO_{2:}$ |___|___|___| mmHg

$PaCO_{2:}$ |___|___|___| mmHg

$SO_{2:}$ |___|___|___| %

$HCO_3^{-:}$ |___|___|___| mmol/L

COMPLIANCE

	Yes	No
Is patient compliant:	❑	❑

If "No", please specify:

____

____

INVESTIGATOR'S STATEMENT

I confirm that the data herein is a true complete and accurate reflection of this subject participation in the study, according to the protocol procedures.

Investigator's name: ……………………………………………………………………………

Investigator's signature: ……………………………………………………………………………

Date: |___|___||___|___||___|___|
D D M M Y Y

VISIT 5 – Day 30

Visit date |___|___||___|___||___|___| at |___|___| |___|___|
D D M M Y Y HOUR MINUTES

Has the patient withdrawn prematurely from the study?

❑ Yes
❑ No

If Yes, please fill in the **“Investigator's statement”** at the end of visit 5 and the **“Study Conclusion Summary/Wihtdrawal/Drop-out Form”**.

Has the patient been discharged from the Hospital?*

❑ Yes
❑ No

If Yes, please specify the date: |___|___||___|___||___|___|
D D M M Y Y

***** please complete only if patient had not been discharged by Visit 4

Centre No. |___|___|___| Screen. No. |___|___|___| Randomisation No. |___|___|___| Pat. Init. |___|___| |___|___|___|
Family name Name

VISIT 5 – Day 30

Visit date |___|___||___|___||___|___| at |___|___| |___|___|
D D M M Y Y HOUR MINUTES

OCCURRING EVENTS

PLEASE COMPLETE THE FOLLOWING TABLE:

Did the patient have at least one COPD exacerbation* since the last visit?<br>(*defined as:<br>- worsening of respiratory symptoms requiring treatment with antibiotics and/or systemic steroids since last visit<br>- and/or new hospital admission for a COPD exacerbation since last visit<br>- and/or need for ventilatory support or intubation, or death since last visit)	❑ Yes<br>❑ No	If YES indicate date(s) of onset of new COPD exacerbation(s) since last visit (DD/MM/YY):<br>1) |___|___||___|___||___|___| 2) |___|___||___|___||___|___| 3) |___|___||___|___||___|___|
Was the patient hospitalized <u>for a new COPD exacerbation</u> since the last visit?	❑ Yes<br>❑ No	If YES indicate date(s) of new hospitalization(s) for COPD exacerbation(s) since last visit (from DD/MM/YY to DD/MM/YY):<br>1) from |___|___||___|___||___|___| to |___|___||___|___||___|___| 2) from |___|___||___|___||___|___| to |___|___||___|___||___|___| 3) from |___|___||___|___||___|___| to |___|___||___|___||___|___|
Was the patient hospitalized <u>for any other cause</u> (except COPD) since the last visit?	❑ Yes<br>❑ No	If YES, specify reason(s) for hospitalization(s):<br>____________________________<br>If YES, please indicate date(s) of new hospitalization(s) since last visit (from DD/MM/YY to DD/MM/YY):<br>1) from |___|___||___|___||___|___| to |___|___||___|___||___|___| 2) from |___|___||___|___||___|___| to |___|___||___|___||___|___| 3) from |___|___||___|___||___|___| to |___|___||___|___||___|___|
Was the patient admitted to an Intensive Care Unit <u>for any cause</u> since the last visit?	❑ Yes<br>❑ No	If YES, specify reason(s) for admission(s) to ICU:<br>____________________________<br>If YES, please indicate date(s) of admission(s) to ICU since last visit (from DD/MM/YY to DD/MM/YY):<br>1) from |___|___||___|___||___|___| to |___|___||___|___||___|___| 2) from |___|___||___|___||___|___| to |___|___||___|___||___|___| 3) from |___|___||___|___||___|___| to |___|___||___|___||___|___|

VISIT 5 – Day 30

Visit date |___|___||___|___||___|___| at |___|___| |___|___|
D D M M Y Y HOUR MINUTES

OCCURRING EVENTS (continued)

Did the patient have any episode(s) of <u>pulmonary embolism</u> since the last visit?	❑ Yes<br>❑ No	If YES indicate date(s) of onset of episode(s) of pulmonary embolism since last visit (DD/MM/YY):<br>1) |___|___||___|___||___|___| 2) |___|___||___|___||___|___| 3) |___|___||___|___||___|___|
Did the patient have any <u>cardiovascular events, including acute onset pulmonary edema</u> since the last visit?	❑ Yes<br>❑ No	If YES, specify kind of cardiovascular event(s):<br>_________________________<br>If YES indicate date(s) of onset of new event(s) since last visit (DD/MM/YY):<br>1) |___|___||___|___||___|___| 2) |___|___||___|___||___|___| 3) |___|___||___|___||___|___|
Did the patient have any episodes of <u>pneumonia</u> since the last visit?	❑ Yes<br>❑ No	If YES indicate date(s) of onset of pneumonia since last visit (DD/MM/YY):<br>1) |___|___||___|___||___|___| 2) |___|___||___|___||___|___| 3) |___|___||___|___||___|___|
Did the patient have <u>any other significant occurring event</u> since the last visit?	❑ Yes<br>❑ No	If YES, specify event(s):<br>____________________________<br>If YES indicate date(s) of onset of new event(s) since last visit (DD/MM/YY):<br>1) |___|___||___|___||___|___| 2) |___|___||___|___||___|___| 3) |___|___||___|___||___|___|

Has the patient experienced <u>at least one</u> of the above events <u>since the last visit</u> (i.e. did you answer YES to any of the above questions)?

No ❑ Yes* ❑

* If YES please fax a copy of pages 41-42 to CROM as indicated in the Study Procedures Handbook.

VISIT 5 – Day 30

Visit date |___|___||___|___||___|___| at |___|___| |___|___|
D D M M Y Y HOUR MINUTES

MEDICATION SINCE THE LAST VISIT

Has any medication been instituted, stopped and/or modified (dose change) since the last visit? ❑ No ❑ Yes

If YES please complete the following details, including only medications **instituted, stopped and/or modified (dose change)** *since the last visit.*

**Trade name**	**Indication**	**Posology**		**Route**	**Administration (DD/MM/YY)**	
		Dose/day	Unit		Start date	End date (or ONGOING)

VISIT 5 – Day 30

Visit date |___|___||___|___||___|___| at |___|___| |___|___|
D D M M Y Y HOUR MINUTES

PHYSICAL EXAMINATION

Physical examination	Status	Comments: if "abnormal" specify:
Cardiovascular system	❑ Abnormal<br>❑ Normal<br>❑ Not done	
Respiratory system (except COPD)	❑ Abnormal<br>❑ Normal<br>❑ Not done	
Lymphatic system	❑ Abnormal<br>❑ Normal<br>❑ Not done	
Neurological-locomotor system	❑ Abnormal<br>❑ Normal<br>❑ Not done	
Skin	❑ Abnormal<br>❑ Normal<br>❑ Not done	
Endocrine	❑ Abnormal<br>❑ Normal<br>❑ Not done	
Ear-nose-throat system	❑ Abnormal<br>❑ Normal<br>❑ Not done	
Ophtalmological system	❑ Abnormal<br>❑ Normal<br>❑ Not done	
Gastrointestinal system	❑ Abnormal<br>❑ Normal<br>❑ Not done	
Others ……………………..	❑ Abnormal<br>❑ Normal<br>❑ Not done	

VITAL SIGNS

SITTING BLOOD PRESSURE (mmHg)		SITTING HEART RATE (bpm)
Systolic	Diastolic	
|___|___|___|	|___|___|___|	|___|___|___|

VISIT 5 – Day 30

Visit date |___|___||___|___||___|___| at |___|___| |___|___|
D D M M Y Y HOUR MINUTES

Pro-calcitonin (pro-ct)

Has the blood sample been taken for the pro-ct analysis?

Yes ❑ No ❑

If Yes, refer to the Study Procedures Handbook for handling and storage.

If No, please specify why: ______________________________________

Please fill in as soon as the results are available:

Pro-calcitonin (mcg/L) I___I___I.I___I___I

C-reactive protein (CRP), serum amyloid (SAA) and RANTES

Has the blood sample been taken for the CRP, SAA and RANTES analysis?

Yes ❑ No ❑

If Yes, refer to the Study Procedures Handbook for handling and storage.

If No, please specify why: ______________________________________

Please fill in as soon as the results are available:

CRP (mg/L) I___I___I.I___I___I

SAA (mg/L) I___I___I.I___I___I

RANTES (mcg/L) I___I___I.I___I___I

VISIT 5 – Day 30

Visit date |___|___||___|___||___|___| at |___|___| |___|___|
D D M M Y Y HOUR MINUTES

QUESTIONNAIRES FOR RESPIRATORY SYMPTOMS/QUALITY OF LIFE

SF36 score: total |___|___|___|

BDI score: total |___|___|___|

TDI score: total |___|___|___|

CCIQ Score: total |___|___|___|

PHARMACOECONOMIC QUESTIONNAIRE

Please remind to fill in the pharmacoeconomic questionnaire and enclose it in the plastic envelope.

SPIROMETRY

- **SPIROMETRY:** from 3 acceptable spirometric manoeuvres record the highest FEV_1 and FVC, irrespective of the manoeuvre (curve) they come from.

date |___|___| |___|___| |___|___| at |__|__| |__|__|
HOUR MINUTES

**FVC**	|___|, |___|___|___| litre
**FEV_1**	|___|, |___|___|___| litre
**FEV_1 predicted normal value**	|___|, |___|___|___| litre [ERS standards]
**FEV_1 (% predicted normal value)**	|___|___| % of predicted value
**FEV_1/FVC (%)**	|___|___|___|, |___| %

VISIT 5 – Day 30

Visit date |___|___||___|___||___|___| at |___|___| |___|___|
D D M M Y Y HOUR MINUTES

MICROBIOLOGY

	Yes	No
Sputum collection performed:	❑	❑

If YES, specify volume of sputum collected: |___|,|___| ml

If NO, specify why ____

Results from local laboratory:

Bacteriology:

Identified microorganism #1: ____________________________ CFU/ml: |___|___|$^{|\_\_|\_\_|}$

Identified microorganism #2: ____________________________ CFU/ml: |___|___|$^{|\_\_|\_\_|}$

Identified microorganism #3: ____________________________ CFU/ml: |___|___|$^{|\_\_|\_\_|}$

Serology for Intracellular pathogens:

Has the sample for serology for intracellular pathogens been obtained? Yes ❑ No ❑

If Yes, please complete below:

Was serology positive for **Mycoplasma pneumoniae**? Yes ❑ No ❑

If YES, indicate: IgM: |___|___|,|___|___|
IgG: |___|___|,|___|___|

Was serology positive for **Chlamydia**? Yes ❑ No ❑

If YES, indicate: IgM: |___|___|,|___|___|
IgG: |___|___|,|___|___|

Was serology positive for **Legionella pneumophila**? Yes ❑ No ❑

If YES, indicate:

	**POSITIVE**	**NEGATIVE**
Legionella pneumophila IgM	❑	❑
Legionella pneumophila IgG	❑	❑

VISIT 5 – Day 30

Visit date |___|___||___|___||___|___| at |___|___| |___|___|
D D M M Y Y HOUR MINUTES

MICROBIOLOGY (continued)

Has the sample for **respiratory viruses RT-PCR analysis** been collected and stored at –80°C?

Yes ❑ No ❑

If **YES,** specify volume stored at -80°C for RT-PCR: |___|,|___||___||___| ml

Please remember to label the sample with the patient's ID number and store it at -80°C, as described in the Study Procedures Handbook.

Has the sample for **inflammatory cell counts** been collected? Yes ❑ No ❑

Were the slides prepared and stored at -20°C? Yes ❑ No ❑

If YES: how many slides were prepared? |___|___|

VISIT 5 – Day 30

Visit date |___|___||___|___||___|___| at |___|___| |___|___|
D D M M Y Y HOUR MINUTES

ARTERIAL BLOOD GASES

	Yes	No
Arterial blood gas analysis performed:	❑	❑
**If YES**: patient was under O_2 therapy?	❑	❑

If patient was under O_2 therapy please indicate:

- how many L/min: |___|,|___|
- how many hours/day: |___|___|

If arterial blood gas analysis was performed, indicate:

pH: |___|,|___|___|

PaO_2: |___|___|___| mmHg

$PaCO_2$: |___|___|___| mmHg

SO_2: |___|___|___| %

HCO_3^-: |___|___|___| mmol/L

INVESTIGATOR'S STATEMENT

I confirm that the data herein is a true complete and accurate reflection of this subject participation in the study, according to the protocol procedures.

Investigator's name: ……………………………………………………………………………

Investigator's signature: ……………………………………………………………………………

Date: |___|___||___|___||___|___|
D D M M Y Y

Centre No. |___|___|___| Screen. No. |___|___|___| Randomisation No. |___|___|___| Pat. Init. |___|___| |___|___|___|
Family name Name

TELEPHONE CONTACT – Day 60

Visit date |___|___||___|___||___|___| at |___|___| |___|___|
D D M M Y Y HOUR MINUTES

CLINICAL STATUS

Collect information on	Status	Comments: if "abnormal" specify:
1. Cardiovascular system	❑ Abnormal<br>❑ Normal<br>❑ Not done	
2. Respiratory system (except COPD)	❑ Abnormal<br>❑ Normal<br>❑ Not done	
3. Lymphatic system	❑ Abnormal<br>❑ Normal<br>❑ Not done	
4. Neurological-locomotor system	❑ Abnormal<br>❑ Normal<br>❑ Not done	
5. Skin	❑ Abnormal<br>❑ Normal<br>❑ Not done	
6. Endocrine	❑ Abnormal<br>❑ Normal<br>❑ Not done	
7. Ear-nose-throat system	❑ Abnormal<br>❑ Normal<br>❑ Not done	
8. Ophtalmological system	❑ Abnormal<br>❑ Normal<br>❑ Not done	
9. Gastrointestinal system	❑ Abnormal<br>❑ Normal<br>❑ Not done	
10. Others ……………………..	❑ Abnormal<br>❑ Normal<br>❑ Not done	

Centre No. |___|___|___| Screen. No. |___|___|___| Randomisation No. |___|___|___| Pat. Init. |___|___| |___|___|___|
Family name Name

TELEPHONE CONTACT – Day 60

Visit date |___|___||___|___||___|___| at |___|___| |___|___|
D D M M Y Y HOUR MINUTES

OCCURRING EVENTS

PLEASE COMPLETE THE FOLLOWING TABLE:

Did the patient have at least one COPD exacerbation* since the last visit?<br>(*defined as:<br>- worsening of respiratory symptoms requiring treatment with antibiotics and/or systemic steroids since last visit<br>- and/or new hospital admission for a COPD exacerbation since last visit<br>- and/or need for ventilatory support or intubation, or death since last visit)	❑ Yes<br>❑ No	If YES indicate date(s) of onset of new COPD exacerbation(s) since last visit (DD/MM/YY):<br>1) |___|___||___|___||___|___| 2) |___|___||___|___||___|___| 3) |___|___||___|___||___|___|
Was the patient hospitalized <u>for a new COPD exacerbation</u> since the last visit?	❑ Yes<br>❑ No	If YES indicate date(s) of new hospitalization(s) for COPD exacerbation(s) since last visit (from DD/MM/YY to DD/MM/YY):<br>1) from |___|___||___|___||___|___| to |___|___||___|___||___|___| 2) from |___|___||___|___||___|___| to |___|___||___|___||___|___| 3) from |___|___||___|___||___|___| to |___|___||___|___||___|___|
Was the patient hospitalized <u>for any other cause</u> (except COPD) since the last visit?	❑ Yes<br>❑ No	If YES, specify reason(s) for hospitalization(s):<br>____________________________<br>If YES, please indicate date(s) of new hospitalization(s) since last visit (from DD/MM/YY to DD/MM/YY):<br>1) from |___|___||___|___||___|___| to |___|___||___|___||___|___| 2) from |___|___||___|___||___|___| to |___|___||___|___||___|___| 3) from |___|___||___|___||___|___| to |___|___||___|___||___|___|
Was the patient admitted to an Intensive Care Unit <u>for any cause</u> since the last visit?	❑ Yes<br>❑ No	If YES, specify reason(s) for admission(s) to ICU:<br>____________________________<br>If YES, please indicate date(s) of admission(s) to ICU since last visit (from DD/MM/YY to DD/MM/YY):<br>1) from |___|___||___|___||___|___| to |___|___||___|___||___|___| 2) from |___|___||___|___||___|___| to |___|___||___|___||___|___| 3) from |___|___||___|___||___|___| to |___|___||___|___||___|___|

TELEPHONE CONTACT – Day 60

Visit date |___|___||___|___||___|___| at |___|___| |___|___|
D D M M Y Y HOUR MINUTES

OCCURRING EVENTS (continued)

Did the patient have any episode(s) of <u>pulmonary embolism</u> since the last visit?	❑ Yes<br>❑ No	If YES indicate date(s) of onset of episode(s) of pulmonary embolism since last visit (DD/MM/YY):<br>1) |___|___||___|___||___|___| 2) |___|___||___|___||___|___| 3) |___|___||___|___||___|___|
Did the patient have any <u>cardiovascular events, including acute onset pulmonary edema</u> since the last visit?	❑ Yes<br>❑ No	If YES, specify kind of cardiovascular event(s):<br>________________________<br>If YES indicate date(s) of onset of new event(s) since last visit (DD/MM/YY):<br>1) |___|___||___|___||___|___| 2) |___|___||___|___||___|___| 3) |___|___||___|___||___|___|
Did the patient have any episodes of <u>pneumonia</u> since the last visit?	❑ Yes<br>❑ No	If YES indicate date(s) of onset of pneumonia since last visit (DD/MM/YY):<br>1) |___|___||___|___||___|___| 2) |___|___||___|___||___|___| 3) |___|___||___|___||___|___|
Did the patient have <u>any other significant occurring event</u> since the last visit?	❑ Yes<br>❑ No	If YES, specify event(s):<br>__________________________<br>If YES indicate date(s) of onset of new event(s) since last visit (DD/MM/YY):<br>1) |___|___||___|___||___|___| 2) |___|___||___|___||___|___| 3) |___|___||___|___||___|___|

Has the patient experienced <u>at least one</u> of the above events <u>since the last visit</u> (i.e. did you answer YES to any of the above questions)?

No ❑ Yes* ❑

* If YES please fax a copy of pages 51-52 to CROM as indicated in the Study Procedures Handbook.

Centre No. |___|___|___| Screen. No. |___|___|___| Randomisation No. |___|___|___| Pat. Init. |___|___| |___|___|___|
Family name Name

TELEPHONE CONTACT – Day 60

Visit date |___|___||___|___||___|___| at |___|___| |___|___|
D D M M Y Y HOUR MINUTES

MEDICATION SINCE THE LAST VISIT

Has any medication been instituted, stopped and/or modified (dose change) since the last visit? ❑ No ❑ Yes

If YES please complete the following details, including only medications **instituted, stopped and/or modified (dose change)** *since the last visit.*

Trade name	Indication	Posology		Route	Administration (DD/MM/YY)	
		Dose/day	Unit		Start date	End date (or ONGOING)

PHARMACOECONOMIC QUESTIONNAIRE

Please remind to fill in the pharmacoeconomic questionnaire and enclose it in the plastic envelope

INVESTIGATOR'S STATEMENT

I confirm that the data herein is a true complete and accurate reflection of this subject participation in the study, according to the protocol procedures.

Investigator's name: …………………………………………………………………………

Investigator's signature: …………………………………………………………………………

Date: |___|___||___|___||___|___|
D D M M Y Y

Centre No. |___|___|___| Screen. No. |___|___|___| Randomisation No. |___|___|___| Pat. Init. |___|___| |___|___|___|
Family name Name

VISIT 6 – Day 90

Visit date |___|___||___|___||___|___| at |___|___| |___|___|
D D M M Y Y HOUR MINUTES

Has the patient withdrawn prematurely from the study?

❑ Yes
❑ No

If Yes, please fill in the **“Investigator's statement”** at the end of visit 6 and the **“Study Conclusion Summary/Wihtdrawal/Drop-out Form”**.

Centre No. |___|___|___| Screen. No. |___|___|___| Randomisation No. |___|___|___| Pat. Init. |___|___| |___|___|___|
Family name Name

VISIT 6 – Day 90

Visit date |___|___||___|___||___|___| at |___|___| |___|___|
D D M M Y Y HOUR MINUTES

OCCURRING EVENTS

PLEASE COMPLETE THE FOLLOWING TABLE:

Did the patient have at least one COPD exacerbation* since the last visit?<br>(*defined as:<br>- worsening of respiratory symptoms requiring treatment with antibiotics and/or systemic steroids since last visit<br>- and/or new hospital admission for a COPD exacerbation since last visit<br>- and/or need for ventilatory support or intubation, or death since last visit)	❑ Yes<br>❑ No	If YES indicate date(s) of onset of new COPD exacerbation(s) since last visit (DD/MM/YY):<br>1) |___|___||___|___||___|___| 2) |___|___||___|___||___|___| 3) |___|___||___|___||___|___|
Was the patient hospitalized <u>for a new COPD exacerbation</u> since the last visit?	❑ Yes<br>❑ No	If YES indicate date(s) of new hospitalization(s) for COPD exacerbation(s) since last visit (from DD/MM/YY to DD/MM/YY):<br>1) from |___|___||___|___||___|___| to |___|___||___|___||___|___| 2) from |___|___||___|___||___|___| to |___|___||___|___||___|___| 3) from |___|___||___|___||___|___| to |___|___||___|___||___|___|
Was the patient hospitalized <u>for any other cause</u> (except COPD) since the last visit?	❑ Yes<br>❑ No	If YES, specify reason(s) for hospitalization(s):<br>______________________________<br>If YES, please indicate date(s) of new hospitalization(s) since last visit<br>(from DD/MM/YY to DD/MM/YY):<br>1) from |___|___||___|___||___|___| to |___|___||___|___||___|___| 2) from |___|___||___|___||___|___| to |___|___||___|___||___|___| 3) from |___|___||___|___||___|___| to |___|___||___|___||___|___|
Was the patient admitted to an Intensive Care Unit <u>for any cause</u> since the last visit?	❑ Yes<br>❑ No	If YES, specify reason(s) for admission(s) to ICU:<br>______________________________<br>If YES, please indicate date(s) of admission(s) to ICU since last visit<br>(from DD/MM/YY to DD/MM/YY):<br>1) from |___|___||___|___||___|___| to |___|___||___|___||___|___| 2) from |___|___||___|___||___|___| to |___|___||___|___||___|___| 3) from |___|___||___|___||___|___| to |___|___||___|___||___|___|

VISIT 6 – Day 90

Visit date |___|___||___|___||___|___| at |___|___| |___|___|
D D M M Y Y HOUR MINUTES

OCCURRING EVENTS (continued)

Did the patient have any episode(s) of <u>pulmonary embolism</u> since the last visit?	❑ Yes<br>❑ No	If YES indicate date(s) of onset of episode(s) of pulmonary embolism since last visit (DD/MM/YY):<br>1) |___|___||___|___||___|___| 2) |___|___||___|___||___|___| 3) |___|___||___|___||___|___|
Did the patient have any <u>cardiovascular events, including acute onset pulmonary edema</u> since the last visit?	❑ Yes<br>❑ No	If YES, specify kind of cardiovascular event(s):<br>________________________<br>If YES indicate date(s) of onset of new event(s) since last visit (DD/MM/YY):<br>1) |___|___||___|___||___|___| 2) |___|___||___|___||___|___| 3) |___|___||___|___||___|___|
Did the patient have any episodes of <u>pneumonia</u> since the last visit?	❑ Yes<br>❑ No	If YES indicate date(s) of onset of pneumonia since last visit (DD/MM/YY):<br>1) |___|___||___|___||___|___| 2) |___|___||___|___||___|___| 3) |___|___||___|___||___|___|
Did the patient have <u>any other significant occurring event</u> since the last visit?	❑ Yes<br>❑ No	If YES, specify event(s):<br>__________________________<br>If YES indicate date(s) of onset of new event(s) since last visit (DD/MM/YY):<br>1) |___|___||___|___||___|___| 2) |___|___||___|___||___|___| 3) |___|___||___|___||___|___|

Has the patient experienced <u>at least one</u> of the above events <u>since the last visit</u> (i.e. did you answer YES to any of the above questions)?

No ❑ Yes* ❑

* If YES please fax a copy of pages 55-56 to CROM as indicated in the Study Procedures Handbook.

Centre No. |___|___|___| Screen. No. |___|___|___| Randomisation No. |___|___|___| Pat. Init. |___|___| |___|___|___|
Family name Name

VISIT 6 – Day 90

Visit date |___|___||___|___||___|___| at |___|___| |___|___|
D D M M Y Y HOUR MINUTES

MEDICATION SINCE THE LAST VISIT

Has any medication been instituted, stopped and/or modified (dose change) since the last visit? ❑ No ❑ Yes

If YES please complete the following details, including only medications **instituted, stopped and/or modified (dose change)** *since the last visit.*

**Trade name**	**Indication**	**Posology**		**Route**	**Administration (DD/MM/YY)**	
		Dose/day	Unit		Start date	End date (or ONGOING)

Centre No. |___|___|___| Screen. No. |___|___|___| Randomisation No. |___|___|___| Pat. Init. |___|___| |___|___|___|
Family name Name

VISIT 6 – Day 90

Visit date |___|___||___|___||___|___| at |___|___| |___|___|
D D M M Y Y HOUR MINUTES

PHYSICAL EXAMINATION

Physical examination	Status	Comments: if "abnormal" specify:
Cardiovascular system	❑ Abnormal<br>❑ Normal<br>❑ Not done	
Respiratory system (except COPD)	❑ Abnormal<br>❑ Normal<br>❑ Not done	
Lymphatic system	❑ Abnormal<br>❑ Normal<br>❑ Not done	
Neurological-locomotor system	❑ Abnormal<br>❑ Normal<br>❑ Not done	
Skin	❑ Abnormal<br>❑ Normal<br>❑ Not done	
Endocrine	❑ Abnormal<br>❑ Normal<br>❑ Not done	
Ear-nose-throat system	❑ Abnormal<br>❑ Normal<br>❑ Not done	
Ophtalmological system	❑ Abnormal<br>❑ Normal<br>❑ Not done	
Gastrointestinal system	❑ Abnormal<br>❑ Normal<br>❑ Not done	
Others ……………………..	❑ Abnormal<br>❑ Normal<br>❑ Not done	

VITAL SIGNS

SITTING BLOOD PRESSURE (mmHg)		SITTING HEART RATE (bpm)
Systolic	Diastolic	
|___|___|___|	|___|___|___|	|___|___|___|

VISIT 6 – Day 90

Visit date |___|___||___|___||___|___| at |___|___| |___|___|
D D M M Y Y HOUR MINUTES

LABORATORY TESTS

Collect blood sample for haematology and blood chemistry.
LOCAL LABORATORY

**Haematology tests** *			**Clinically Significant** ** Yes	No
Haemoglobin (mmol/L)		|___|___|.|___|	|___|	|___|
Haematocrit (L/L)		|___|.|___|	|___|	|___|
RBC (10^{12}/L)		|___|.|___|	|___|	|___|
WBC (10^{9}/L)		|___|___|.|___|	|___|	|___|
WBC (differential counts)	Neutrophils (%)	|___|___|.|___|	|___|	|___|
	Eosinophils (%)	|___|.|___|	|___|	|___|
	Basophils (%)	|___|.|___|	|___|	|___|
	Monocytes (%)	|___|___|.|___|	|___|	|___|
	Lymphocytes (%)	|___|___|.|___|	|___|	|___|
Platelet (10^{9}/L)		|___|___|___|	|___|	|___|

Blood Chemistry tests *

Alkaline Phosphatase (U/L)	|___|___|___|.|___|	|___|	|___|
AST (SGOT) (U/L)	|___|___|.|___|	|___|	|___|
ALT (SGPT) (U/L)	|___|___|.|___|	|___|	|___|
BUN/blood urea (mmol/L)	|___|.|___|	|___|	|___|
Creatinine Kinase (U/L)	|___|___|___|.|___|	|___|	|___|
Creatinine (µmol/L)	|___|___|___|.|___|	|___|	|___|
Gamma-GT (U/L)	|___|___|.|___|	|___|	|___|
Sodium (mmol/L)	|___|___|___|.|___|	|___|	|___|
Potassium (mmol/L)	|___|.|___|	|___|	|___|
Glucose (mmol/L)	|___|.|___|___|	|___|	|___|

* to be filled in as soon as results are available

** For the out of range values, please specify if the abnormality is clinically or not clinically significant

Laboratory results need to be stored in the patient's file

VISIT 6 – Day 90

Visit date |___|___||___|___||___|___| at |___|___| |___|___|
D D M M Y Y HOUR MINUTES

Pro-calcitonin (pro-ct)

Has the blood sample been taken for the pro-ct analysis?

Yes ❑ No ❑

If Yes, refer to the Study Procedures Handbook for handling and storage.

If No, please specify why: ______________________________________

Please fill in as soon as the results are available:

Pro-calcitonin (mcg/L) I___I___I.I___I___I

C-reactive protein (CRP), serum amyloid (SAA) and RANTES

Has the blood sample been taken for the CRP, SAA and RANTES analysis?

Yes ❑ No ❑

If Yes, refer to the Study Procedures Handbook for handling and storage.

If No, please specify why: ______________________________________

Please fill in as soon as the results are available:

CRP (mg/L) I___I___I.I___I___I

SAA (mg/L) I___I___I.I___I___I

RANTES (mcg/L) I___I___I.I___I___I

PTX-3 and IL1 decoy

Has the blood sample been taken for the PTX-3 and IL1 decoy analysis?

Yes ❑ No ❑

If Yes, refer to the Study Procedures Handbook for handling and storage.

If No, please specify why: ______________________________________

Please fill in as soon as the results are available:

PTX-3 (ng/ml) I___II___I___I.I___I___I

IL1 decoy (ng/ml) I___II___I___I.I___I___I

VISIT 6 – Day 90

Visit date |___|___||___|___||___|___| at |___|___| |___|___|
D D M M Y Y HOUR MINUTES

QUESTIONNAIRES FOR RESPIRATORY SYMPTOMS/QUALITY OF LIFE

SF36 score: total |___|___|___|

BDI score: total |___|___|___|

TDI score: total |___|___|___|

CCIQ Score: total |___|___|___|

PHARMACOECONOMIC QUESTIONNAIRE

Please remind to fill in the pharmacoeconomic questionnaire and enclose it in the plastic envelope.

SPIROMETRY

- **SPIROMETRY:** from 3 acceptable spirometric manoeuvres record the highest FEV_1 and FVC, irrespective of the manoeuvre (curve) they come from.

date |___|___| |___|___| |___|___| at |__|__| |__|__|
HOUR MINUTES

**FVC**	|___|, |___|___|___| litre
**FEV_1**	|___|, |___|___|___| litre
**FEV_1 predicted normal value**	|___|, |___|___|___| litre [ERS standards]
**FEV_1 (% predicted normal value)**	|___|___| % of predicted value
**FEV_1/FVC (%)**	|___|___|___|, |___| %

VISIT 6 – Day 90

Visit date |___|___||___|___||___|___| at |___|___| |___|___|
D D M M Y Y HOUR MINUTES

ARTERIAL BLOOD GASES

	Yes	No
Arterial blood gas analysis performed:	❑	❑
**If YES**: patient was under O_2 therapy?	❑	❑

If patient was under O_2 therapy please indicate:

- how many L/min: |___|,|___|
- how many hours/day: |___|___|

If arterial blood gas analysis was performed, indicate:

pH: |___|,|___|___|

$PaO_{2:}$ |___|___|___| mmHg

$PaCO_{2:}$ |___|___|___| mmHg

$SO_{2:}$ |___|___|___| %

$HCO_3^{-:}$ |___|___|___| mmol/L

INVESTIGATOR'S STATEMENT

I confirm that the data herein is a true complete and accurate reflection of this subject participation in the study, according to the protocol procedures.

Investigator's name: ……………………………………………………………………………

Investigator's signature: ……………………………………………………………………………

Date: |___|___||___|___||___|___|
D D M M Y Y

TELEPHONE CONTACT – Day 120

Visit date |___|___||___|___||___|___| at |___|___| |___|___|
D D M M Y Y HOUR MINUTES

CLINICAL STATUS

Collect information on	Status	Comments: if "abnormal" specify:
1. Cardiovascular system	❑ Abnormal<br>❑ Normal<br>❑ Not done	
2. Respiratory system (except COPD)	❑ Abnormal<br>❑ Normal<br>❑ Not done	
3. Lymphatic system	❑ Abnormal<br>❑ Normal<br>❑ Not done	
4. Neurological-locomotor system	❑ Abnormal<br>❑ Normal<br>❑ Not done	
5. Skin	❑ Abnormal<br>❑ Normal<br>❑ Not done	
6. Endocrine	❑ Abnormal<br>❑ Normal<br>❑ Not done	
7. Ear-nose-throat system	❑ Abnormal<br>❑ Normal<br>❑ Not done	
8. Ophtalmological system	❑ Abnormal<br>❑ Normal<br>❑ Not done	
9. Gastrointestinal system	❑ Abnormal<br>❑ Normal<br>❑ Not done	
10. Others ……………………..	❑ Abnormal<br>❑ Normal<br>❑ Not done	

Centre No. |___|___|___| Screen. No. |___|___|___| Randomisation No. |___|___|___| Pat. Init. |___|___| |___|___|___|
Family name Name

TELEPHONE CONTACT – Day 120

Visit date |___|___||___|___||___|___| at |___|___| |___|___|
D D M M Y Y HOUR MINUTES

OCCURRING EVENTS

PLEASE COMPLETE THE FOLLOWING TABLE:

Did the patient have at least one COPD exacerbation* since the last visit?<br>(*defined as:<br>- worsening of respiratory symptoms requiring treatment with antibiotics and/or systemic steroids since last visit<br>- and/or new hospital admission for a COPD exacerbation since last visit<br>- and/or need for ventilatory support or intubation, or death since last visit)	❑ Yes<br>❑ No	If YES indicate date(s) of onset of new COPD exacerbation(s) since last visit (DD/MM/YY):<br>1) |___|___||___|___||___|___| 2) |___|___||___|___||___|___| 3) |___|___||___|___||___|___|
Was the patient hospitalized <u>for a new COPD exacerbation</u> since the last visit?	❑ Yes<br>❑ No	If YES indicate date(s) of new hospitalization(s) for COPD exacerbation(s) since last visit (from DD/MM/YY to DD/MM/YY):<br>1) from |___|___||___|___||___|___| to |___|___||___|___||___|___| 2) from |___|___||___|___||___|___| to |___|___||___|___||___|___| 3) from |___|___||___|___||___|___| to |___|___||___|___||___|___|
Was the patient hospitalized <u>for any other cause</u> (except COPD) since the last visit?	❑ Yes<br>❑ No	If YES, specify reason(s) for hospitalization(s):<br>____________________________<br>If YES, please indicate date(s) of new hospitalization(s) since last visit (from DD/MM/YY to DD/MM/YY):<br>1) from |___|___||___|___||___|___| to |___|___||___|___||___|___| 2) from |___|___||___|___||___|___| to |___|___||___|___||___|___| 3) from |___|___||___|___||___|___| to |___|___||___|___||___|___|
Was the patient admitted to an Intensive Care Unit <u>for any cause</u> since the last visit?	❑ Yes<br>❑ No	If YES, specify reason(s) for admission(s) to ICU:<br>____________________________<br>If YES, please indicate date(s) of admission(s) to ICU since last visit (from DD/MM/YY to DD/MM/YY):<br>1) from |___|___||___|___||___|___| to |___|___||___|___||___|___| 2) from |___|___||___|___||___|___| to |___|___||___|___||___|___| 3) from |___|___||___|___||___|___| to |___|___||___|___||___|___|

TELEPHONE CONTACT – Day 120

Visit date |___|___||___|___||___|___| at |___|___| |___|___|
D D M M Y Y HOUR MINUTES

OCCURRING EVENTS (continued)

Did the patient have any episode(s) of <u>pulmonary embolism</u> since the last visit?	❑ Yes<br>❑ No	If YES indicate date(s) of onset of episode(s) of pulmonary embolism since last visit (DD/MM/YY):<br>1) |___|___||___|___||___|___| 2) |___|___||___|___||___|___| 3) |___|___||___|___||___|___|
Did the patient have any <u>cardiovascular events, including acute onset pulmonary edema</u> since the last visit?	❑ Yes<br>❑ No	If YES, specify kind of cardiovascular event(s):<br>________________________<br>If YES indicate date(s) of onset of new event(s) since last visit (DD/MM/YY):<br>1) |___|___||___|___||___|___| 2) |___|___||___|___||___|___| 3) |___|___||___|___||___|___|
Did the patient have any episodes of <u>pneumonia</u> since the last visit?	❑ Yes<br>❑ No	If YES indicate date(s) of onset of pneumonia since last visit (DD/MM/YY):<br>1) |___|___||___|___||___|___| 2) |___|___||___|___||___|___| 3) |___|___||___|___||___|___|
Did the patient have <u>any other significant occurring event</u> since the last visit?	❑ Yes<br>❑ No	If YES, specify event(s):<br>___________________________<br>If YES indicate date(s) of onset of new event(s) since last visit (DD/MM/YY):<br>1) |___|___||___|___||___|___| 2) |___|___||___|___||___|___| 3) |___|___||___|___||___|___|

Has the patient experienced <u>at least one</u> of the above events <u>since the last visit</u> (i.e. did you answer YES to any of the above questions)?

No ❑ Yes* ❑

* If YES please fax a copy of pages 64-65 to CROM as indicated in the Study Procedures Handbook.

TELEPHONE CONTACT – Day 120

Visit date |___|___||___|___||___|___| at |___|___| |___|___|
D D M M Y Y HOUR MINUTES

MEDICATION SINCE THE LAST VISIT

Has any medication been instituted, stopped and/or modified (dose change) since the last visit? ❑ No ❑ Yes

If YES please complete the following details, including only medications **instituted, stopped and/or modified (dose change)** *since the last visit.*

Trade name	Indication	Posology		Route	Administration (DD/MM/YY)	
		Dose/day	Unit		Start date	End date (or ONGOING)

PHARMACOECONOMIC QUESTIONNAIRE

Please remind to fill in the pharmacoeconomic questionnaire and enclose it in the plastic envelope.

INVESTIGATOR’S STATEMENT

I confirm that the data herein is a true complete and accurate reflection of this subject participation in the study, according to the protocol procedures.

Investigator’s name: ……………………………………………………………………………

Investigator’s signature: ……………………………………………………………………………

Date: |___|___||___|___||___|___|
D D M M Y Y

Centre No. |___|___|___| Screen. No. |___|___|___| Randomisation No. |___|___|___| Pat. Init. |___|___| |___|___|___|
Family name Name

TELEPHONE CONTACT – Day 150

Visit date |___|___||___|___||___|___| at |___|___| |___|___|
D D M M Y Y HOUR MINUTES

CLINICAL STATUS

Collect information on	Status	Comments: if "abnormal" specify:
1. Cardiovascular system	❑ Abnormal<br>❑ Normal<br>❑ Not done	
2. Respiratory system (except COPD)	❑ Abnormal<br>❑ Normal<br>❑ Not done	
3. Lymphatic system	❑ Abnormal<br>❑ Normal<br>❑ Not done	
4. Neurological-locomotor system	❑ Abnormal<br>❑ Normal<br>❑ Not done	
5. Skin	❑ Abnormal<br>❑ Normal<br>❑ Not done	
6. Endocrine	❑ Abnormal<br>❑ Normal<br>❑ Not done	
7. Ear-nose-throat system	❑ Abnormal<br>❑ Normal<br>❑ Not done	
8. Ophtalmological system	❑ Abnormal<br>❑ Normal<br>❑ Not done	
9. Gastrointestinal system	❑ Abnormal<br>❑ Normal<br>❑ Not done	
10. Others ……………………..	❑ Abnormal<br>❑ Normal<br>❑ Not done	

Centre No. |___|___|___| Screen. No. |___|___|___| Randomisation No. |___|___|___| Pat. Init. |___|___| |___|___|___|
Family name Name

TELEPHONE CONTACT – Day 150

Visit date |___|___||___|___||___|___| at |___|___| |___|___|
D D M M Y Y HOUR MINUTES

OCCURRING EVENTS

PLEASE COMPLETE THE FOLLOWING TABLE:

Did the patient have at least one COPD exacerbation* since the last visit?<br>(*defined as:<br>- worsening of respiratory symptoms requiring treatment with antibiotics and/or systemic steroids since last visit<br>- and/or new hospital admission for a COPD exacerbation since last visit<br>- and/or need for ventilatory support or intubation, or death since last visit)	❑ Yes<br>❑ No	If YES indicate date(s) of onset of new COPD exacerbation(s) since last visit (DD/MM/YY):<br>1) |___|___||___|___||___|___| 2) |___|___||___|___||___|___| 3) |___|___||___|___||___|___|
Was the patient hospitalized for a new COPD exacerbation since the last visit?	❑ Yes<br>❑ No	If YES indicate date(s) of new hospitalization(s) for COPD exacerbation(s) since last visit (from DD/MM/YY to DD/MM/YY):<br>1) from |___|___||___|___||___|___| to |___|___||___|___||___|___| 2) from |___|___||___|___||___|___| to |___|___||___|___||___|___| 3) from |___|___||___|___||___|___| to |___|___||___|___||___|___|
Was the patient hospitalized for any other cause (except COPD) since the last visit?	❑ Yes<br>❑ No	If YES, specify reason(s) for hospitalization(s):<br>___________________________<br>If YES, please indicate date(s) of new hospitalization(s) since last visit (from DD/MM/YY to DD/MM/YY):<br>1) from |___|___||___|___||___|___| to |___|___||___|___||___|___| 2) from |___|___||___|___||___|___| to |___|___||___|___||___|___| 3) from |___|___||___|___||___|___| to |___|___||___|___||___|___|
Was the patient admitted to an Intensive Care Unit for any cause since the last visit?	❑ Yes<br>❑ No	If YES, specify reason(s) for admission(s) to ICU:<br>___________________________<br>If YES, please indicate date(s) of admission(s) to ICU since last visit (from DD/MM/YY to DD/MM/YY):<br>1) from |___|___||___|___||___|___| to |___|___||___|___||___|___| 2) from |___|___||___|___||___|___| to |___|___||___|___||___|___| 3) from |___|___||___|___||___|___| to |___|___||___|___||___|___|

Centre No. |___|___|___| Screen. No. |___|___|___| Randomisation No. |___|___|___| Pat. Init. |___|___| |___|___|___|
Family name Name

TELEPHONE CONTACT – Day 150

Visit date |___|___||___|___||___|___| at |___|___| |___|___|
D D M M Y Y HOUR MINUTES

OCCURRING EVENTS (continued)

Did the patient have any episode(s) of pulmonary embolism since the last visit?	❑ Yes<br>❑ No	If YES indicate date(s) of onset of episode(s) of pulmonary embolism since last visit (DD/MM/YY):<br>1) |___|___||___|___||___|___| 2) |___|___||___|___||___|___| 3) |___|___||___|___||___|___|
Did the patient have any cardiovascular events, including acute onset pulmonary edema since the last visit?	❑ Yes<br>❑ No	If YES, specify kind of cardiovascular event(s):<br>______________________<br>If YES indicate date(s) of onset of new event(s) since last visit (DD/MM/YY):<br>1) |___|___||___|___||___|___| 2) |___|___||___|___||___|___| 3) |___|___||___|___||___|___|
Did the patient have any episodes of pneumonia since the last visit?	❑ Yes<br>❑ No	If YES indicate date(s) of onset of pneumonia since last visit (DD/MM/YY):<br>1) |___|___||___|___||___|___| 2) |___|___||___|___||___|___| 3) |___|___||___|___||___|___|
Did the patient have any other significant occurring event since the last visit?	❑ Yes<br>❑ No	If YES, specify event(s):<br>________________________<br>If YES indicate date(s) of onset of new event(s) since last visit (DD/MM/YY):<br>1) |___|___||___|___||___|___| 2) |___|___||___|___||___|___| 3) |___|___||___|___||___|___|

Has the patient experienced at least one of the above events since the last visit (i.e. did you answer YES to any of the above questions)?

No ❑ Yes* ❑

* If YES please fax a copy of pages 68-69 to CROM as indicated in the Study Procedures Handbook.

Centre No. |___|___|___| Screen. No. |___|___|___| Randomisation No. |___|___|___| Pat. Init. |___|___| |___|___|___|
Family name Name

TELEPHONE CONTACT – Day 150

Visit date |___|___||___|___||___|___| at |___|___| |___|___|
D D M M Y Y HOUR MINUTES

MEDICATION SINCE THE LAST VISIT

Has any medication been instituted, stopped and/or modified (dose change) since the last visit? ❑ No ❑ Yes

If YES please complete the following details, including only medications **instituted, stopped and/or modified (dose change)** *since the last visit.*

**Trade name**	**Indication**	**Posology**		**Route**	**Administration (DD/MM/YY)**	
		Dose/day	Unit		Start date	End date (or ONGOING)

PHARMACOECONOMIC QUESTIONNAIRE

Please remind to fill in the pharmacoeconomic questionnaire and enclose it in the plastic envelope.

INVESTIGATOR'S STATEMENT

I confirm that the data herein is a true complete and accurate reflection of this subject participation in the study, according to the protocol procedures.

Investigator's name: ……………………………………………………………………………

Investigator's signature: ……………………………………………………………………………

Date: |___|___||___|___||___|___|
D D M M Y Y

Centre No. |___|___|___| Screen. No. |___|___|___| Randomisation No. |___|___|___| Pat. Init. |___|___| |___|___|___|
Family name Name

VISIT 7 – Day 180

Visit date |___|___||___|___||___|___| at |___|___| |___|___|
D D M M Y Y HOUR MINUTES

Has the patient withdrawn prematurely from the study?

- ❑ Yes
- ❑ No

If Yes, please fill in the **"Investigator's statement"** at the end of visit 7 and the **"Study Conclusion Summary/Wihtdrawal/Drop-out Form"**.

Centre No. |___|___|___| Screen. No. |___|___|___| Randomisation No. |___|___|___| Pat. Init. |___|___| |___|___|___|
Family name Name

VISIT 7 – Day 180

Visit date |___|___||___|___||___|___| at |___|___| |___|___|
D D M M Y Y HOUR MINUTES

OCCURRING EVENTS

PLEASE COMPLETE THE FOLLOWING TABLE:

Did the patient have at least one COPD exacerbation* since the last visit?<br>(*defined as:<br>- worsening of respiratory symptoms requiring treatment with antibiotics and/or systemic steroids since last visit<br>- and/or new hospital admission for a COPD exacerbation since last visit<br>- and/or need for ventilatory support or intubation, or death since last visit)	❑ Yes<br>❑ No	If YES indicate date(s) of onset of new COPD exacerbation(s) since last visit (DD/MM/YY):<br>1) |___|___||___|___||___|___| 2) |___|___||___|___||___|___| 3) |___|___||___|___||___|___|
Was the patient hospitalized <u>for a new COPD exacerbation</u> since the last visit?	❑ Yes<br>❑ No	If YES indicate date(s) of new hospitalization(s) for COPD exacerbation(s) since last visit (from DD/MM/YY to DD/MM/YY):<br>1) from |___|___||___|___||___|___| to |___|___||___|___||___|___| 2) from |___|___||___|___||___|___| to |___|___||___|___||___|___| 3) from |___|___||___|___||___|___| to |___|___||___|___||___|___|
Was the patient hospitalized <u>for any other cause</u> (except COPD) since the last visit?	❑ Yes<br>❑ No	If YES, specify reason(s) for hospitalization(s):<br>____________________________<br>If YES, please indicate date(s) of new hospitalization(s) since last visit (from DD/MM/YY to DD/MM/YY):<br>1) from |___|___||___|___||___|___| to |___|___||___|___||___|___| 2) from |___|___||___|___||___|___| to |___|___||___|___||___|___| 3) from |___|___||___|___||___|___| to |___|___||___|___||___|___|
Was the patient admitted to an Intensive Care Unit <u>for any cause</u> since the last visit?	❑ Yes<br>❑ No	If YES, specify reason(s) for admission(s) to ICU:<br>____________________________<br>If YES, please indicate date(s) of admission(s) to ICU since last visit (from DD/MM/YY to DD/MM/YY):<br>1) from |___|___||___|___||___|___| to |___|___||___|___||___|___| 2) from |___|___||___|___||___|___| to |___|___||___|___||___|___| 3) from |___|___||___|___||___|___| to |___|___||___|___||___|___|

VISIT 7 – Day 180

Visit date |___|___||___|___||___|___| at |___|___| |___|___|
D D M M Y Y HOUR MINUTES

OCCURRING EVENTS (continued)

Did the patient have any episode(s) of pulmonary embolism since the last visit?	❑ Yes<br>❑ No	If YES indicate date(s) of onset of episode(s) of pulmonary embolism since last visit (DD/MM/YY):<br>1) |___|___||___|___||___|___| 2) |___|___||___|___||___|___| 3) |___|___||___|___||___|___|
Did the patient have any cardiovascular events, including acute onset pulmonary edema since the last visit?	❑ Yes<br>❑ No	If YES, specify kind of cardiovascular event(s):<br>_________________________<br>If YES indicate date(s) of onset of new event(s) since last visit (DD/MM/YY):<br>1) |___|___||___|___||___|___| 2) |___|___||___|___||___|___| 3) |___|___||___|___||___|___|
Did the patient have any episodes of pneumonia since the last visit?	❑ Yes<br>❑ No	If YES indicate date(s) of onset of pneumonia since last visit (DD/MM/YY):<br>1) |___|___||___|___||___|___| 2) |___|___||___|___||___|___| 3) |___|___||___|___||___|___|
Did the patient have any other significant occurring event since the last visit?	❑ Yes<br>❑ No	If YES, specify event(s):<br>____________________________<br>If YES indicate date(s) of onset of new event(s) since last visit (DD/MM/YY):<br>1) |___|___||___|___||___|___| 2) |___|___||___|___||___|___| 3) |___|___||___|___||___|___|

Has the patient experienced at least one of the above events since the last visit (i.e. did you answer YES to any of the above questions)?

No ❑ Yes* ❑

* If YES please fax a copy of pages 72-73 to CROM as indicated in the Study Procedures Handbook.

Centre No. |___|___|___| Screen. No. |___|___|___| Randomisation No. |___|___|___| Pat. Init. |___|___| |___|___|___|
Family name Name

VISIT 7 – Day 180

Visit date |___|___||___|___||___|___| at |___|___| |___|___|
D D M M Y Y HOUR MINUTES

MEDICATION SINCE THE LAST VISIT

Has any medication been instituted, stopped and/or modified (dose change) since the last visit? ❑ No ❑ Yes

If YES please complete the following details, including only medications **instituted, stopped and/or modified (dose change)** *since the last visit.*

Trade name	Indication	Posology		Route	Administration (DD/MM/YY)	
		Dose/day	Unit		Start date	End date (or ONGOING)

VISIT 7 – Day 180

Visit date |___|___||___|___||___|___| at |___|___| |___|___|
D D M M Y Y HOUR MINUTES

PHYSICAL EXAMINATION

Physical examination	Status	Comments: if "abnormal" specify:
Cardiovascular system	❑ Abnormal<br>❑ Normal<br>❑ Not done	
Respiratory system<br>(except COPD)	❑ Abnormal<br>❑ Normal<br>❑ Not done	
Lymphatic system	❑ Abnormal<br>❑ Normal<br>❑ Not done	
Neurological-locomotor system	❑ Abnormal<br>❑ Normal<br>❑ Not done	
Skin	❑ Abnormal<br>❑ Normal<br>❑ Not done	
Endocrine	❑ Abnormal<br>❑ Normal<br>❑ Not done	
Ear-nose-throat system	❑ Abnormal<br>❑ Normal<br>❑ Not done	
Ophtalmological system	❑ Abnormal<br>❑ Normal<br>❑ Not done	
Gastrointestinal system	❑ Abnormal<br>❑ Normal<br>❑ Not done	
Others<br>……………………..	❑ Abnormal<br>❑ Normal<br>❑ Not done	

VITAL SIGNS

SITTING BLOOD PRESSURE (mmHg)		SITTING HEART RATE (bpm)
Systolic	Diastolic	
|___|___|___|	|___|___|___|	|___|___|___|

Centre No. |___|___|___| Screen. No. |___|___|___| Randomisation No. |___|___|___| Pat. Init. |___|___| |___|___|___|
Family name Name

VISIT 7 – Day 180

Visit date |___|___||___|___||___|___| at |___|___| |___|___|
D D M M Y Y HOUR MINUTES

Pro-calcitonin (pro-ct)

Has the blood sample been taken for the pro-ct analysis?

Yes ❑ No ❑

If Yes, refer to the Study Procedures Handbook for handling and storage.

If No, please specify why: ____

Please fill in as soon as the results are available:

Pro-calcitonin (mcg/L) I___I___I.I___I___I

C-reactive protein (CRP), serum amyloid (SAA) and RANTES

Has the blood sample been taken for the CRP, SAA and RANTES analysis?

Yes ❑ No ❑

If Yes, refer to the Study Procedures Handbook for handling and storage.

If No, please specify why: ____

Please fill in as soon as the results are available:

CRP (mg/L) I___I___I.I___I___I

SAA (mg/L) I___I___I.I___I___I

RANTES (mcg/L) I___I___I.I___I___I

Centre No. |___|___|___| Screen. No. |___|___|___| Randomisation No. |___|___|___| Pat. Init. |___|___| |___|___|___|
Family name Name

VISIT 7 – Day 180

Visit date |___|___||___|___||___|___| at |___|___| |___|___|
D D M M Y Y HOUR MINUTES

QUESTIONNAIRES FOR RESPIRATORY SYMPTOMS/QUALITY OF LIFE

SF36 score: total |___|___|___|

BDI score: total |___|___|___|

TDI score: total |___|___|___|

CCIQ Score: total |___|___|___|

PHARMACOECONOMIC QUESTIONNAIRE

Please remind to fill in the pharmacoeconomic questionnaire and enclose it in the plastic envelope.

SPIROMETRY

- **For this visit, please obtain a complete spirometry.**
- **SPIROMETRY:** from 3 acceptable spirometric manoeuvres record the highest FEV_1 and FVC, irrespective of the manoeuvre (curve) they come from.

date |___|___| |___|___| |___|___| at |__|__| |__|__|
HOUR MINUTES

**FVC**	|___|, |___|___|___| litre
**FEV_1**	|___|, |___|___|___| litre
**FEV_1 predicted normal value**	|___|, |___|___|___| litre [ERS standards]
**FEV_1 (% predicted normal value)**	|___|___| % of predicted value
**FEV_1/FVC (%)**	|___|___|___|, |___| %
**RV**	|___|, |___|___|___| litre
**RV (% predicted normal value)**	|___|___|___|, |___| %
**TLC**	|___|, |___|___|___| litre
**TLC (% predicted normal value)**	|___|___|___|, |___| %
**RV/TLC (%)**	|___|___|___|, |___| %
**KCO**	|___|___|, |___||___| ml/min/mmHg/l
**KCO (% predicted normal value)**	|___|___|___|, |___| %

VISIT 7 – Day 180

Visit date |___|___||___|___||___|___| at |___|___| |___|___|
D D M M Y Y HOUR MINUTES

MICROBIOLOGY

	Yes	No
Sputum collection performed:	❑	❑

If YES, specify volume of sputum collected: |___|,|___| ml

If NO, specify why ____

Results from local laboratory:

Bacteriology:

Identified microorganism #1: ____________________________ CFU/ml: |___|___|$^{|\_\_|\_\_|}$

Identified microorganism #2: ____________________________ CFU/ml: |___|___|$^{|\_\_|\_\_|}$

Identified microorganism #3: ____________________________ CFU/ml: |___|___|$^{|\_\_|\_\_|}$

Serology for Intracellular pathogens:

Has the sample for serology for intracellular pathogens been obtained? Yes ❑ No ❑

If Yes, please complete below:

Was serology positive for **Mycoplasma pneumoniae**? Yes ❑ No ❑

If YES, indicate: IgM: |___|___|,|___|___|

IgG: |___|___|,|___|___|

Was serology positive for **Chlamydia**? Yes ❑ No ❑

If YES, indicate: IgM: |___|___|,|___|___|

IgG: |___|___|,|___|___|

Was serology positive for **Legionella pneumophila**? Yes ❑ No ❑

If YES, indicate:

	**POSITIVE**	**NEGATIVE**
Legionella pneumophila IgM	❑	❑
Legionella pneumophila IgG	❑	❑

VISIT 7 – Day 180

Visit date |___|___||___|___||___|___| at |___|___| |___|___|
D D M M Y Y HOUR MINUTES

MICROBIOLOGY (continued)

Has the sample for **respiratory viruses RT-PCR analysis** been collected and stored at –80°C?

Yes ❑ No ❑

If **YES,** specify volume stored at -80°C for RT-PCR: |___|,|___||___||___| ml

Please remember to label the sample with the patient's ID number and store it at -80°C, as described in the Study Procedures Handbook.

Has the sample for **inflammatory cell counts** been collected? Yes ❑ No ❑

Were the slides prepared and stored at -20°C? Yes ❑ No ❑

If YES: how many slides were prepared? |___|___|

VISIT 7 – Day 180

Visit date |___|___||___|___||___|___| at |___|___| |___|___|
D D M M Y Y HOUR MINUTES

ARTERIAL BLOOD GASES

	Yes	No
Arterial blood gas analysis performed:	❑	❑
**If YES**: patient was under O_2 therapy?	❑	❑

If patient was under O_2 therapy please indicate:

- how many L/min: |___|,|___|
- how many hours/day: |___|___|

If arterial blood gas analysis was performed, indicate:

pH: |___|,|___|___|

PaO_2: |___|___|___| mmHg

$PaCO_2$: |___|___|___| mmHg

SO_2: |___|___|___| %

HCO_3^-: |___|___|___| mmol/L

INVESTIGATOR'S STATEMENT

I confirm that the data herein is a true complete and accurate reflection of this subject participation in the study, according to the protocol procedures.

Investigator's name: …………………………………………………………………………

Investigator's signature: ……………………………………………………………………

Date: |___|___||___|___||___|___|
D D M M Y Y

Centre No. |___|___|___| Screening No. |___|___|___| Randomisation No. |___|___|___| Visit No. |___|___| Patient's Initials |___|___| |___|___|___|
Family name Name

ADVERSE EVENT(S) REPORT FORM

SECTION A

ADVERSE EVENT, SPECIFY	SERIOUS	REASON (several statements are possible)	DATE OF ONSET	DATE OF END	SEVERITY	RELATIONSHIP	ACTION TAKEN (several statements are possible)	OUTCOME OF EVENT
Please, list ONE event per line	1. No<br>2. Yes	1. Fatal<br>2. Life-threatening<br>3. Inpatient hospitalisation or prolonged inpatient hospitalisation<br>4. Permanent or significant disability / Incapacity<br>5. Congenital anomaly / Birth defect<br>6. May require medical intervention to prevent the above outcomes	(dd mm yy)	(dd mm yy)	1. Mild<br>2. Moderate<br>3. Severe	1. Certain/Definite<br>2. Probable/Likely<br>3. Possible<br>4. Doubtful/Unlikely<br>5. Not available<br>6. Not assessable<br>7. Not related	1. None<br>2. Drug treatment<br>3. Discontinuation of study drug(s)<br>4. Discontinuation of concomitant drug(s)	1. Recovered<br>2. Recovered with sequelae<br>3. Not yet recovered<br>4. Death: drug may be contributory<br>5. Death: due to adverse event<br>6. Death: unrelated to drug<br>7. Unknown
1.	1 ☐ 2 ☐	1 ☐ 2 ☐ 3 ☐ 4 ☐ 5 ☐ 6 ☐	____/___/____	____/___/____	1 ☐ 2 ☐ 3 ☐	1 ☐ 2 ☐ 3 ☐ 4 ☐ 5 ☐ 6 ☐ 7 ☐	1 ☐ 2 ☐ 3 ☐ 4 ☐	1 ☐ 2 ☐ 3 ☐ 4 ☐ 5 ☐ 6 ☐ 7 ☐
2.	1 ☐ 2 ☐	1 ☐ 2 ☐ 3 ☐ 4 ☐ 5 ☐ 6 ☐	____/___/____	____/___/____	1 ☐ 2 ☐ 3 ☐	1 ☐ 2 ☐ 3 ☐ 4 ☐ 5 ☐ 6 ☐ 7 ☐	1 ☐ 2 ☐ 3 ☐ 4 ☐	1 ☐ 2 ☐ 3 ☐ 4 ☐ 5 ☐ 6 ☐ 7 ☐
3.	1 ☐ 2 ☐	1 ☐ 2 ☐ 3 ☐ 4 ☐ 5 ☐ 6 ☐	____/___/____	____/___/____	1 ☐ 2 ☐ 3 ☐	1 ☐ 2 ☐ 3 ☐ 4 ☐ 5 ☐ 6 ☐ 7 ☐	1 ☐ 2 ☐ 3 ☐ 4 ☐	1 ☐ 2 ☐ 3 ☐ 4 ☐ 5 ☐ 6 ☐ 7 ☐
4.	1 ☐ 2 ☐	1 ☐ 2 ☐ 3 ☐ 4 ☐ 5 ☐ 6 ☐	____/___/____	____/___/____	1 ☐ 2 ☐ 3 ☐	1 ☐ 2 ☐ 3 ☐ 4 ☐ 5 ☐ 6 ☐ 7 ☐	1 ☐ 2 ☐ 3 ☐ 4 ☐	1 ☐ 2 ☐ 3 ☐ 4 ☐ 5 ☐ 6 ☐ 7 ☐
5.	1 ☐ 2 ☐	1 ☐ 2 ☐ 3 ☐ 4 ☐ 5 ☐ 6 ☐	____/___/____	____/___/____	1 ☐ 2 ☐ 3 ☐	1 ☐ 2 ☐ 3 ☐ 4 ☐ 5 ☐ 6 ☐ 7 ☐	1 ☐ 2 ☐ 3 ☐ 4 ☐	1 ☐ 2 ☐ 3 ☐ 4 ☐ 5 ☐ 6 ☐ 7 ☐

FILL IN THE REASON FOR SERIOUSNESS AND THE COMPLEMENTARY PAGE FOR SERIOUS ADVERSE EVENTS AND REPORT BOTH PAGES TO *CROM AND THE DIREZIONE SANITARIA OF THE HOSPITAL* WITHIN A WORKING DAY (SEE STUDY PROCEDURES HANDBOOK FOR INSTRUCTIONS).

Date:____/____/_____ Investigator's Signature:_________________

Centre No. |__|__|__| Screening No. |__|__|__| Randomisation No. |__|__|__| Visit No. |__|__| Patient's Initials |__|__| |__|__|__|
Family name Name

COMPLEMENTARY PAGE FOR THE SERIOUS ADVERSE EVENT(S)

SECTION B

FURTHER DETAILS OF ADVERSE EVENT

**REPORT TYPE**	**CODE BROKEN**	**COUNTRY WHERE THE ADVERSE EVENT OCCURRED**	**DATE OF BIRTH (**dd/mm/yy)	**AGE** (years)	**SEX**
Initial ☐ Follow-up ☐	Yes ☐ No ☐				Male ☐ Female ☐

**CONCOMITANT DISEASE(S)**	**MEDICAL HISTORY**

**SUSPECTED DRUG(S)**	Indication for use	Daily dose	Route of administration	Treatment dates (dd/mm/yyyy)	Comments
				From ____/____/_____ To ____/____/_____	
				From ____/____/_____ To ____/____/_____	

**DECHALLENGE AND RECHALLENGE**	Did suspect study drug (s) discontinue?	Did reaction abate after stopping study drug(s)?	Was/were study drug(s) readministered?	Did reaction reappear after reintroduction?
	Yes ☐ No ☐ NA ☐	Yes ☐ No ☐ NA ☐	Yes ☐ No ☐ NA ☐	Yes ☐ No ☐ NA ☐

**CONCOMITANT MEDICATION(S)** (Tradename/Generic name)	Indication for use	Daily dose	Route of administration	Treatment dates (dd/mm/yyyy)	**Comments**
				From ____/____/_____ To ____/____/_____	
				From ____/____/_____ To ____/____/_____	

**POSSIBLE EXPLANATION(S) FOR SERIOUS ADVERSE EVENT**	**IN CASE OF OUTCOME "DEATH"** attach copy of hospital report, autopsy report, death certificate, if available)**:**
☐ Underlying disease ☐ Intercurrent disease ☐ Study drug<br>☐ Concomitant disease ☐ Concomitant drug	Date of death (dd/mm/yy): ____/____/_____<br>☐ Natural course of disease ☐ Other, please specify:<br>____

Date:____/____/_____ Investigator's Signature:_________________

Centre No. |___|___|___| Screening No. |___|___|___| Randomisation No. |___|___|___| Visit No. |___|___| Patient's Initials |___|___| |___|___|___|
Family name Name

COMPLEMENTARY PAGE FOR THE SERIOUS ADVERSE EVENT(S)

SECTION B

FURTHER DETAILS OF ADVERSE EVENT

**REPORT TYPE**	**CODE BROKEN**	**COUNTRY WHERE THE ADVERSE EVENT OCCURRED**	**DATE OF BIRTH** (dd/mm/yy)	**AGE** (years)	**SEX**
Initial ☐ Follow-up ☐	Yes ☐ No ☐				Male ☐ Female ☐

**CONCOMITANT DISEASE(S)**	**MEDICAL HISTORY**

**SUSPECTED DRUG(S)**	Indication for use	Daily dose	Route of administration	Treatment dates (dd/mm/yyyy)	Comments
				From ____/____/_____ To ____/____/_____	
				From ____/____/_____ To ____/____/_____	

**DECHALLENGE AND RECHALLENGE**	Did suspect study drug (s) discontinue?	Did reaction abate after stopping study drug(s)?	Was/were study drug(s) readministered?	Did reaction reappear after reintroduction?
	Yes ☐ No ☐ NA ☐	Yes ☐ No ☐ NA ☐	Yes ☐ No ☐ NA ☐	Yes ☐ No ☐ NA ☐

**CONCOMITANT MEDICATION(S)** (Tradename/Generic name)	Indication for use	Daily dose	Route of administration	Treatment dates (dd/mm/yyyy)	**Comments**
				From ____/____/_____ To ____/____/_____	
				From ____/____/_____ To ____/____/_____	

POSSIBLE EXPLANATION(S) FOR SERIOUS ADVERSE EVENT
☐ Underlying disease ☐ Intercurrent disease ☐ Study drug
☐ Concomitant disease ☐ Concomitant drug

IN CASE OF OUTCOME "DEATH" attach copy of hospital report, autopsy report, death certificate, if available)**:**
Date of death (dd/mm/yy): ____/____/_____
☐ Natural course of disease ☐ Other, please specify:
____

Date:____/____/_____ Investigator's Signature:_________________

Centre No. |___|___|___| Screening No. |___|___|___| Randomisation No. |___|___|___| Visit No. |___|___| Patient's Initials |___|___| |___|___|___|
Family name Name

COMPLEMENTARY PAGE FOR THE SERIOUS ADVERSE EVENT(S)

SECTION B

FURTHER DETAILS OF ADVERSE EVENT

**REPORT TYPE**	**CODE BROKEN**	**COUNTRY WHERE THE ADVERSE EVENT OCCURRED**	**DATE OF BIRTH (**dd/mm/yy)	**AGE** (years)	**SEX**
Initial ☐ Follow-up ☐	Yes ☐ No ☐				Male ☐ Female ☐

**CONCOMITANT DISEASE(S)**	**MEDICAL HISTORY**

**SUSPECTED DRUG(S)**	Indication for use	Daily dose	Route of administration	Treatment dates (dd/mm/yyyy)	Comments
				From ____/____/_____ To ____/____/_____	
				From ____/____/_____ To ____/____/_____	

**DECHALLENGE AND RECHALLENGE**	Did suspect study drug (s) discontinue?	Did reaction abate after stopping study drug(s)?	Was/were study drug(s) readministered?	Did reaction reappear after reintroduction?
	Yes ☐ No ☐ NA ☐	Yes ☐ No ☐ NA ☐	Yes ☐ No ☐ NA ☐	Yes ☐ No ☐ NA ☐

**CONCOMITANT MEDICATION(S)** (Tradename/Generic name)	Indication for use	Daily dose	Route of administration	Treatment dates (dd/mm/yyyy)	**Comments**
				From ____/____/_____ To ____/____/_____	
				From ____/____/_____ To ____/____/_____	

**POSSIBLE EXPLANATION(S) FOR SERIOUS ADVERSE EVENT**	**IN CASE OF OUTCOME "DEATH"** attach copy of hospital report, autopsy report, death certificate, if available)**:**
☐ Underlying disease ☐ Intercurrent disease ☐ Study drug<br>☐ Concomitant disease ☐ Concomitant drug	Date of death (dd/mm/yy): ____/____/_____<br>☐ Natural course of disease ☐ Other, please specify:<br>____

Date:____/____/_____

Investigator's Signature:_________________

STUDY CONCLUSION SUMMARY/WITHDRAWAL/DROP-OUT FORM

Did the patient complete the study? ❑ Yes ❑ No

Date of last study treatment (antibiotic) intake: |___|___||___|___||___|___|
D D M M Y Y

If Yes: Date of study completion: |___|___||___|___||___|___|
D D M M Y Y

If No: - Date of withdrawal: |___|___||___|___||___|___|
D D M M Y Y

- Reason for the withdrawal:

- Did not satisfy all inclusion/exclusion criteria ❑
  Specify: ………………………………………………………………………………
- Protocol violation ❑
  Specify: ……………………………………………………………………………....
- Adverse event (noted on adverse event page) ❑
- Death ❑
  Specify cause of death: ………………………………………………………………..
- Withdrawal of consent ❑
- Pregnancy ❑
- Insufficient therapeutic response ❑
- Lost to follow-up visit ❑
- Poor compliance ❑
- Other reason(s) ❑
  Specify: ……..…………………………………………………………………...

INVESTIGATOR'S STATEMENT

I confirm that the data herein is a true complete and accurate reflection of this subject participation in the study, according to the protocol procedures.

Investigator's name: ………………………………………………………………………………

Investigator's signature: ………………………………………………………………………………

Date: |___|___||___|___||___|___|
D D M M Y Y
