# Supplementary material for: Antibiotic Treatment of Severe Exacerbations of Chronic Obstructive Pulmonary Disease with Procalcitonin: A Randomized Noninferiority Trial
Source: PLoS One. 2015 Mar 11;10(3):e0118241. doi: 10.1371/journal.pone.0118241 (PMC4356612; doi:10.1371/journal.pone.0118241)
Supplement: S1 Supporting Information — (PDF) [file pone.0118241.s005.pdf]

Mod. 2384

Regione Puglia
OSPEDALI RIUNITI
Azienda Ospedaliero - Universitaria
FOGGIA

Deliberazione del Direttore Generale

Nominato con Delibera di Giunta Regionale n.1251 del 6.9.2005

N. 1270 del Registro

Oggetto: Sperimentazione clinica prot. AIFA BPCO
Struttura di Mal. App. Resp. 4 - Sponsor Agenzia Italiana del Farmaco

L'anno 2006 giorno 8 del mese di NOVEMBRE, nella sede dell'Azienda Ospedaliera "Ospedali Riuniti", il Direttore Generale dott. Tommaso Moretti, con la partecipazione del Direttore Amministrativo e del Direttore Sanitario, e con l'assistenza del Segretario redigente sig.ra Filomena Filannino sulla base dell'istruttoria espletata dall'ufficio competente e su proposta del Dirigente responsabile, che attesta la legittimità e conformità della proposta alla vigente normativa, adotta il provvedimento che segue:

Visto che il Direttore di Struttura di Mal.App.Resp.4 di questa Azienda ha presentato istanza tesa ad ottenere l'autorizzazione a svolgere uno sperimentazione clinica dal titolo "Confronto fra un regime terapeutico basato su livelli sierici di procalcitonina rispetto a un regime terapeutico standard in pazientoi ospedalizzati per riacutizzazione di broncopatia cronica ostruttiva" Prot. AIFA BPCO

Considerato che lo studio in parola avrebbe una positiva ricaduta sull'arricchimento professionale degli operatori della Struttura richiedente;

Visto l'attestato del Comitato Etico prot.44/CE/06 del 25.10.06 con il quale viene approvato il protocollo di studio;

Vista la dichiarazione sottoscritta dagli interessati, ai sensi del regolamento aziendale sulle sperimentazioni approvato con deliberazione 1105/2005, secondo cui il 100% degli utili debba essere destinato a fondo della struttura di Mal. App. Resp.4

Ritenuto di dover autorizzare lo studio in oggetto, il quale prevede la corresponsione da parte dell'Agenzia Italiana del Farmaco tramite il Dipartimento Integrato di Oncologia e Ematologia dell'Università degli Studi di Modena e Reggio Emilia di un contributo di euro 10000,00 da considerarsi non assoggettabile a IVA.

Ritenuto che il 100% delle somme citate debbano essere destinata a fondo della Struttura di Mal.App.Resp. 4.

Acquisiti i pareri favorevoli dei Direttori Sanitario e Amministrativo.

DELIBERA

Grafiche DIGIMAIL - Foggia

Regione Puglia

OSPEDALI RIUNITI

Azienda Ospedaliero – Universitaria

FOGGIA

Prot.n. 44/CE/06

COMITATO ETICO

Tel.0881/2593 Fax 0881/732249 E.Mail m.cocomalud@tiscalinet.it

Oggetto: Approvazione sperimentazione

Il Comitato Etico dell'Azienda Ospedaliera "Ospedali Riuniti" nella riunione del 25.10.2006 dopo aver esaminato la documentazione prevista dal "Regolamento per l'esecuzione di sperimentazioni cliniche su farmaci, dispositivi medici e studi osservazionali nelle strutture dell'azienda" approva lo studio dal titolo ***"Confronto fra un regime terapeutico basato su livelli sierici di procalcitonina rispetto ad un regime terapeutico standard in pazienti ospedalizzati per riacutizzazione di broncopatia cronica ostruttiva" Prot. AIFA BPCO***

Documentazione esaminata: Protocollo 2.9.2006, Sinossi 2.9.06, Lettera medico curante
Foglio informativo e consenso informato, Questionario farmacoeconomia
Elenco centri, CRF, Parere centro coordinatore, Questionari SF-36, CCIQ, BDI e TDI

Sperimentatori: Giovanna Elisiana Carpagnano, Tiziana Giliberti, Annarita De Palo

Dott. Michele Monaco – Ematologo	~~Si~~ No [signature]
Prof. Raffele De Cesaris – Dir.Strutt. Medicina I Univ.	~~Si~~ No [signature]
Prof. Vittorio Fineschi – Dir. Strutt. Medicina Legale. Univ.	Si No ASSENTE
Dott. Romano Bucci – Resp. Reumatol. Osp.	Si No ASSENTE
Dott. Fausto Tricarico – Dir. Strutt. Chir.Gen.2 Osp.	Si No ASSENTE
Dott. Nicola Crucinio – Dirigente Medico Gastroenterologia	~~Si~~ No [signature]
Dott.ssa Daniela Pedà – Direttore Medico Pres.Biostatistica	~~Si~~ No [signature]
Dott. Giuseppe Gallo – Dirigente Med. DMP- Bioetica	~~Si~~ No [signature]
Dott.ssa Rosanna Stea – Direttore Farmacia	~~Si~~ No Rosanna Stea
Avv. Simona Mastropieri – Avvocato	~~Si~~ No [signature]
Prof.ssa Luigia Trabace – Farmacologo	Si No ASSENTE
Dott. Vitangelo Dattoli – Direttore Sanitario	Si No ASSENTE
Sig.ra Nadia Belgioioso – Caposala	~~Si~~ No [signature]
Ing. Maria Rosaria Castrignanò – TDM – Presidente	~~Si~~ No [signature]

Il Segretario
(Michele Cocomazzi)
Michele Cocom[signature]

AZIENDA OSPEDALIERA UNIVERSITARIA
"SAN MARTINO"
Largo Rosanna Benzi n. 10
16132 GENOVA

Verbale n. 02/2007

**VERBALE DELLA SEDUTA DEL COMITATO ETICO
DELL'AZIENDA OSPEDALIERA UNIVERSITARIA "SAN MARTINO"
DEL 09 FEBBRAIO 2007**

In data 09 febbraio 2007, alle ore 9,00 presso la Sala delle Ceramiche dell'Azienda Ospedaliera Universitaria "San Martino", si è riunito il Comitato Etico.

Sono presenti i seguenti Componenti Effettivi:

DOTT. LUIGI FRANCESCO MELONI (Presidente Comitato Etico, Magistrato); **PROF. FRANCO DALLEGRI** (Prof. Ordinario di Medicina Interna, Università di Genova – Direttore U.O. Clinica Medicina Interna 1, Azienda Ospedaliera Universitaria "San Martino", Genova); **PROF.SSA ANTONIETTA MARTELLI** (Prof. Ordinario di Farmacologia, Università di Genova); **PROF. GIANCARLO ICARDI** (Prof. Ordinario di Igiene Generale e applicata, Università di Genova – Direttore U.O. Igiene, Azienda Ospedaliera Universitaria "San Martino", Genova); **DOTT. LUCIANO LUSARDI** (Medico di Medicina Generale)a "San Martino" Genova)

Sono presenti i seguenti Componenti Supplenti:

DOTT.SSA BARBARA REBESCO (Dirigente, U.O.Farmacia, Azienda Ospedaliera Universitaria "San Martino", Genova - Componente Supplente della Dott.ssa Maria Elvira Amalfitano);**PROF. DOMENICO PALOMBO** (Direttore U.O. Chirurgia Vascolare ed Endovascolare, Azienda Ospedaliera Universitaria "San Martino", Genova – Componente Supplente del Prof. A. Sobrero)

Sono assenti giustificati:

DOTT.SSA MARIA ELVIRA AMALFITANO (Direttore U.O. Farmacia Azienda Ospedaliera Universitaria "San Martino", Genova); **SIG.RA MARIA CHICHINE MASSIDDA** (Presidente Confederazione dei Centri Liguri per la tutela dei diritti del malato); **PROF. RENZO CELESTI** (Prof. Ordinario di Medicina Legale con competenze di bioetica, Università di Genova – Direttore della U.O. Medicina Legale, Azienda Ospedaliera Universitaria "San Martino", Genova); **DOTT. FRANCESCO COPELLO** (Direttore Sanitario, Azienda Ospedaliera Universitaria San martino Genova); **PROF. ALBERTO SOBRERO** (Direttore della U.O. Oncologia Medica, Azienda Ospedaliera Universitaria "San Martino", Genova) **DOTT.SSA LAURA TIBALDI** (Collaboratore professioni sanitarie esperto con funzioni di coordinamento, Azienda Ospedaliera Universitaria "San Martino", Genova);

Alle ore 9.10 il Presidente, Dr. Luigi Francesco Meloni, constatata la presenza del numero legale, dichiara aperta la seduta del Comitato Etico.

Partecipa a questa seduta, senza diritto di voto, la Dott.ssa Maria Lucrezia Mazzarella, specializzanda in Medicina Legale.

.........*OMISSIS*.......

VALUTAZIONE PROGETTI DI RICERCA

Alle ore 9.25 entra il Dott. Fulvio Braido (U.O. Clinica Tisiologica e Pneumologica), in sostituzione del Prof. G.W. Canonica, il quale presenta il **punto 1** dell'o.d.g.:

"Confronto fra un regime terapeutico basato sui livelli sierici di procalcitonina rispetto ad un regime terapetucio standard in pazienti ospedalizzati per riacutizzazione di broncopneumopatia cronica ostruttiva"

Prot. FARM58J2XH (Reg. 161/2006)

Elenco documentazione: Lettera per via e-mail di autorizzazione del Prof. Canonica;Dati identificativi del protocollo;Parere Unico del centro coordinatore azienda Ospedaliera Universitaria di Modena +Versione emendata del 02.9.06;Chronic Cough Impact Questionnaire;Case report Form;Lista dei centri partecipanti;Scheda informativa per il paziente;Lettera per il medico curante;Lettera dell'Aifa;Modulo per il consenso informato;Study Protocol versione 24 luglio 06;Indice basale di dispnea;Schede di segnalazione;Indice basale di dispneia (BDI);Punteggi questionari BDI,TDI e MRC;Indice di dispnea di transizione(TDI);Questionario sullo stato di salute;Sinossi del protocollo;Convenzione economica;

Il Dott. Braido illustra il protocollo e viene congedato alle ore 9:30. Il Comitato, dopo breve discussione, approva la sperimentazione all'unanimità.

.........*OMISSIS*.......

La seduta è tolta alle ore 11.30.

E' copia conforme all'originale
Il Dirigente Amministrativo U.O.
Affari Generali e Legali

AZIENDA OSPEDALIERA UNIVERSITARIA
"SAN MARTINO"
Largo Rosanna Benzi n. 10
16132 GENOVA

Comitato Etico Aziendale
(Costituito Con Deliberazione n. 1075 Del 14/04/2005 - N. 11 Componenti)

Oggetto: registrazione presenze alla seduta CEA del 09/02/2007

Componenti Supplenti

PROF. ROBERTO BALESTRERI ....
(già Direttore Clinica Gerontologia, Università di Genova)
Ente di appartenenza: UNIVERSITA DEGLI STUDI DI GENOVA
[Componente Supplente del Prof. Franco Dallegri]

Dott. Angelo CANEPA ....
(Medico di Medicina Generale)
Ente di appartenenza: LIBERO PROFESSIONISTA convenzionato con S.S.N.
[Componente Supplente del Dott. Luciano Lusardi]

DOTT.SSA ALESSANDRA MORANDO ....
(Dirigente Medico, U.O. Direzione Sanitaria, Azienda Ospedaliera Universitaria "San Martino", Genova)
Ente di appartenenza: AZIENDA OSPEDALIERA UNIVERSITARIA SAN MARTINO DI GENOVA
[Componente Sostituto del Direttore Sanitario]

PROF. DOMENICO PALOMBO ....
(Direttore U.O. Chirurgia Vascolare ed Endovascolare, Azienda Ospedaliera Universitaria "San Martino", Genova)
Ente di appartenenza: AZIENDA OSPEDALIERA UNIVERSITRIA SAN MARTINO DI GENOVA
[Componente Supplente del Prof. Alberto Sobrero]

DOTT.SSA BARBARA REBESCO ....
(Dirigente U.O. Farmacia, Azienda Ospedaliera Universitaria "San Martino", Genova)
Ente di appartenenza: AZIENDA OSPEDALIERA UNIVERSITARIA SAN MARTINO DI GENOVA
[Componente Supplente della Dott.ssa Maria Elvira Amalfitano]

Sig.ra Marcella REMONDINI ROSSI ....
(Rappresentante AVO)
[Componente Supplente della Sig.ra Maria Chighine Massidda]

I sopra elencati componenti del Comitato Etico dichiarano che si asterranno dal pronunciarsi su quelle Sperimentazioni per le quali possa sussistere un conflitto di interessi di tipo diretto o indiretto.

ASL MilanoDue

Azienda Sanitaria Locale della Provincia di Milano 2

PROT. 1949 Spontaneo

IL COMITATO ETICO INDIPENDENTE

Istituito con Deliberazione n. 78 in data 14 gennaio 1999 e successivamente aggiornato e integrato, si è riunito in data **19 settembre 2006** alle ore **14.00** nei locali della A.S.L. Provincia di Milano 2 in via VIII Giugno n.69 in Melegnano per valutare la documentazione, pervenuta dall'Istituto Clinico Humanitas, inerente alla sperimentazione, avente per titolo:

Confronto fra un regime terapeutico basato sui livelli sierici di procalcitonina rispetto ad un regime terapeutico standard in pazienti ospedalizzati per riacutizzazione di broncopneumopatia cronica ostruttiva.

Documenti esaminati:

- Protocollo finale dello studio 24 luglio 2006;
- Modulo Consenso Informato;
- Foglio Informativo paziente;
- Lettera al MMG;
- Questionario BDI – TDI;
- CRF Draft 31 luglio 2006;
- CCIQ-AIFA 2006;
- Lista centri partecipanti;
- Questionario farmaco economia-AIFA 2006;
- SF36_AIFA2006;
- Sinossi Finale 24 luglio 2006;
- Questionario BDI punteggio
- Questionario BDI-TDI-MRC punteggi
- Dichiarazione Polizza Assicurativa Humanitas
- Questionario TDI punteggio.
- Dichiarazione di Garanzia del 5 settembre 2006

Responsabile dello Studio	Dr. Ciccarelli
Reparto coinvolto	Pneumologia

Presentatore dello studio Dr. Ciccarelli

PROT. 1949
Spontaneo

Dopo valutazione il Comitato esprime

- [x] **PARERE FAVOREVOLE**
- [ ] **PARERE NON FAVOREVOLE IN QUANTO**
- [ ] **PARERE SOSPESO**
- [ ] **PARERE FAVOREVOLE CON RISERVA:**

....

....

....

I Componenti che il giorno **19 settembre 2006** sono espressi in merito alla sperimentazione avente per titolo:

Confronto fra un regime terapeutico basato sui livelli sierici di procalcitonina rispetto ad un regime terapeutico standard in pazienti ospedalizzati per riacutizzazione di broncopneumopatia cronica ostruttiva.

	**Presenti**
• Prof. GianPiero Benetti – Clinico – Presidente	
• Dott. Alfonso Fossà - Direttore Sanitario ASL MilanoDue2	
• Sig.ra Rossana Rinaldi - Infermiera componente NOC ASL MilanoDue	Rinaldi R.
• Prof. Pietro Bonini – Rappresentante volontariato	Pietro Bonini
• Dott. Giorgio Tresoldi – Rapp.te Medicina Generale Territoriale	Giorgio Tresoldi
• Dott. Emerico Maurizio Panciroli – Esperto in Biostatistica	[illegible]
• Dott. Roberto Giunta – Medico Legale e delle Assicurazioni	[illegible]
• D.ssa M.A. Ferretti – Direttore Dip. Cure Primarie ASL MilanoDue	
• Don Carlo Millefanti - Esperto in bioetica	
• Dott. Franco Pazzucconi - Farmacologo	[illegible]
• Dott.ssa Isabella Ruggeri – Direttore Serv. Assistenza Farmac.	[illegible]
• Prot. Sergio Sforzini – Clinico	[illegible]

Il segretario: Sig.ra Paola Locatelli Paola Locatelli

SERVIZIO SANITARIO REGIONALE
EMILIA-ROMAGNA

Azienda Ospedaliero-Universitaria di Modena
Azienda Unità Sanitaria Locale di Modena

Comitato Etico Provinciale

UNIVERSITÀ DEGLI STUDI
DI MODENA E REGGIO EMILIA
Ateneo fondato nel 1175

Il Segretario

Prot. n. 2716 / C.E.

Modena, lì 22 SET 2006

Chiar.mo Prof.
Leonardo Fabbri
Malattie Apparato Respiratorio
Università Modena e Reggio Emilia

Ministero della Salute
Agenzia del Farmaco –AIFA
Via della Sierra Nevada 60
00144 ROMA

OGGETTO: Pratica 118/06 "Confronto tra un regime terapeutico basato sui livelli sierici di procalcitonina rispetto ad un regime terapeutico standard in pazienti ospedalizzati per riacutizzazione di broncopneumopatia cronica ostruttiva". NO Sponsor.

**Co-SPERIMENTATORI**	Richeldi Luppi Franco De Carlo Mosconi D'Agostino Mantovani Plebani
**CENTRO COORDINATORE**	Malattie Apparato Respiratorio di Modena
**OBIETTIVO PRIMARIO**	confrontare due regimi di terapia antibiotica per il trattamento di pazienti BPCO ospedalizzati per riacutizzazione infettiva di malattia
**DISEGNO DELLO STUDIO**	Studio sperimentale di fase IV, prospettico, longitudinale, policentrico nazionale
**N° PAZIENTI PER CENTRO**	23
**DURATA DELLO STUDIO**	10/06-04/08

Il Comitato Etico per la valutazione dei protocolli di sperimentazione dei farmaci e di ricerca, si è riunito il 05/09/06

- esaminato il protocollo di studio di cui all'oggetto,
- unanime esprime

PARERE UNICO FAVOREVOLE alla conduzione dello studio di cui all'oggetto.

Si ricorda che in riferimento ai rapporti sullo stato di avanzamento dello studio (D.M. 18/03/1998), lo Sperimentatore deve inviare al Comitato Etico annualmente, o più di frequente se richiesto dallo stesso, i riassunti scritti della situazione dello studio e deve inoltre fornire tempestivamente rapporti scritti in merito ad eventuali cambiamenti che influenzino la conduzione dello studio e/o che aumentino il rischio per i soggetti.

Distinti saluti

Dott. Saverio Santachiara

Allegato: elenco componenti Comitato Etico Provinciale/elenco documenti esaminati/Approv DS
Ref. Bianchi N\Approv. PARUNICO 118/06.doc

Comitato Etico Provinciale
Via del Pozzo, 71 – 41100 Modena
T.+39.059.422.2990 – F.+39.059.422.2324
comitatoetico@policlinico.mo.it

Azienda Ospedaliero-Universitaria di Modena
Via del Pozzo, 71 – 41100 Modena
T.+39.059.422.2111 – F.+39.059.422.4905
Partita IVA 02241740360 www.policlinico.mo.it

SERVIZIO SANITARIO REGIONALE
EMILIA-ROMAGNA

Azienda Ospedaliero-Universitaria di Modena
Azienda Unità Sanitaria Locale di Modena

UNIVERSITÀ DEGLI STUDI
DI MODENA E REGGIO EMILIA
Ateneo fondato nel 1175

Comitato Etico Provinciale

Il Segretario

OGGETTO: Pratica 118/06 "Confronto tra un regime terapeutico basato sui livelli sierici di procalcitonina rispetto ad un regime terapeutico standard in pazienti ospedalizzati per riacutizzazione di broncopneumopatia cronica ostruttiva". NO Sponsor

DOCUMENTI ESAMINATI DAL COMITATO ETICO

- ✓ Griglia riassuntiva (anche formato elettronico)
- ✓ Protocollo in esteso del 02/09/06
- ✓ Sinossi del protocollo versione del 02/09/06
- ✓ Dichiarazione del Proponente e dello Sperimentatore di attenersi alla Dichiarazione di Helsinky e autorizzazione del responsabile della Unità Operativa e del Direttore del Dipartimento ad eseguire la sperimentazione
- ✓ Testo del foglio informativo per il paziente
- ✓ Modulo per il consenso informato scritto
- ✓ Testo della lettera da inviare al medico curante
- ✓ Case Report Form
- ✓ Questionari per la valutazione dei sintomi respiratori SF-36, CCIQ, BDI e TDI
- ✓ Questionario di farmaco economia
- ✓ Dichiarazione di rientrare nella copertura assicurativa Aziendale
- ✓ Richiesta di esenzione dagli oneri amministrativi
- ✓ Pubblicabilità dei dati
- ✓ Bozza di convenzione economica
- ✓ Elenco dei centri partecipanti alla ricerca specificando il Centro Coordinatore
- ✓ Breve curriculum vitae dello Sperimentatore e sua dichiarazione di disporre del tempo e dei mezzi necessari per condurre la sperimentazione clinica proposta
- ✓ Dichiarazione di non avere conflitto di interesse personale o familiare con i risultati della ricerca

Il Comitato Etico rilascia questo documento in conformità ai propri regolamenti costitutivi e dichiara che il Comitato Etico è organizzato ed opera nel rispetto delle norme di buona pratica clinica (GCP-ICH) e degli adempimenti previsti dall'allegato al Decreto Ministeriale del 15/07/97 (Recepimento delle linee guida dell'Unione Europea di buona pratica clinica per l'esecuzione delle sperimentazioni cliniche dei medicinali) e DL n. 211 del 24/06/03 recante l'attuazione della direttiva 2001/20/CE.

Data di esame: 05/09/06

(Firma il Segretario del Comitato)

22 SET 2006
(gg/mm/aa)

Comitato Etico Provinciale
Via del Pozzo, 71 – 41100 Modena
T.+39.059.422.2990 – F.+39.059.422.2324
comitatoetico@policlinico.mo.it

Azienda Ospedaliero-Universitaria di Modena
Via del Pozzo, 71 – 41100 Modena
T.+39.059.422.2111 – F.+39.059.422.4905
Partita IVA 02241740360 www.policlinico.mo.it

A. O. MONALDI - NAPOLI
Azienda di Rilievo Nazionale e di Alta Specializzazione
Via L. Bianchi - 80131 - NAPOLI - Tel 081 /7061111

COMITATO ETICO-SCIENTIFICO
dell'Azienda Ospedaliera Monaldi

Parere n. 32 del 15.11.2006

OGGETTO: Studio sperimentale "Confronto fra un regime terapeutico basato sui livelli sierici di procalcitonina rispetto ad un regime terapeutico standard in pazienti ospedalizzati per riacutizzazione di broncopneumopatia cronica ostruttiva". (Clinica di Malattie dell'Apparato Respiratorio dell'Università degli Studi di Modena e Reggio Emilia ed AIFA)

Nella seduta del 15.11.2006 questo Comitato Etico-Scientifico così composto:

Prof. Ernesto CATENA	Presidente Comitato Etico-Scientifico<br>Esperto di Area delle Malattie dell'Apparato Respiratorio	Presente	
Prof. Francesco BARIFFI	Esperto in malattie dell'Apparato Respiratorio e Tisiologia	Presente	
Dott.ssa Loredana CAFARO	Rapp.te del Tribunale dei diritti del malato	Presente	
Prof. Massimo CAFIERO	Esperto Area Medica	Presente	
Sig. Ciro CARBONE	Rappresentante del Collegio IPASVI		Assente
Prof. Antonello CRISCI	Esperto di Medicina legale delle Assicurazioni	Presente	
Prof. Aldo CELENTANO	Esperto di biostatistica	Presente	
Prof. Ennio DE VINCENTIIS	Esperto Area Chirurgica		Assente
Prof.ssa Vittoria DI MARTINO	Rapp.te dell'Ass.ne Volontari Ospedalieri	Presente	
Prof. Giovanni DI MINNO	Clinico esperto di farmacoterapia	Presente	
Dott. Marco PAPA	Direttore Sanitario "ex officio" A.O. "V. Monaldi"	Presente	
Prof. Francesco GRANDE	Presidente onorario	Presente	
Prof. Goffredo SCIAUDONE	Esperto di bioetica	Presente	
Dott.ssa M. Luisa PLACELLA	Dirigente "ex officio" della Farmacia A.O "V. Monaldi"		Assente

- Esaminata la richiesta del Direttore della Clinica di Malattie dell'Apparato Respiratorio dell'Università degli Studi di Modena e Reggio Emilia e dell'AIFA, relativa allo studio in oggetto, da svolgere presso la U.O.C. Medico-chirurgica di oncologia e patologia toracica c/o A.O. "Monaldi".
- Preso atto che lo studio presentato è giustificato scientificamente ed eticamente ed anche per quanto concerne gli esami valutativi previsti ed il rapporto rischio/beneficio.

ESPRIME IL PARERE FAVOREVOLE

- All'effettuazione dello studio di cui sopra che dovrà essere condotto ai sensi della normativa in materia;
- e ricorda al responsabile della sperimentazione che è tenuto a far pervenire una relazione trimestrale sullo svolgimento dello studio oltre alla relazione finale sull'esito dello stesso.

IL SEGRETARIO
- Dott. Sergio Giaquinto -

IL PRESIDENTE DEL
COMITATO ETICO-SCIENTIFICO
- Prof. Ernesto Catena -

A.O. MONALDI - NAPOLI
Azienda di Rilievo Nazionale e di Alta Specializzazione
Via L. Bianchi - 80131 - NAPOLI -

Prot n° 128
del 07/12/2006

Spett.le Prof. Pierfranco Conte
Dipartimento Integrato di Oncologia
ed Ematologia
Università degli Studi di Modena
e Reggio Emilia
Via Del Pozzo, 71 – 41100
Modena

e p.c. Alla Direzione della Farmacia Ospedaliera
Al Direttore del Servizio Gestione E.F.
Al Prof. Serafino A. Marsico

OGGETTO: Sperimentazione clinica dal titolo: "Confronto fra un regime terapeutico basato sui livelli sierici di Procalcitonina rispetto ad un regime terapeutico standard in pazienti ospedalizzati per riacutizzazione di broncopneumopatia cronica ostruttiva". (Università degli Studi di Modena e Reggio Emilia ed A.I.F.A.)

Trasmetto per gli adempimenti di competenza, in uno al parere n. 32/06 del Comitato Etico-Scientifico e ai documenti esaminati, la delibera n. 606 del 06/12/2006 relativa allo studio in oggetto.

Il Direttore Generale
- Prof. Tullio Cusano -

A. O. MONALDI - NAPOLI
Azienda di Rilievo Nazionale e di Alta Specializzazione
Via L. Bianchi - 80131 - NAPOLI - Tel 081 /7061111

ELENCO DOCUMENTI ALLEGATI ED ESAMINATI DAL COMITATO ETICO-SCIENTIFICO. Sperimentazione clinica dal titolo: "Confronto fra un regime terapeutico basato sui livelli sierici di Procalcitonina rispetto ad un regime terapeutico standard in pazienti ospedalizzati per riacutizzazione di broncopneumopatia cronica ostruttiva". (Università degli Studi di Modena e Reggio Emilia ed A.I.F.A.)

1) Richiesta di autorizzazione alla conduzione dello studio (sperimentatore Prof. S.A. Marsico), datata 03/10/2006
2) Sinossi del Protocolloin lingua inglese – versione 24/07/2006
3) Sinossi del Protocollo in lingua italiana
4) Elenco degli Emendamenti al protocollo
5) Confidential Case Report Form
6) Chronic Cough Impact Questionnaire
7) Indice basale di dispnea (BDI)
8) Lettera dell'A.I.F.A. indicante le caratteristiche e la rilevanza scientifica dello studio in oggetto (datata 24/03/2006)
9) Parere favorevole del Comitato Etico Provinciale di Modena
10) Copia della polizza assicurativa
11) Elenco dei centri partecipanti allo studio
12) Scheda Informativa e Consenso informato per il paziente
13) Lettera per il medico curante
14) Proposta di convenzione

Valentina Ruggieri

Da: ce.sperimentazione@sanita.padova.it
Inviato: mercoledì 26 marzo 2014 11.36
A: valentina.ruggieri@unimore.it
Oggetto: Rif: Richiesta num approvazione etica centro satellite

nostro numero di riferimento per lo studio 1351P, responsabile Prof. Renzo Zuin approvato nella seduta del 13/11/2006

Segreteria del Comitato Etico per la Sperimentazione Clinica (CESC) della Provincia di Padova
Azienda Ospedaliera di Padova
Via Giustiniani, 1 - 35128 Padova
Tel. 0498212341-42 fax 0498212827
e-mail: ce.sperimentazione@sanita.padova.it

-----"Valentina Ruggieri" <valentina.ruggieri@unimore.it> ha scritto: -----

> Per: <ce.sperimentazione@sanita.padova.it>
> Da: "Valentina Ruggieri" <valentina.ruggieri@unimore.it>
> Data: 25/03/2014 16.57
> Cc: "'Alessia VERDURI'" <alessia.verduri@unimore.it>
> Oggetto: Richiesta num approvazione etica centro satellite
>
> Spett.le Comitato Etico,
>
> la presente e-mail, per chiedere il vostro numero di riferimento (pratica, protocollo, procedimento…) di approvazione e data della seduta del seguente studio AIFA FARM58J2XH: "COMPARISON OF A SERUM PROCALCITONIN (PRO-CT) GUIDED TREATMENT PLAN WITH THE STANDARD GUIDELINE RECOMMENDED ANTIBIOTIC TREATMENT PLAN FOR PATIENTS HOSPITALIZED WITH A DIAGNOSIS OF EXACERBATION OF COPD".
>
> Si allega parere favorevole del centro coordinatore Modena (Prof Leonardo Fabbri).
>
> In attesa di un vostro cortese riscontro, porgo cordiali saluti.
>
> Valentina Ruggieri
>
> *Dott.ssa Valentina Ruggieri*

Clinical Research Coordinator

Clinica Malattie Apparato Respiratorio

Policlinico di Modena

Via del Pozzo 71,

I-41124 MODENA

Tel. 0039 059 422 5762

Fax 0039 059 422 3113

email valentina.ruggieri@unimore.it

[allegato "Approvazione Centro Modena.pdf" rimosso da Servizio Comitato Etico per la Sperimentazione AOP/Direzione Generale/servam/People/sanita.padova.it]
=

UNIVERSITÀ DEGLI STUDI DI PARMA

COMITATO DI ETICA

Parma, 6 novembre 2006

Chier.mo
Prof. Dario Olivieri
Direttore
Sezione di Clinica Pneumologica
Via Gramsci, 14
PARMA

Prot. N. 63/CE/06

Oggetto : PROTOCOLLO DI STUDIO: "Confronto fra un regime terapeutico basato sui livelli sierici di procalcitonina rispetto ad un regime terapeutico standard in pazienti ospedalizzati per riacutizzazione di broncopneumopatia cronica ostruttiva".

In base alla delega conferitami dal Comitato Etico nella seduta del 19.10.2006, preso atto che la documentazione pervenuta in data odierna è conforme alle modifiche richieste dal Comitato stesso nella riunione poc'anzi menzionata, autorizzo l'inizio della sperimentazione in oggetto.

Cordiali saluti

Il Presidente

Prof. Almerico Novarini

43100 Parma - Italia - Via A. Gramsci, 14 - Tel. 0521.033117 - Fax 0521.033118 - e-mail: com.etic@unipr.it

FONDAZIONE
IRCCS POLICLINICO *"SAN MATTEO"*
Istituto di Ricovero e Cura a Carattere Scientifico
di diritto pubblico (D.M. 28/4/2006)
C.F. 00303490189 - P. IVA 00580590180
V.le Golgi, 19 - 27100 PAVIA
Tel. 0382 5011

COMITATO DI BIOETICA

(istituito, in conformità alle disposizioni del Decreto del Ministero della Sanità del 27/04/1992, con deliberazione commissariale n. 1354/92 del 19/10/1992, modificato nella sua composizione, in conformità alle disposizioni del Decreto del Ministero della Sanità del 18/03/1998, con deliberazione commissariale n. 1231/98 del 20/07/1998)

ESTRATTO DEL VERBALE DEI LAVORI PER LA SEDUTA DEL 13/11/2006

In data 13/11/2006 alle ore 16.15, presso la Direzione della Fondazione, si è riunito il "Comitato di Bioetica" per discutere il seguente ordine del giorno:

1. Approvazione verbale dei lavori per la seduta del 16/10/2006;
2. Aggiornamento sulle iniziative formative e culturali;
3. Comunicazioni dell'Ufficio di Segreteria;
4. Valutazione eventi avversi in studi coordinati dalla Fondazione o per pazienti arruolati presso la Fondazione;
5. Richieste di parere sulla effettuazione di studi clinici presso la Fondazione;
6. Comunicazioni relative a studi clinici già valutati dal Comitato, ma non ancora approvati;
7. Comunicazioni relative a studi clinici già approvati dal Comitato;
8. Varie ed eventuali.

Sono presenti i seguenti Componenti:

I COPIA

Prof. Arturo MAPELLI
Prof. Giorgio RONDINI
Dr.ssa Luigina ZAMBIANCHI
Prof. Carlo Alberto REDI
Dr. Vanni BASCAPE'
Prof. Edoardo ASCARI
Don Giovanni LODIGIANI
Prof. Francantonio BERTE'
Prof.ssa Giovanna RUBERTO
Dr. Carmine TINELLI

Il Presidente del Comitato, Prof. Arturo Mapelli, verificata la presenza del numero legale dei componenti, dichiara aperti i lavori.

Omissis

**Tipo:** sperimentazione farmaci	**Fase:** IV
**Farmaco:** Terapia Antibiotica (Amoxicillina/Acido Clavulanico o Chinolonici)	
**Proponente:** Università degli Studi di Modena e Reggio Emilia	
**Struttura:** Malattie dell'Apparato Respiratorio	
**Responsabile Scientifico:** Prof. Ernesto Pozzi	
**Protocollo:** Comparison of a serum procalcitonin (pro-CT) guided treatment plan with the standard guideline recommended antibiotic treatment plan for patients hospitalized with a diagnosis of exacerbation of COPD.	
**Codice Prot.:** ----------	**Data Prot.:** 2/9/2006
**Modulo di Informazione e di Consenso del Paziente (versione – data):** documento privo di indicazione di versione – data; lettera per il medico curante	
**Case Report Form (versione – data):** bozza del 31/7/2006	
**Investigator's Brochure:** ----------	
**Polizza assicurazione:** Fondazione I.R.C.C.S. Policlinico San Matteo, Pavia	
**Centro Coordinatore:** Università degli Studi di Modena e Reggio Emilia - Parere Unico del 5/9/2006	
**Codice Osservatorio:** 2006-005354-68	
**Ulteriore Documentazione:** Questionario sullo stato di salute SF-36 (versione 1.6); Chronic Cough Impact Questionnaire; Indici di Dispnea (BDI - TDI); scheda di segnalazione eventi avversi.	
**Nr. Pazienti:** circa 24	**Compenso:** ------------
**Note:** --------	
**Parere: Favorevole***	
**Condizioni:**<br>- **fatta salva la copertura dei costi aggiuntivi con il finanziamento AIFA assegnato;**<br>- **fatta salva la modifica della scheda informativa per il paziente a pag. 2, seconda riga "Questo *potrebbe comportare* per Lei il vantaggio di non essere sottoposto inutilmente ad una terapia antibiotica non necessaria. In breve, se Lei venisse selezionato nel gruppo sperimentale *le verrà sospesa la* terapia, mentre se Lei dovesse continuare...".**	

N.B.*:

1) Il parere si intende vincolato al rispetto delle eventuali condizioni di cui sopra, della normativa nazionale e regionale (ivi compresa quella in materia di tutela dei dati personali) e dei regolamenti dell'Istituto e **con la clausola che i risultati dello studio non potranno essere utilizzati a fini commerciali.**
2) Gli eventuali campioni biologici prelevati, previo consenso, da pazienti arruolati nello studio dovranno essere conservati solo per il tempo necessario allo svolgimento dello stesso. Qualsiasi ulteriore utilizzo dovrà essere preventivamente autorizzato, per iscritto, dai pazienti interessati.
3) Fatta salva l'assenza di qualsiasi conflitto di interessi in capo a tutto il personale coinvolto nello studio.
4) Si raccomanda l'inserimento nel foglio di informazioni al paziente della frase "*Qualora disponga di un'assicurazione medica privata è opportuno che verifichi con l'assicuratore che la Sua partecipazione allo studio non abbia alcuna ripercussione sulla Sua copertura assicurativa medica o sulla vita, prima di acconsentire a parteciparvi.*"

Omissis

Alle ore 19.40 il Comitato conclude i propri lavori.

IL PRESIDENTE
(Prof. Arturo Mapelli)

A. Mapelli

AZIENDA OSPEDALIERO-UNIVERSITARIA PISANA

(L.R.T. 24 febbraio 2005, n. 40)

Sede legale: Via Roma, 67 - 56126 PISA - Tel. 050996111

Regione 090 - Azienda 901

SEGRETERIA AMMINISTRATIVA DEL COMITATO DI BIOETICA
tel. 050996392 050996247 050996287 – fax 050996293

Da *SEGRETERIA AMMINISTRATIVA DEL COMITATO DI BIOETICA*

Prot. n° 47802

Pisa, li 20/12/2006

Egr.
PROF. PIERLUIGI PAGGIARO
U.O. PNEUMOLOGIA E FISIOPATOLOGIA RESPIRATORIA UNIVERSITARIA
P.O. CISANELLO

Studio n. 2218

Oggetto: **Approvazione dello studio n. 2218– Protocollo**

In allegato alla presente, si invia il verbale relativo alla seguente sperimentazione: "Confronto fra un regime terapeutico basato sui livelli sierici di procalcitonina rispetto ad uno standard in pazienti ospedalizzati per riacutizzazione di broncopneumopatia cronica ostruttiva"Codice Eudract esaminata dal Comitato nella seduta del 14/12/2006.

Nelle comunicazioni successive (comprese quelle relative agli eventi avversi), si richiede gentilmente, di riportare il **numero dello studio** citato in oggetto.

Il Comitato Etico si riserva la facoltà di verificare il corso della sperimentazione autorizzata.

Lo sperimentatore dovrà attenersi, nell'esecuzione dello studio, a quanto previsto dal D.M. 15 luglio 1997, art. 4 e in particolare:

- 4.1 Qualifiche dello Sperimentatore ed Accordi;
- 4.2 Adeguatezza delle Risorse;
- 4.3 Assistenza medica dei Soggetti che partecipano allo Studio;
- 4.4 Comunicazioni con il Comitato Etico Indipendente (IEC);
- 4.5 Aderenza al Protocollo;
- 4.6 Prodotto/i in Sperimentazione;
- 4.7 Procedure di Randomizzazione e Apertura del Cieco;
- 4.8 Consenso Informato dei Soggetti Coinvolti nello Studio;
- 4.9 Documentaizone e Rapporti;
- 4.10 Rapporti sullo stato di Avanzamento;
- 4.11 Rapporti sulla Sicurezza;

AZIENDA OSPEDALIERO-UNIVERSITARIA PISANA
(L.R.T. 24 febbraio 2005, n. 40)
Sede legale: Via Roma, 67 - 56126 PISA - Tel. 050996111
Regione 090 - Azienda 901

SEGRETERIA AMMINISTRATIVA DEL COMITATO DI BIOETICA
tel. 050996392 050996247 050996287 – fax 050996293

- 4.12 Conclusione Anticipata o Sospensione dello Studio;
- 4.13 Rapporto Finale dello Sperimentatore: **lo sperimentatore deve fornire al Comitato Etico di competenza, un riassunto dell'esito dello studio ed alle autorità regolatorie ogni eventuale rapporto richiesto.**

Distinti saluti.

Il Segretario
(Franca Cossu)
FCossu

SST Servizio Sanitario della Toscana

AZIENDA OSPEDALIERO - UNIVERSITARIA PISANA

(L. R. T. 24 febbraio 2005, n. 40) Regione 090 - Azienda 901
Sede legale: Via Roma, 67 - 56126 PISA – Tel. 050996111

SEGRETERIA AMMINISTRATIVA DEL COMITATO DI BIOETICA
Tel. 050996392 050996247 050996287 - Fax 050996293

Comitato per la Sperimentazione Clinica dei Medicinali

Arrivato il 12-10-2006

Studio n° 2218 / 2006

Titolo "Confronto fra un regime terapeutico basato sui livelli sierici di procalcitonina rispetto ad uno standard in pazienti ospedalizzati per riacutizzazione di broncopneumopatia cronica ostruttiva"

Tipo OSSERVAZIONALE

Codice Eudract

Fase

Protocollo

Farmaco

Principio attivo

Codice

Acronimo

Sponsor AGENZIA ITALIANA DEL FARMACO (AIFA)

C. R. O.

Centro di Ricerca AZIENDA OSPEDALIERO-UNIVERSITARIA PISANA
VIA ROMA, 67 - 56126 PISA

Reparto U.O. PNEUMOLOGIA E FISIOPATOLOGIA RESPIRATORIA UNIVERSITARIA, P.O. CISANELLO

Sperimentatore PROF. PIERLUIGI PAGGIARO

C. Coordinatore UNIVERSITA' DEGLI STUDI DI MODENA E REGGIO EMILIA

Tipo STUDIO LIBERO

Data Seduta 14/12/2006

Esito **POSITIVO**

Il Comitato per la sperimentazione clinica dei medicinali, in osservanza a quanto previsto dal D.M. 15 luglio 1997; dalla Circolare n. 15 del 15 ottobre 2000 e dal D. Lgs. n. 211 del 24 giugno 2003, si è riunito per esaminare lo studio in oggetto ed in particolare la seguente documentazione:

Servizio Sanitario della Toscana

AZIENDA OSPEDALIERO - UNIVERSITARIA PISANA
(L. R. T. 24 febbraio 2005, n. 40)
Sede legale: Via Roma, 67 - 56126 PISA - Tel. 050996111 *Regione 090 - Azienda 901*

SEGRETERIA AMMINISTRATIVA DEL COMITATO DI BIOETICA
Tel. 050996392 050996247 050996287 - Fax 050996293

Comitato per la Sperimentazione Clinica dei Medicinali

	Ver	Data ver.	Annotazioni
LISTA CENTRI PARTECIPANTI			
MODULO DI CONSENSO INFORMATO PER IL PAZIENTE			
SCHEDA DI INFORMAZIONE PER IL PAZIENTE			
LETTERA AL MEDICO CURANTE			
PROTOCOLLO		24/07/2006	
SCHEDA DI SEGNALAZIONE EVENTO AVVERSO			
PARERE FAVOREVOLE CENTRO COORDINATORE		05/09/2006	
INDICE BASALE DI DISPNEA			
CHRONIC COUGH IMPACT QUESTIONNAIRE			
SINOSSI DEL PROTOCOLLO			
QUESTIONARIO SULLA SALUTE	1.6 SF-36		ITALIAN VERSION

DELIBERAZIONE

Il Comitato per la sperimentazione clinica dei medicinali accetta il parere unico favorevole espresso dal comitato etico del centro coordinatore ed **APPROVA** la sperimentazione in oggetto con la raccomandazione di chiarire l'incongruenza tra la versione del protocollo esaminata dal comitato etico del centro coordinatore, che è datata 02/09/2006 e quella inviata a questo Comitato Etico (che reca la data del 24/07/2006).

COMPONENTI DEL COMITATO PER LA SPERIMENTAZIONE CLINICA DEI MEDICINALI

Presenti alla seduta:

Prof. Roberto BARSOTTI — Presente
Professore Ordinario Diritto Internazionale-Esp. materia giuridica-Presidente

Prof. Stefano BOMBARDIERI — Presente

AZIENDA OSPEDALIERO - UNIVERSITARIA PISANA
(L. R. T. 24 febbraio 2005, n. 40)
Sede legale: Via Roma, 67 - 56126 PISA – Tel. 050996111 *Regione 090 - Azienda 901*

SEGRETERIA AMMINISTRATIVA DEL COMITATO DI BIOETICA
Tel. 050996392 050996247 050996287 - Fax 050996293

Comitato per la Sperimentazione Clinica dei Medicinali

Professore Ordinario di Reumatologia - Clinico

Dr. Maido CASTIGLIONI<br>Direttore U.O. Medicina Generale IV - Clinico	Presente
Dr.ssa Flora COSCETTI<br>Direttore Dip. Infermicristico - Dirigente Infermieristico	**ASSENTE**
Dr.ssa Luana DAL CANTO<br>Farmacista del Servizio Farmaceutico Ospedaliero - Farmacista	Presente
Prof. Romano DANESI<br>Professore Straordinario di Farmacologia medica - Farmacologo	Presente
Prof. Stefano DEL PRATO<br>Professore Ordinario Diabetologia - Clinico	Presente
Prof. Ranieri DOMENICI<br>Professore Ordinario di Medicina Legale - Medico Legale	Presente
Dr. Claudio FAVRE<br>Direttore U.O. Oncoematologia pediatrica - Pediatra	Presente
Dr. Giuseppe FIGLINI<br>Federazione Medici di Medicina Generale-Medico Medicina Generale	**ASSENTE**
Prof. Gianfranco FIORAVANTI<br>Professore Ordinario-Preside Facoltà Lettere e Filosofia - Esp. Bioetico	**ASSENTE**
Dr.ssa Gabriella GIULIANO<br>Dirigente Sanitario Azienda Ospedaliero-Universitaria Pisana - Biostatistico	**ASSENTE**
Prof. Bruno MACCHIA<br>Professore Ordinario di Chimica Farmaceutica - Esp. politiche farmaco	Presente
Prof. Luigi MURRI<br>Preside Facoltà Medicina e Chirurgia - Clinico	Presente
Dr.ssa Anna TONGIORGI<br>Rappresentante Ass. Oncologica Pisana-Rappresentante volontariato	Presente
Sig.ra FRANCA COSSU<br>Funzionario Azienda Ospedaliero-Universitaria Pisana – Segretario	Presente

AZIENDA OSPEDALIERO - UNIVERSITARIA PISANA

(L. R. T. 24 febbraio 2005, n. 40)

Sede legale: Via Roma, 67 - 56126 PISA – Tel. 050996111

Regione 090 - Azienda 901

SEGRETERIA AMMINISTRATIVA DEL COMITATO DI BIOETICA

Tel. 050996392 050996247 050996287 - Fax 050996293

Comitato per la Sperimentazione Clinica dei Medicinali

I SOPRA ELENCATI COMPONENTI DEL COMITATO DICHIARANO CHE SI ASTERRANNO DAL PRONUNCIARSI SU QUELLE SPERIMENTAZIONI PER LE QUALI POSSA SUSSISTERE UN CONFLITTO DI INTERESSI DI TIPO DIRETTO OD INDIRETTO

f.to IL PRESIDENTE
Prof. Roberto Barsotti

Il Segretario
(Franca Cossu)

FCossu

SERVIZIO SANITARIO REGIONALE
EMILIA-ROMAGNA
Azienda Ospedaliera di Reggio Emilia

Arcispedale S. Maria Nuova
Direzione Sanitaria
Direzione Operativa

Dott. Giorgio Mazzi - Direttore

Reggio Emilia, lì 25 SET. 2006

Prot. n. 24374

COMITATO ETICO

Dott.ssa Elisa VERATELLI
Dipartimento di Malattie Respiratorie
Università degli Studi di Modena e Reggio Emilia
Via Del Pozzo, 71
41100 MODENA

Dott. Luigi ZUCCHI
Direttore S.C. di Pneumologia
Azienda Ospedaliera
Arcispedale Santa Maria Nuova
SEDE

Dott. Giorgio MAZZI
Direttore Operativo
Azienda Ospedaliera
Arcispedale Santa Maria Nuova
SEDE

e p.c. Agenzia Italiana del Farmaco
Sperimentazione e Ricerca
Via della Sierra Nevada, 60
00144 ROMA EUR

Oggetto: Studio approvato secondo Bando AIFA 2005 "Confronto di uno schema di trattamento antibiotico deciso in accordo ai livelli sierici di procalcitonina rispetto al trattamento standard in pazienti ricoverati per riacutizzazione di broncopneumopatia cronica ostruttiva (BPCO)" proposto dalla Clinica di Malattie dell'Apparato Respiratorio dell'Università degli Studi di Modena e Reggio Emilia presso la S.C. di Pneumologia. 90/2006

Allegati: n. 1

Si comunica che il Comitato Etico nella seduta del 18.09.2006 ha esaminato lo studio all'oggetto ed ha espresso parere favorevole alla sua effettuazione.

Relativamente al Foglio Informativo/Modulo di Consenso Informato si chiede di correggere laddove presente l'indicazione "In questa Clinica Universitaria..." e di inserire l'informazione che lo studio è coperto dalla polizza assicurativa dell'Azienda Ospedaliera.

Si rimane pertanto in attesa di ricevere Il Foglio Informativo modificato come richiesto.

Si trasmette in allegato l'elenco dei componenti il Comitato presenti alla suddetta seduta del 18.09.2006.

Cordiali saluti.

p. Il Presidente
(Dr. Nicola Magrini)
Il segretario

SC Affari Generali e Legali
Direttore: Dott. Fabio Zimarelli

COMITATO ETICO

(istituito in conformità alle disposizioni del Decreto del Ministero della Sanità del 18/03/1998, con deliberazione n. 566 dd. 16/12/2004)

PRESENZE DELLA SEDUTA 18/12/2006

PRESENTI:

- Prof. Cesare CAMPELLO - Presidente
  Direttore Dipartimento di Scienze Medicina Pubblica Università degli Studi di Trieste
- Mons. Tarcisio BOSSO
  Professore presso la Facoltà Teologia Alta Italia, licenziato in Diritto Civile e Canonico
- Dott.ssa Lucia PELUSI
  Dirigente Medico Presidio Ospedaliero di Cattinara Azienda Ospedaliero-Universitaria Ospedali Riuniti di Trieste
- Dott.sa Maria Chiara TREVISAN
  Dirigente S. C. Farmacia Azienda Ospedaliero-Universitaria Ospedali Riuniti di Trieste
- Prof. Carlo SCORRETTI
  U.C.O. Medicina Legale Università di Trieste
- Sig.ra Patrizia TRAMARIN
  Coordinatore Infermieristico Ospedale Maggiore - Azienda Ospedaliero-Universitaria
- Prof. Ugo TRAVERSA
  Farmacologo Dipartimento Scienze Biomediche dell'Università di Trieste
- Dott.ssa Antonella ZADINI
  Dirigente S.C. Riabilitazione Azienda Ospedaliero-universitaria Ospedali Riuniti di Trieste
  Rappresentante Associazioni Volontariato Provincia di Trieste
- Dott.ssa Daniela ZARO
  Responsabile U.O. Affari Giuridici Azienda per i Servizi Sanitari ASS n. 1 Triestina

ASSENTI GIUSTIFICATI:

- Dott. Sergio MINUTILLO – Vicepresidente
  Responsabile S.C. Medicina d'Urgenza Azienda Ospedaliero-Universitaria Ospedali
- Prof. Mauro GIACCA
  Direttore Centro Internazionale d'Ingegneria Genetica e Biotecnologia (ICGEB) di Trieste
- Dott. Nicola DELLI QUADRI
  Direttore Sanitario Azienda Ospedaliero-Universitaria Ospedali Riuniti di Trieste

I sopraindicati componenti del Comitato Etico indipendente dichiarano di astenersi dal pronunciarsi sulle sperimentazioni per le quali sussiste conflitto di interesse di tipo diretto e indiretto.

È presente la Dott.ssa Antonia Antonioli, segretario verbalizzante.

...omissis

Approvazione dei seguenti studi e/o sperimentazioni e delle decisioni sotto riportate:

N. ORDINE 55/2006
"COMPARISON OF A SERUM PROCALCITONIN (PRO-CT) GUIDED TREATMENT PLAN WITH THE STANDARD GUIDELINE RECOMMENDED ANTIBIOTIC TREATMENT PLAN FOR PATIENTS HOSPITALIZED WITH A DIAGNOSIS OF EXACERBATION OF COPD" - CONFRONTO FRA UN REGIME TERAPEUTICO BASATO SUI LIVELLI SIERICI DI PROCALCITONINA RISPETTO AD UN REGIME TERAPEUTICO STANDARD IN PAZIENTI OSPEDALIZZATI PER RIACUTIZZAZIONE DI BRONCOPNEUMOPATIA CRONICA OSTRUTTIVA.

Il Comitato prende atto della scheda informativa per il paziente e del modulo per il consenso informato con le modifiche richieste nella seduta del 17 ottobre 2006 e approva definitivamente lo studio.

Questa parte del verbale viene redatta, approvata e sottoscritta seduta stante.

omissis...

Letto e sottoscritto:

Prof. Cesare CAMPELLO – Presidente

Mons. Tarcisio BOSSO

Dott.ssa Lucia PELUSI

Prof. Carlo SCORRETTI

Sig.ra Patrizia TRAMARIN

Dott.ssa Maria Chiara TREVISAN

Prof. Ugo TRAVERSA

Dott.ssa Antonella ZADINI

Dott.ssa Daniela ZARO

REGIONE DEL VENETO

COPIA

AZIENDA UNITA' LOCALE SOCIO-SANITARIA N. 6 "VICENZA"

DELIBERAZIONE

n. 37 del 31/01/2007

OGGETTO

Autorizzazione sperimentazioni cliniche (Comitato Etico per la sperimentazione clinica della Provincia di Vicenza - seduta del 20.12.2006).

Proponente: Servizio Farmaceutico Territoriale
Anno Proposta: 2007
Numero Proposta: 26

COMITATO ETICO PER LA SPERIMENTAZIONE CLINICA DELLA PROVINCIA DI VICENZA

SEDUTA DEL 20 DICEMBRE 2006

Dott. Eugenio FANTUZ
ASS.GIUST. Direttore Sanitario ULSS n. 6
Dott. Francesco PIETROBON
PRESENTE Direttore Sanitario ULSS n. 3
Dott. Pier Paolo BENETOLLO
ASS.GIUST. Direttore Sanitario ULSS n. 4
Dott. Giampaolo STOPAZZOLO
ASS.GIUST. Direttore Sanitario ULSS n. 5
Dott. Roberto GALLO
ASS.GIUST. Direttore Serv. Farmaceutico Territ. ULSS n. 6
Dott.ssa Maria Sofia COGATO
PRESENTE Serv. Farmacia ULSS n. 3
Dott.ssa Maria GIARDINO
ASS.GIUST. Direttore Serv. Farmaceutico Territ. ULSS n. 4
Dott.ssa Maria Paola CARIOLATO
PRESENTE Serv. Farmaceutico Territ. ULSS n. 5
Dott. Daniele BUSETTO
PRESENTE Specialista in Farmacologia Clinica- Internista Dirigente Medico Ospedaliero ULSS n. 6
Dott. Giorgio VESCOVO
PRESENTE Internista - Direttore S.C. Medicina Gen.ULSS n. 6
Prof. Francesco RODEGHIERO
ASS.GIUST. Ematologo - Direttore S.C. Ematologia ULSS n. 6
Dott. Francesco SALSA
PRESENTE già Direttore S.C. Neurologia ULSS n. 3
Dott. Salvatore CASTRONOVO
PRESENTE Internista - ematologo Dirigente Medico Ospedaliero ULSS n. 3
Dott. Giuseppe TESSAROLO
PRESENTE Internista - già Dirigente Medico Osp. ULSS n. 4
Dott. Claudio BUSANA
PRESENTE Psichiatra - Direttore Dip. Sal. Mentale ULSS n. 5
Prof. Paolo COLLESELLI
PRESENTE Pediatra - Direttore S.C. Pediatria ULSS n. 6
Dott. Renato TARTAROTTI
ASS.GIUST. Medico Medicina Gen. - indicato dall'Ordine dei Medici Chirurghi e Odontoiatri Prov. di Vicenza
Sig.ra Roberta SARTORI
ASS.GIUST. Coordinatore infermieristico Diplomata in materia sanitaria - ULSS n. 6
Dott.ssa Rina MIRANDOLA
PRESENTE Laureata in Scienze Statistiche Serv. Controllo di Gestione - ULSS n. 4
Prof. Giuseppe VERLATO
PRESENTE Professore Associato di Statistica Medica e Biometria Università di Verona - ULSS n. 5
Don Aldo DE TONI
ASS.GIUST. Esperto in bioetica Responsabile Pastorale Diocesana
Dott.ssa Licia SARTORI
PRESENTE Specialista in Medicina Legale e delle Assic. Responsabile Serv. Medicina Legale ULSS n. 4
Dott.ssa Maria Pia SARTORI
PRESENTE Esperta in materie giuridiche - ULSS n. 4
Dott. Alessandro DACOMO
PRESENTE Esperto monitoraggio studi clinici Direttore Serv. Farmacia Osped. - ULSS n. 6
Sig. Fiorenzo DOTTO
PRESENTE Rappresentante Associazioni Diritti del Malato - ULSS n. 5

Dott.ssa Paola VALPONDI
Segreteria Scientifica

Sig.ra Manuela PIVA
Segreteria Amministrativa

Scheda n. 3/facciata a)

Sperimentazione n. 22/06 CE PROV.LE

Protocollo:
Antibioticoterapia in BPCO

Versione: 03.08.06

Codice EUDRACT: ///

Fase: ///

Emendamento/i: 02.09.06

N° pazienti arr. localmente:

Prodotto:

Consenso Informato: presente

Investigator's Brochure Version: ///

Centro/i Coordinatore/i: C.E. di Modena

Sponsor: ///

Società di Gestione Studio: ///

Opzione:

Scheda n. 3 /facciata b) Sperimentazione n. 22/06 CE PROV.LE

Unità Operativa: Pneumologia - Ospedale di Bassano ULSS 3

Responsabile Studio: Dott. Calabro Stefano

Relatore: Dott. Calabro Stefano

Osservazioni: Il relatore espone ai presenti gli obiettivi che la sperimentazione si propone e dà risposta alle richieste di chiarimento. Congedato il relatore si valuta e si discute il protocollo di sperimentazione.

Parere: Il Comitato Etico all'unanimità esprime **parere favorevole** con richiesta di modifica del consenso informato per il paziente: a pag. 2 va riportato che "la partecipazione allo studio NON DOVREBBE COMPORTARE per Lei alcun rischio..."

ELENCO DOCUMENTI ALLEGATI ALLA DOMANDA DI AUTORIZZAZIONE:

protocollo di studio versione finale del 03.08.06
sinossi del protocollo versione finale del 03.08.06
elenco centri partecipanti allo studio
foglio informativo per il soggetto e consenso informato
lettera informativa per il medico curante
contratto per studio osservazionale
dichiarazione sulla proprietà dei dati
flow chart
scheda raccolta dati
copia bonifico bancario per spese CE
bozza convenzione economica
questionario sullo stato di salute

Valentina Ruggieri

**Da:**	comitatoetico@hpg23.it
**Inviato:**	lunedì 31 marzo 2014 14.09
**A:**	Valentina Ruggieri
**Cc:**	'Alessia VERDURI'; segrcomitatodibioetica@ospedaliriuniti.bergamo.it
**Oggetto:**	Rif: I: Richiesta num approvazione etica centro satellite

Gent.ma,
lo studio è stato valutato nella seduta del Comitato di Bioetica del 05/02/2007, delibera n. 418 del 26/03/2007, numero registro: 137/06, USC Pneumologia

Cordiali Saluti
Silvia Sala

Segreteria Tecnico-Scientifica
Comitato Etico della Provincia di Bergamo
A.O. Papa Giovanni XXIII
Piazza OMS, 1- 24127 Bergamo
☎ *035-2673340/3341*

comitatoetico@hpg23.it

-----"Valentina Ruggieri" <valentina.ruggieri@unimore.it> ha scritto: -----
Per: <segrcomitatodibioetica@ospedaliriuniti.bergamo.it>
Da: "Valentina Ruggieri" <valentina.ruggieri@unimore.it>
Data: 31/03/2014 01.21PM
Cc: "'Alessia VERDURI'" <alessia.verduri@unimore.it>
Oggetto: I: Richiesta num approvazione etica centro satellite

Gent.mi,

è possibile avere un riscontro sull’e-mail inoltrata?

Grazie.

Cordiali saluti

Valentina Ruggieri

Dott.ssa Valentina Ruggieri

Clinical Research Coordinator

Clinica Malattie Apparato Respiratorio

Policlinico di Modena

Via del Pozzo 71,

I-41124 MODENA

Tel. 0039 059 422 5762

Fax 0039 059 422 3113

email valentina.ruggieri@unimore.it

Da: Valentina Ruggieri [mailto:valentina.ruggieri@unimore.it]
Inviato: martedì 25 marzo 2014 16.25
A: 'segrcomitatodibioetica@ospedaliriuniti.bergamo.it'
Cc: 'Alessia VERDURI'
Oggetto: Richiesta num approvazione etica centro satellite

Spett.le Comitato Etico,

la presente e-mail, per chiedere il vostro numero di riferimento (pratica, protocollo, procedimento…) di approvazione e data della seduta del seguente studio AIFA FARM58J2XH: "COMPARISON OF A SERUM PROCALCITONIN (PRO-CT) GUIDED TREATMENT PLAN WITH THE STANDARD GUIDELINE RECOMMENDED ANTIBIOTIC TREATMENT PLAN FOR PATIENTS HOSPITALIZED WITH A DIAGNOSIS OF EXACERBATION OF COPD".

Si allega parere favorevole del centro coordinatore Modena (Prof Leonardo Fabbri).

In attesa di un vostro cortese riscontro, porgo cordiali saluti.

Valentina Ruggieri

Dott.ssa Valentina Ruggieri

Clinical Research Coordinator

Clinica Malattie Apparato Respiratorio

Policlinico di Modena

Via del Pozzo 71,

I-41124 MODENA

Tel. 0039 059 422 5762

Fax 0039 059 422 3113

email valentina.ruggieri@unimore.it

(Vedere file allegato:Approvazione Centro Modena.pdf)

[allegato "Approvazione Centro Modena.pdf" rimosso da Segreteria Comitato Etico/Direzione Sanitaria/ORBG]

Avviso di riservatezza - Il testo e gli eventuali documenti trasmessi contengono informazioni riservate al destinatario indicato. La seguente e-mail é confidenziale e la sua riservatezza é tutelata dal D.Lgs 196/2003. La lettura, copia o altro uso non autorizzato o qualsiasi altra azione derivante dalla conoscenza di queste informazioni sono rigorosamente vietate. Qualora abbiate ricevuto questo documento per errore siete cortesemente pregati di darne immediata comunicazione al mittente, ai numeri qui indicati e/o all'indirizzo dello stesso e di provvedere immediatamente alla sua distruzione. Si informa inoltre che il presente messaggio non ha natura personale, e che l'eventuale risposta potrà essere conosciuta da altro personale della nostra Azienda.

PROVINCIA AUTONOMA DI BOLZANO ALTO ADIGE
AZIENDA SANITARIA DI BOLZANO
COMITATO ETICO
Sede: Direzione Amministrativa
Via L. Böhler 5 – 39100 Bolzano
Tel. 0471 – 908232- Fax 0471 – 909018

AUTONOME PROVINZ BOZEN - SÜDTIROL
SANITÄTSBETRIEB BOZEN
ETHIKKOMITEE
Sitz: Verwaltungsdirektion
L.Böhlerstraße 5 – 39100 Bozen
Tel. 0471 – 908232 Fax 0471 – 909018

COMITATO ETICO DELL'AZIENDA SANITARIA DI BOLZANO

(Istituito ed operante ai sensi del DM 18.3.98 con deliberazione del Direttore Generale nr. 3265 del 9.8.99)

COMPONENTI:

Dr. Mauro ALMICI Dirigente Medico – Direzione Medica dell'Ospedale di Bolzano - Azienda Sanitaria BZ - PRESIDENTE
Dr. Giovanna ZANIRATO Primario del Servizio di Medicina Legale dell'Azienda Sanitaria BZ
Dr. Giorgio RADETTI Dirigente Medico della Divisione di Pediatria dell'Ospedale di Bolzano - Azienda Sanitaria BZ
Dr. Claudio GRAIFF Primario del Servizio di Oncologia Medica dell'Ospedale di Bolzano Azienda Sanitaria BZ
Dr. Peter ZANON Primario del Servizio di Anestesia e Rianimazione 2 dell'Ospedale di Bolzano Azienda Sanitaria BZ
Dr. Christian WIEDERMANN Primario della 2° Divisione Medica dell'Ospedale di Bolzano Azienda Sanitaria BZ
Dr. Josef WIDMANN Dirigente Medico della 1° Divisione Chirurgica dell'Ospedale di Bolzano Azienda Sanitaria BZ
Dr. Adolf PUTZ Direttore Sanitario della casa di cura privata "S. Maria" di Bolzano - Membro non dipendente dalla struttura
Dr. Paul ZELGER Dirigente Farmacista del Servizio Farmaceutico dell'Azienda Sanitaria BZ
Sig.ra Paola NESLER Rappresentante dell'area infermieristica - Capo Sala – Membro non dipendente dalla struttura
Avv. Sonia GASPARRI Responsabile dell'Ufficio Legale dell'Azienda Sanitaria BZ
Prof. Luigi COSTALBANO Rappresentante dell'associazione di volontariato "Tribunale dei diritti del malato" Membro non dipendente dalla struttura
Dr. Antonio FANOLLA Esperto in Statistica – Membro non dipendente dalla struttura
Dr. Renzo CORAZZOLA Dirigente Amministrativo dell'Ospedale di Bolzano – Azienda Sanitaria BZ – SEGRETARIO

PARERE Nr. 38/2006

Bolzano, 30.08.2006

Elenco dei membri partecipanti alla seduta del 29.08.2006

Sono presenti:
Dr. Mauro ALMICI
Dr.ssa Giovanna ZANIRATO
Dr. Giorgio RADETTI
Dr. Claudio GRAIFF
Prof. Christian WIEDERMANN
Dr. Paul ZELGER
Sig.ra Paola NESLER
Prof. Luigi COSTALBANO
Avv. Jutta HUEBER
Dr. Antonio FANOLLA
Rag. Manuela CONVENTO

Sono assenti:
Dr. Diego SIGNORELLO
Dr. Peter ZANON
Dr. Adolf PUTZ

OGGETTO: Richiesta della Divisione di Pneumologia dell'Ospedale di Bolzano di partecipazione allo studio clinico "Comparison of a serum procalcitonin (PRO-CT) guided treatment plan with standard guideline recommended antibiotic treatment plan for patients hospitalized with a diagnosis of exacerbation of COPD" proposto dall'AIFA

**TITOLO DELLO STUDIO:**	Comparison of a serum procalcitonin (PRO-CT) guided treatment plan with standard guideline recommended antibiotic treatment plan for patients hospitalized with a diagnosis of exacerbation of COPD
**FASE DELLA RICERCA:**	
**PROT:**	
**EUDRACT NUMBER:**	
**FARMACO:**	
**IMPRESA FARMACEUTICA:**	
**REPARTO/SERVIZIO:**	Divisione di Pneumologia
**REFERENTE DELLA SPERIMENTAZIONE:**	Dr. Giulio Donazzan

In data 29.08.2006 il Comitato Etico, in osservanza a quanto previsto dal DM 15/7/97

ha preso in esame lo studio in oggetto relativamente a:

☒ **PROTOCOLLO DI STUDIO:**

versione nr. del 24.07.2006

PROVINCIA AUTONOMA DI BOLZANO ALTO ADIGE
AZIENDA SANITARIA DI BOLZANO
COMITATO ETICO
Sede: Direzione Amministrativa
Via L. Böhler 5 – 39100 Bolzano
Tel. 0471 – 908232- Fax 0471 – 909018

AUTONOME PROVINZ BOZEN - SÜDTIROL
SANITÄTSBETRIEB BOZEN
ETHIKKOMITEE
Sitz: Verwaltungsdirektion
L.Böhlerstraße 5 – 39100 Bozen
Tel. 0471 – 908232 Fax 0471 – 909018

☐ **EMENDAMENTO AL PROTOCOLLO**

☐ **INVESTIGATOR'S BROCHURE** nr. del

☒ **RAPPRESENTAZIONE SCHEMATICA DELLO STUDIO** farmaco versione nr. del

☐ **PARERE UNICO ALL'EFFETTUAZIONE DELLO STUDIO**
il comitato etico del centro coordinatore dello studio non ha ancora espresso parere unico

☒ **MODULO INFORMATIVO PER IL PAZIENTE E CONSENSO INFORMATO** versione nr. del

☐ **MODULO INFORMATIVO PER IL MEDICO DI FAMIGLIA** versione nr. del

☐ **POLIZZA ASSICURATIVA:** compagnia assicurativo, validità dal al - polizza nr. massimale per sinistro e per anno

☒ **CONVENZIONE ECONOMICA**

ha espresso il seguente parere:

☐ **APPROVATO**
☒ **APPROVATO CON NOTA**
☐ **APPROVATO A CONDIZIONE CHE VENGANO SODDISFATTE LE RICHIESTE DI SEGUITO INDICATE**
☐ **NON APPROVATO PER LE MOTIVAZIONI INDICATE**
☐ **SOSPESO IN ATTESA DEI CHIARIMENTI RICHIESTI**
☐ **PRESO ATTO DELL'EFFETTUAZIONE DELLO STUDIO**

Il presente parere favorevole è subordinato al parere unico che sarà espresso dal Comitato Etico del centro coordinatore.

LA SEGRETARIA SUPPLENTE
(Rag. Manuela Convento)
M Convento

IL PRESIDENTE
(Dr. Mauro Almici)

PROVINCIA AUTONOMA DI BOLZANO - ALTO ADIGE
AZIENDA SANITARIA DI BOLZANO
COMITATO ETICO

AUTONOME PROVINZ BOZEN - SÜDTIROL
SANITÄTSBETRIEB BOZEN
ETHIKKOMITEE

Estratto verbale della 20° riunione del Comitato Etico dell'Azienda Sanitaria di Bolzano
(istituito ai sensi della delibera nr. 1744/04)

Il giorno 12.12.2006 - ad ore 14.30 presso la sala riunioni del Servizio di Ingegneria Clinica dell'ospedale di Bolzano si riunisce il Comitato Etico.

Sono presenti:

Dr. Mauro ALMICI	Presidente
Dr.ssa Giovanna ZANIRATO	Membro
Prof. Christian WIEDERMANN	Membro
Dr.ssa Judith STOCKER	Membro (sostituta del Dr. Graiff)
Dr. Diego SIGNORELLO	Membro
Dr. Giorgio RADETTI	Membro
Dr. Adolf PUTZ	Membro
Dr.ssa Alicia TAVELLA	Membro (sostituta del Dr. Zelger)
Dr. Antonio FANOLLA	Membro
Prof. Luigi COSTALBANO	Membro
Sig.ra Paola NESLER	Membro
Rag. Manuela CONVENTO	Segretaria

Sono assenti giustificati: Dr. Peter Zanon, Prof. Christian Wiedermann Avv. Jutta Hueber

- **OMISSIS –**

Punto 5 dell'o.d.g. (seduta del 12.12.2006)

Divisione di Pneumologia – Studio clinico "Confronto fra un regime terapeutico basato sui livelli sierici di procalcitonina rispetto ad un regime terapeutico standard in pazienti ospedalizzati per riacutizzazione di broncopneumopatia cronica ostruttiva"
Emendamento al protocollo di studio

Il presidente legge ai presenti la nota pervenuta dal referente dello studio, Dr. Giulio Donazzan, dalla quale si evince che l'emendamento riguarda le seguenti modifiche al protocollo di studio:
- l'indirizzo errato di uno dei centri coinvolti nello studio
- l'esplicitazione dei criteri internazionali adottati per alcune definizioni e la citazione delle linee guida per la diagnosi
- una più accurata specificazione dei criteri di monitoraggio degli eventi avversi.

Sono stati inoltre specificati in maniera più corretta ed attenta:
- gli obiettivi secondari dove il gruppo di confronto deve presentare sicuramente lo stesso tipo di patologia, cioè la riacutizzazione infettiva di bronchite
- per quanto concerne i trattamenti di confronto viene specificato più dettagliatamente come i pazienti verranno inseriti nei bracci dello studio
- nei criteri di inclusione viene specificato meglio un criterio di valutazione funzionale respiratoria
- sempre nei criteri di inclusione viene fatto preciso riferimento ai criteri Anthonisen per la valutazione clinica.

Il Comitato dopo aver preso atto di quanto comunicato ed accertato che l'emendamento non comporta sostanziali modifiche all'impianto iniziale dello studio, approva l'emendamento al protocollo di studio – versione 2 settembre 2006.

- OMISSIS -

COMITATO ETICO - COMPOSIZIONE AL 04.10.2005

MEMBRI EFFETTIVI	Qualifica	MEMBRI SUPPLENTI	Qualifica
**Dr. Flavio GIRARDI**	Dirigente Medico di II° livello del Servizio Ospedaliero – Azienda Sanitaria di BZ PRESIDENTE	**Dr. Mauro ALMICI**	Dirigente Medico di I° livello della Direzione Sanitaria – VICE PRESIDENTE
**Dr. Giovanna ZANIRATO**	Dirigente Medico di II° livello – Responsabile del Servizio di Medicina Legale dell' Azienda Sanitaria di BZ	**Dr. Maria Cristina SALERNO**	Dirigente Medico di I° livello del Servizio di Medicina Legale dell'Azienda Sanitaria di BZ
**Dr. Giorgio RADETTI**	Medico della Divisione di Pediatria dell'Ospedale di Bolzanc – Azienda Sanitaria di BZ	**Dr. Hubert MESSNER**	Medico della Divisione di Pediatria dell'Ospedale di Bolzanc – Azienda Sanitaria di BZ
**Dr. Claudio GRAIFF**	Primario del Servizio di Oncologia dell'Ospedale di Bolzanc – Azienda Sanitaria di BZ	**Dr.ssa Judith STOCKER**	Medico del Servizio di Oncologia dell'Ospedale di Bolzanc – Azienda Sanitaria di BZ
**Dr. Peter ZANON**	Primario del Servizio di Anestesia e Rianimazione 1 dell'ospedale di Bolzano – Azienda Sanitaria di BZ	**Dr. Giorgio DE GIORGI**	Medico del Servizio di Anestesia e Rianimazione II dell'ospedale di Bolzano – Azienda Sanitaria di BZ
**Dr. Christian WIEDERMANN**	Primario della 2° Divisione Medica dell'ospedale di Bolzano – Azienda Sanitaria di BZ	**Dr. Werner Günther RAUHE**	Medico della Divisione di Cardiologia dell'ospedale di Bolzano – Azienda Sanitaria di Bolzano
**Dr. Josef WIDMANN**	Medico della 1° Divisione Chirurgica dell'ospedale di Bolzano – Azienda Sanitaria di Bolzano	**Dr. Diego SIGNORELLO**	Medico della Divisione di Urologia dell'ospedale di Bolzano – Azienda Sanitaria di Bolzano
**Dr. Adolf PUTZ**	Direttore Sanitario della casa di cura privata "S. Maria" di Bolzano Membro non dipendente dalla struttura	**Dr. Paolo BONVICINI**	Direttore Sanitario della casa di cura privata "Bonvicini" – Bolzano Membro non dipendente dalla struttura
**Dr. Paul ZELGER**	Dirigente Farmacista di I° livello del Servizio Farmaceutico dell'Azienda Sanitaria di BZ	**Dr.ssa Alicia TAVELLA**	Dirigente Farmacista di I° livello del Servizio Farmaceutico dell'Azienda Sanitaria di BZ
**Sig.ra Paola NESLER**	Rappresentante dell'area infermieristica Capo Sala – Membro non dipendente dalla struttura	**Sig. Walter FABBRICOTTI**	Rappresentante dell'area infermieristica Infermiere c/o Distretto di Chiusa dell'Azienda Sanitaria di Bressanone
**Avv. Sonia GASPARRI**	Rappresentante dell'Ufficio Legale dell'Azienda Sanitaria di Bolzano	**Avv. Jutta HUEBER**	Rappresentante dell'Ufficio Legale dell'Azienda Sanitaria di Bolzano
**Prof. Luigi COSTALBANO**	Rappresentante dell'associazione di volontariato "Tribunale dei diritti del malato" Membro non dipendente dalla struttura	**Dr.ssa Simonetta MANCA**	Rappresentante dell'associazione di volontariato "Tribunale dei diritti del malato" Membro non dipendente dalla struttura
**Dr. Antonio FANOLLA**	Statistico – funzionario dell'osservatorio epidemiologico provinciale Membro non dipendente dalla struttura	**Dr.ssa Carla MELANI**	Statistica – Responsabile dell'osservatorio epidemiologico provinciale Membro non dipendente dalla struttura
**Dr. Renzo CORAZZOLA**	Dirigente Amministrativo dell'Ospedale di Bolzano – AZ. Sanit. BZ – SEGRETARIO	**Rag. Manuela CONVENTO**	Assistente Amministrativo c/o Direzione Amministrativa dell'Ospedale di Bolzano – AZ.Sanitaria BZ – SEGRETARIA SUPPL.

FONDAZIONE SALVATORE MAUGERI
CLINICA DEL LAVORO E DELLA RIABILITAZIONE
D.P.R. 991 DEL 15-6-1965
I.R.C.C.S.

Sede legale e amministrativa
Via Salvatore Maugeri 4, 27100 Pavia Ital
Tel. 0382 592504 Fax 0382 592576

Istituto Scientifico di Cassano delle Murge (BA)

Divisione di Pneumologia

Via per Mercadante Km.2
70020 Cassano delle Murge (BA)
Tel. 080 7814111

Istituti Scientifici:

PAVIA 27100
- Sede di Via Salvatore Maugeri 10
- Sede di Via Palestro 26
Tel. 0382 5921
Fax 0382 592081
- Sede di Via Boezio 28
Tel. 0382 5931
Fax 0382 593081

MILANO
- Sede di Via Clefi 9 (cap 20146)
Tel. 02 43069511
Fax 02 43069529
- Sede di Via Camaldoli 64 (cap 20138)
Tel. 02 507259
Fax 02 50725201

MONTESCANO
27040 (PV)
Via per Montescano
Tel. 0385 2471
Fax 0385 61386

LISSONE
20035 (MI)
Via Mons. Bernasconi 16
Tel. 039 4657235
Fax 039 4657234

LUMEZZANE
25066 (BS)
Via Mazzini 129
Tel. 030 8253011
Fax 030 8920262

TRADATE
21049 (VA)
Via Roncaccio 16
Tel. 0331 829111

Da: Dr. Mauro Carone
Divisione di Pneumolgia Riabilitativa – Ist. Scientifico di Cassano Murge (BA)

a : Comitato Etico Centrale – Fondazione Salvatore Maugeri - PAVIA

p.c. : Direzione Tecnico scientifica
Fondazione "Salvatore Maugeri" - Cassano delle Murge (BA)

Oggetto: sperimentazione clinica "COMPARISON OF A SERUM PROCALCITONIN (PRO-CT) GUIDED TREATMENT PLAN WITH THE STANDARD GUIDELINE RECOMMENDED ANTIBIOTIC TREATMENT PLAN FOR PATIENTS HOSPITALIZED WITH A DIAGNOSIS OF EXACERBATION OF COPD"

Con la presente si comunica che in data 28 ottobre 2011 è stata effettuata la visita di chiusura del centro per il termine della sperimentazione clinica in oggetto, già autorizzata da codesto Comitato nella seduta dell'8 gennaio 2007 (N. Archivio CEC 451).

Di seguito viene riportata la situazione aggiornata:

- Numero totale pazienti screenati : 17
- N° totale pazienti randomizzati: 17
- Nà Pazienti Screening failure: 00
- N° totale pazienti completati :12
- N° totale di pazienti usciti prematuramente per ritiro consenso : 05
- N° totale decessi:00
- N° totale eventi avversi seri occorsi: 00

La documentazione della sperimentazione clinica verrà conservata presso la Fondazione Salvatore Maugeri di Cassano delle Murge (BA) per 15 anni dalla chiusura della stessa secondo la normativa vigente.
RingraziandoVi per l'attenzione, rimaniamo a disposizione per ulteriori chiarimenti.
Cordiali saluti,

Dr. Mauro Carone.
Principal Investigator

Cassano delle Murge, 28.10.2011

COMITATO ETICO DELL'AZIENDA OSPEDALIERA
DI RILIEVO NAZIONALE E DI ALTA SPECIALIZZAZIONE
"Garibaldi, S.Luigi-Currò,Ascoli-Tomaselli"
Piazza S. Maria di Gesù n. 5 - 95123 CATANIA

Catania, 29 DIC 2006
Tel. 095/7594943-fax 095-7594817
e-mail: raimondovinci@aliceposta.it

N° di prot. 1415/CE .... Allegati n.°....................

OGGETTO: Studio: Confronto di uno schema di trattamento antibiotico deciso in accordo ai livelli sierici di Procalcitonina rispetto al trattamento standard in pazienti ricoverati per riacutizzazione di broncopneumopatia cronica ostruttiva (BPCO)".

Spett.le Dipartimento Integrato di
Oncologia ed Ematologia
Università degli Studi di Modena e
Reggio Emilia
Via Del Pozzo n. 71
<u>41100 M O D E N A</u>

Prof./Dott. Nunzio Crimi
U.O. Allergologia
<u>P.O. ASCOLI - TOMASELLI</u>

Al Ministero della Salute
Dipartimento per la valutazione dei medicinali
e la Farmacovigilanza
Ufficio Sperimentazione Clinica
Viale della Civiltà Romana, 7
<u>00144 ROMA EUR</u>

In risposta alla richiesta avanzata da codesto Dipartimento, concernente lo studio in oggetto, si comunica che il Comitato Etico di questa Azienda, riunitosi in data 27/11/2006 (Verbale n. 9/CE/06), ha espresso il parere di competenza che, in allegato, si trasmette per gli adempimenti di rispettiva pertinenza in uno alla lista dei membri del Collegio e dei relativi documenti esaminati.

Il Presidente
del Comitato Etico
(Dott. Salvatore Giuffrida)

COMITATO ETICO DELL'AZIENDA OSPEDALIERA
DI RILIEVO NAZIONALE E DI ALTA SPECIALIZZAZIONE
"Garibaldi, S.Luigi-Currò, Ascoli-Tomaselli"
Piazza S. Maria di Gesù n. 5 – 95123 CATANIA

Tel.095/7594943- Fax 095/7594817

OGGETTO: Parere.

Elenco dei membri partecipanti alla seduta del 27/11/2006 (Verbale n. 9/CE/06)

P	A	Dott.	Salvatore Giuffrida	Direttore Sanitario Azienda
P	A	Dott.	Gaetano Siscaro	Magistrato
P	A	Avv.	Ilio Bertocchi	Avvocato del Foro di Catania
P	A	Prof.	Giuseppe Di Maria	Dir. Med. 2° Liv. Azienda
P	A	Prof.	Filippo Drago	Farmacologo Università di Catania
P	A	D.ssa	Maria Rosa Calcagno	Dir. Farmacista Azienda
P	A	Dott.	Vito Schillaci	Dir. Med. 2° Liv. Azienda
P	A	Prof.ssa	Santa Salvo	Biostatistica dell'Università di Catania

OGGETTO: Studio: Confronto di uno schema di trattamento antibiotico deciso in accordo ai livelli sierici di Procalcitonina rispetto al trattamento standard in pazienti ricoverati per riacutizzazione di broncopneumopatia cronica ostruttiva (BPCO)".

ELENCO DOCUMENTI ESAMINATI:

1. Protocollo
2. Sinossi del protocollo
3. Modello di consenso informato
4. Bozza di Convenzione.

Il Comitato Etico, esaminata la documentazione sopra indicata e sulla base delle normative vigenti in materia, esprime PARERE FAVOREVOLE alla conduzione dello studio di che trattasi - da attuarsi sotto la responsabilità del prof. Nunzio Crimi presso l'U.O. di Allergologia del P.O. Ascoli - Tomaselli con le seguenti prescrizioni formali, al cui solo soddisfacimento potrà darsi corso senza ulteriore valutazione da parte del Collegio:

- Acquisizione formale lettera di richiesta di valutazione da parte dello Sperimentatore;
- In considerazione del fatto che nello studio è prevista l'ospedalizzazione per tre giorni, lo Sperimentatore deve indicare presso quale U.O. intende avviare la sperimentazione in questione.

Si dichiara che questo Comitato Etico è organizzato ed opera nel rispetto delle norme di Buona Pratica Clinica (GCP_ICH) e secondo la normativa vigente sulle sperimentazioni cliniche e sull'istituzione dei Comitati Etici.

Si ritiene qualificato lo Sperimentatore ed adeguate le strutture dove verrà svolta la ricerca.

Si invita altresì lo Sperimentatore a voler trasmettere relazione finale alla conclusione dello studio.

Il Segretario
del Comitato Etico
(d.ssa Gaetana Piccolo)

Il Presidente
del Comitato Etico
(dott. Salvatore Giuffrida)

ARCISPEDALE SANT'ANNA
AZIENDA OSPEDALIERO UNIVERSITARIA DI FERRARA
Servizio Sanitario Nazionale Regione Emilia-Romagna

Ferrara, 24 ottobre 2006

"Confronto fra un regime terapeutico basato sui livelli sierici di procalcitonina rispetto ad un regime terapeutico standard in pazienti ospedalizzati per riacutizzazione di broncopneumopatia cronica ostruttiva"

**Proponente responsabile**:	Prof. A. PAPI, Unità Operativa di Pneumologia, Azienda Ospedaliera Universitaria Arcispedale "S. Anna", Ferrara.
**Staff operativo**:	/ /
**Ditta/sponsor**:	/ /
**Codice identificativo del piano clinico generale**:	/ /
**Specialità medicinale (nome o sigla):**	/ /
**Principio attivo:**	amoxicillina + acido clavulanico o chinolonici
**Codice CAS (ove disponibile)**:	/ /
**Classe farmacologica di appartenenza**:	antibiotici
**Codice ATC proposto (secondo codifica OMS)**:	J01 CR, J01 MA
**Codice ICD**:	/ /
**Fase della sperimentazione clinica**:	IV
**Indicazione proposta**:	Si tratta di uno studio multicentrico, prospettico, randomizzato e controllato , eseguito in singolo cieco, che ha lo scopo di confrontare due regimi terapeutici (come da linee guida internazionali) a base dello stesso tipo di antibiotici, ma somministrati per tempi diversi, per il trattamento di pazienti con BPCO (broncopneumopatia cronica ostruttiva) ospedalizzati per riacutizzazione infettiva di malattia.
**Forma farmaceutica**:	compresse o fiale
**Via di somministrazione**:	per os oppure endovena

Durata dello studio: lo studio prevede la durata di 24 mesi.

Posologia: come da linee guida

Schema dello studio: lo studio prevede due bracci :
1) un braccio sperimentale che riceverà una terapia antibiotica somministrata per 3 giorni (terapia minima consigliata da linee guida) e poi interrotta/proseguita sulla base dei livelli sierici di procalcitonina;
2) un braccio di controllo che riceverà una terapia antibiotica somministrato per un totale di 10 giorni (terapia massima consigliata).

Eventuale terapia concomitante: //

AIC in Italia: //
all'estero: //

Indicazioni all'AIC, posologia, vie di somministrazione e forme farmaceutiche autorizzate: //

Precedenti approvazioni/autorizzazioni alla sperimentazione per la stessa indicazione proposta: *Parere Unico rilasciato dal Comitato Etico dell'Azienda Ospedaliero-Universitaria di Modena in data 5 settembre 2006; approvazione da parte dell'Agenzia Italiana del Farmaco (AIFA) in data 24 marzo 2006.*

Obiettivi dello studio:
Primario
- Determinare se la terapia antibiotica possa essere interrotta senza rischi dopo 3 giorni o debba essere continuata per 10 giorni sulla base di un regime terapeutico guidato dalla procalcitonina, in pazienti ospedalizzati per riacutizzazioni di BPCO richiedente trattamento antibiotico e associata ad aumento della dispnea e del volume e purulenza dell'essudato;
- Lo studio è disegnato per valutare la non inferiorità del trattamento guidato dalla procalcitonina rispetto al trattamento standard di 10 giorni raccomandato dalle linee guida. Tutti i pazienti riceveranno 3 giorni di terapia antibiotica indipendentemente dal braccio di randomizzazione, come indicato dalle linee guida.

Secondari
- Stimare i costi diretti ed indiretti dei pazienti ospedalizzati per riacutizzazione di BPCO ed il rapporto costo/beneficio del trattamento antibiotico guidato dalla pro-ct.
- Valutare se 10 giorni di terapia antibiotica instaurati sulla base dei valori di pro-ct risultano più efficaci rispetto ad una terapia antibiotica di analoga durata stabilita secondo i criteri indicati dalle linee guida in pazienti affetti da riacutizzazione infettiva di BPCO.
- Confrontare il valore predittivo dei criteri clinici sopraenunciati con biomarcatori tradizionali, quali la PCR, la conta dei leucociti, la febbre, ecc. e nuovi, quali peptide natriuretico, le proteine di fase acuta ed i proormoni.
- Valutare l'incidenza di effetti collaterali legati all'uso degli antibiotici.
- Determinare la relazione esistente fra i livelli sierici di pro-ct con la specie batterica isolata nell'espettorato, la carica virale e l'eziologia della riacutizzazione.
- Stimare attraverso il dosaggio della pro-ct sierica il numero di riacutizzazione di BPCO definite sulla base dei criteri clinici sopraindicati.
- Data la mancanza di specificità dei sintomi determinati dalle riacutizzazioni di BPCO,

valutare l'eterogeneicità di tali riacutizzazioni, in quanto esse possono essere determinate da altre patologie, quali lo scompenso cardiaco, l'embolia polmonare, ecc.

Tipologia dei soggetti da arruolare (specificare se pazienti o volontari sani): pazienti di entrambi i sessi, di età maggiore di 18 anni, con diagnosi di BPCO, in fase di riacutizzazione.

Numero dei soggetti da arruolare: si prevede l'arruolamento di 400 soggetti, 200 per ogni braccio di studio.

Informazione al candidato: mediante scheda informativa, nella quale si riportano notizie sulla natura, durata e scopo dello studio, nonché il rapporto rischio/beneficio.

L'informazione del paziente, in virtù della propedeuticità di tale fase, dovrà essere fornita in un momento formalmente distinto dal recepimento del consenso.

Si raccomanda che l'avvenuta informazione venga formalizzata su scheda personale del paziente (in alternativa su modulo che ne faccia parte integrante), riportando contestualmente data e firma del medico sperimentatore e dell'arruolando stesso.

Recepimento del consenso: mediante apposito modulo;

Si raccomanda che il recepimento del consenso/dissenso avvenga in un momento formalmente distinto dalla fase informativa e ad essa successivo e venga formalizzato su scheda personale del paziente (oppure su modulo che ne faccia parte integrante).

Criteri di inclusione/esclusione:
criteri di inclusione:

- pazienti di entrambi i sessi, di età > 18 anni;
- pazienti con diagnosi di BPCO secondo le linee guida GOLD: FEV1/VC < 70% con FEV1 < 80% del predetto. Nel caso la diagnosi di BPCO non possa essere desunta da esami spirometrici precedenti, si eseguirà un esame spirometrico entro 3 giorni dal ricovero e si porrà diagnosi di nuovo caso di BPCO in presenza dei 3 seguenti criteri:
    - FEV1/VC < 70% con FEV1 < 80% del predetto;
    - storia di abitudine tabagica;
    - esclusione di diagnosi di asma.

  Al termine dello studio verrà eseguito un ulteriore esame spirometrico per confermare la diagnosi di nuovo caso di BPCO fatta all'ingresso.
- Pazienti con diagnosi di BPCO riacutizzata:
    1. dispnea acuta e/o tosse associate ad aumento del volume e viraggio del colore dell'espettorato (Anthonisen's criteria) in assenza di altre malattie polmonari o cardiache;
    2. necessità di terapia antibiotica secondo le linee guida GOLD 2005;
    3. necessità di ospedalizzazione.

criteri di esclusione:

- pazienti di sesso femminile in gravidanza o allattamento;
- pazienti con diagnosi di asma;

- pazienti con malattie concomitanti instabili a carico di apparato cardiovascolare, reni, fegato, apparato digerente, sistema nervoso, sistema endocrino, metabolismo, apparato muscolo scheletrico, tumori, malattie respiratorie o altre malattie cliniche rilevanti;
- pazienti con anomalie di laboratorio indicanti condizioni cliniche instabili;
- pazienti in cui non si può garantire la sopravvivenza ad 1 anno.

Sorveglianza clinica:
Lo studio prevede un totale di 7 visite, con randomizzazione alla visita 3.
Alla visita 1 (giorno 0, ospedalizzazione) saranno effettuati: raccolta dell'anamnesi, esame fisico, valutazione dei segni vitali e della temperatura corporea, valutazione di terapie concomitanti, radiografia del torace, spirometria, raccolta di escreato, ECG, somministrazione di questionario sulla qualità della vita e sulla farmaco-economia, prelievo ematico (per analisi ematochimiche, valutazione della procalcitonina, Proteina C Reattiva, amiloide sierica, RANTES -Regulated on Activation Normal T Expressed and Secreted-, PTX3 –pentraxina-, IL 1, emogas analisi),valutazione della compliance al trattamento ed eventuali eventi avversi.
Alla visita 2 e 3 saranno nuovamente valutati: esame fisico, valutazione dei segni vitali e della temperatura corporea, valutazione di terapie concomitanti, valutazione della procalcitonina, Proteina C Reattiva, amiloide sierica, RANTES, emogas analisi, valutazione della compliance al trattamento ed eventuali eventi avversi.
Alle successive visite saranno valutati: esame fisico (visita 4, 5, 6, 7), valutazione dei segni vitali e della temperatura corporea (visita 4, 5, 6, 7), valutazione di terapie concomitanti (visita 4, 5, 6, 7), spirometria (visita 4, 5, 6, 7), raccolta di escreato (4, 5, 7), somministrazione di questionario sulla qualità della vita e sulla farmaco-economia (visita 4, 5, 6, 7), prelievo ematico (per analisi ematochimiche, valutazione della procalcitonina, Proteina C Reattiva, amiloide sierica, RANTES - Regulated on Activation Normal T Expressed and Secreted-, PTX3 –pentraxina-, emogas analisi), valutazione della compliance al trattamento ed eventuali eventi avversi (visita 4, 5, 6, 7).

Modalità di sospensione: è prevista l'interruzione dello studio qualora si verifichino eventi avversi, su decisione del medico sperimentatore e, comunque, per libera scelta e disposizione dell'arruolato.

Il Comitato Etico esprime parere favorevole allo studio proposto, ove siano soddisfatti i prerequisiti etici nei termini innanzi richiesti.

Si dà atto che il Comitato Etico ha preso visione della seguente documentazione:
- *n.1 copia di Protocollo di studio, versione 2 settembre 2006;*
- *n.1 copia di Sinossi del protocollo;*
- *n.1 copia di Modulo di Informazione per il paziente;*
- *n.1 copia di Modulo di Consenso;*
- *n.1 copia di Parere Unico rilasciato da Comitato Etico dell'Azienda Ospedaliero-Universitaria di Modena in data 5/09/06;*
- *n.1 copia di Lista dei Centri partecipanti Progetto AIFA;*
- *n.1 copia di Case Report Form;*
- *n.1 copia di Scheda di segnalazione.*

Il Segretario

Francesca Palara

Valentina Ruggieri

Da: Petilli Silvia [petillis@aou-careggi.toscana.it]
Inviato: martedì 25 marzo 2014 17.09
A: Valentina Ruggieri
Cc: Alessia VERDURI; segrscient.cesf@pharm.unifi.it
Oggetto: R: Richiesta num approvazione etica centro satellite

Gent.ma Dr.ssa Ruggirei, lo studio AIFA prot. FARM58J2XH corrisponde alla ns. pratica n. 543/2006 e fu approvato dal Comitato Etico della Azienda Careggi per il centro del Prof. Massimo Pistolesi (Fisiopatologia Respiratoria) nella seduta del 7 novembre 2006

Cordiali saluti

Silvia Petilli
Segreteria Comitato Etico Area Vasta Centro
Azienda Ospedaliero-Universitaria Careggi
Padiglione 3
Largo Brambilla, 3
50134 Firenze
Tel 055 794.7396
Fax 055 794.9624

Per maggiori informazioni sulle sperimentazioni cliniche in corso in AOU Careggi, accedere al seguente link: <http://www.aou-careggi.toscana.it/internet/index.php?option=com_content&task=view&id=976&Itemid=216>

Da: Valentina Ruggieri [mailto:valentina.ruggieri@unimore.it]
Inviato: martedì 25 marzo 2014 16.50
A: Petilli Silvia; segrscient.cesf@pharm.unifi.it
Cc: 'Alessia VERDURI'
Oggetto: Richiesta num approvazione etica centro satellite

Spett.le Comitato Etico,

la presente e-mail, per chiedere il vostro numero di riferimento (pratica, protocollo, procedimento…) di approvazione e data della seduta del seguente studio AIFA FARM58J2XH: "COMPARISON OF A SERUM PROCALCITONIN (PRO-CT) GUIDED TREATMENT PLAN WITH THE STANDARD GUIDELINE RECOMMENDED ANTIBIOTIC TREATMENT PLAN FOR PATIENTS HOSPITALIZED WITH A DIAGNOSIS OF EXACERBATION OF COPD".
Si allega parere favorevole del centro coordinatore Modena (Prof Leonardo Fabbri).

In attesa di un vostro cortese riscontro, porgo cordiali saluti.

Valentina Ruggieri

Dott.ssa Valentina Ruggieri

Clinical Research Coordinator
Clinica Malattie Apparato Respiratorio

Policlinico di Modena
Via del Pozzo 71,
I-41124 MODENA

Tel. 0039 059 422 5762
Fax 0039 059 422 3113
email valentina.ruggieri@unimore.it
