# Supplementary material for: Antibiotic Treatment of Severe Exacerbations of Chronic Obstructive Pulmonary Disease with Procalcitonin: A Randomized Noninferiority Trial
Source: PLoS One. 2015 Mar 11;10(3):e0118241. doi: 10.1371/journal.pone.0118241 (PMC4356612; doi:10.1371/journal.pone.0118241)
Supplement: S1 Table — (DOCX) [file pone.0118241.s006.docx]

**S1 Table: All data of primary and secondary outcomes and adverse events for all**

**patients at all visits**

| SUPPORTING INFORMATION | | | | | | | | | | | | | | | | | | | | | | | | | | | |  |
| --- | --- | --- | --- | --- | --- | --- | --- | --- | --- | --- | --- | --- | --- | --- | --- | --- | --- | --- | --- | --- | --- | --- | --- | --- | --- | --- | --- | --- |
|  |  | Primary  outcome  (ECOPD) | | | Secondary outcomes | | | | | | | | | | | | | | | | | | | | | | | Adverse  events |
| ID | Arm |  |  |  | Subsequent use  of antibiotic  for the  treatment  of ECOPD | Clinical  success | Hospital  Readmission  for ECOPD | | | Hospital  Readmission  for any  cause | | | Need  for ICU  stay | | | Respiratory  failure | | | Death | | Change in  lung function  (ΔFEV1, liter) | | | Change in  lung function  (ΔFEV1, %pred) | | | Lenght of  hospital  stay |  |
|  |  | 30d | 90d | 180d |  |  | 30d | 90d | 180d | 30d | 90d | 180d | 30d | 90d | 180d | 30d | 90d | 180d |  |  | 30d | 90d | 180d | 30d | 90d | 180d |  |  |
| 1 | STD | 0 | 0 | 0 | 0 | 0 | 0 | 0 | 0 | 0 | 0 | 0 | 0 | 0 | 0 |  |  |  |  |  |  |  |  |  |  |  |  |  |
| 2 | PROCT 10d | 0 | 0 | 0 | 0 | 1 | 0 | 0 | 0 | 0 | 0 | 0 | 0 | 0 | 0 | 1 | 1 | 1 |  |  | 0.44 | 0.41 | 0.32 | 19 | 18 | 14 | 7 |  |
| 3 | PROCT 3d | 0 | 0 | 0 | 0 | 1 | 0 | 0 | 0 | 0 | 0 | 0 | 0 | 0 | 0 |  |  |  |  |  | 0.14 |  | 0.28 | 10 |  | 18 | 6 |  |
| 4 | PROCT 10d | 0 | 1 | 0 | 1 | 0 | 0 | 1 | 0 | 0 | 1 | 0 | 0 | 0 | 0 |  | 1 |  |  |  | -0.02 | 0.26 | 0.01 | -1 | 8 | 0 | 7 |  |
| 5 | STD | 0 | 0 | 0 | 1 | 0 | 0 | 0 | 0 | 0 | 0 | 0 | 0 | 0 | 0 | 1 |  |  |  |  | 0.19 | -0.11 | 0.05 | 11 | -6 | 3 | 7 |  |
| 6 | STD | 0 | 0 | 0 | 0 | 1 | 0 | 0 | 0 | 0 | 0 | 0 | 0 | 0 | 0 |  |  |  |  |  | 0.7 | 0.66 | 0.36 | 27 | 26 | 24 | 7 |  |
| 7 | STD | 1 | 1 | 0 | 1 | 0 | 0 | 0 | 0 | 0 | 0 | 0 | 0 | 0 |  |  |  |  |  |  | 0.22 | 0.03 | 0.06 | 9 | 1 | 3 | 5 |  |
| 8 | PROCT 10d | 0 | 0 | 0 | 0 | 0 | 0 | 0 | 0 | 0 | 0 | 0 |  |  |  |  |  |  |  |  |  |  |  |  |  |  | 8 |  |
| 9 | PROCT 10d | 1 | 0 | 0 | 1 | 1 | 0 | 0 | 0 | 0 | 0 | 0 | 0 |  |  |  |  |  |  |  | 0.17 |  |  | 11 |  |  | 11 |  |
| 10 | STD | 0 | 0 | 0 | 0 | 0 | 0 | 0 | 0 | 0 | 0 | 0 | 0 | 0 |  | 1 |  | 1 |  |  | 0.49 | 0.54 | 0.24 | 17 | 15 | 7 | 7 |  |
| 11 | STD | 0 | 1 | 0 | 1 |  | 0 | 0 | 0 | 0 | 0 | 0 | 0 | 0 | 0 |  |  |  |  |  | 0.02 | -0.31 | -0.18 | 1 | -10 | -8 | 9 |  |
| 12 | STD | 0 | 0 | 0 | 0 |  | 0 | 0 | 0 | 0 | 0 | 0 | 0 | 0 | 0 |  |  |  |  |  | -0.07 | 0.14 | 0.3 | 0 | 8 | 10 | 11 |  |
| 13 | STD | 0 | 1 | 0 | 0 |  | 0 | 0 | 0 | 0 | 0 | 0 | 0 | 0 | 0 |  |  |  |  |  | 0.2 | 0.03 | 0.1 | 12 | 2 | 6 | 9 |  |
| 14 | STD | 0 | 0 | 0 | 0 |  | 0 | 0 | 0 | 0 | 0 | 0 |  |  |  |  |  |  |  |  | 0.09 |  |  | 3 |  |  | 7 |  |
| 15 | PROCT 10d | 1 | 1 | 1 | 1 | 1 | 0 | 0 | 1 | 0 | 0 | 1 | 0 | 0 | 0 |  |  |  |  |  | -0.03 | -0.02 | -0.12 | -1 | -1 | -4 | 6 |  |
| 16 | PROCT 3d | 1 | 0 | 0 | 1 |  | 0 | 0 | 0 | 0 | 0 | 0 | 0 | 0 | 0 |  |  |  |  |  | 0.17 | 0.57 | 0.16 | 8 | 27 | 9 | 8 |  |
| 17 | PROCT 3d | 0 | 0 | 0 | 0 |  | 0 | 0 | 0 | 0 | 0 | 0 | 0 | 0 | 0 |  |  |  |  |  | -0.14 | -0.11 | -0.03 | -6 | -5 | -1 | 7 |  |
| 18 | PROCT 3d | 0 | 0 | 0 | 0 |  | 0 | 0 | 0 | 0 | 0 | 0 | 0 |  |  |  |  |  |  |  | 0.17 |  |  | 6 |  |  | 12 |  |
| 19 | PROCT 3d | 0 | 0 | 0 | 0 |  | 0 | 0 | 0 | 0 | 0 | 0 | 0 | 0 | 0 |  |  |  |  |  | -0.31 | -0.12 | -0.22 | -12 | -5 | -8 | 12 |  |
| 20 | STD | 1 | 1 | 0 | 1 |  | 0 | 0 | 0 | 0 | 0 | 0 | 0 | 0 | 0 |  | 1 |  |  |  | -0.08 | -0.15 | -0.14 | -3 | -5 | -5 | 8 |  |
| 21 | STD | 0 | 0 | 0 | 0 | 1 | 0 | 0 | 0 | 0 | 1 | 0 | 0 | 0 | 0 | 1 |  | 1 |  |  | 0.53 | 0.52 | 0.58 | 21 | 21 | 23 | 8 |  |
| 22 | STD | 0 | 0 | 0 | 0 | 1 | 0 | 0 | 0 | 0 | 0 | 0 | 0 | 0 | 0 |  |  |  |  |  | 0.34 | 0.31 | 0.37 | 12 | 11 | 13 | 6 |  |
| 23 | PROCT 3d | 0 | 0 | 0 | 0 | 1 | 0 | 0 | 0 | 0 | 0 | 0 | 0 | 0 |  |  | 1 |  |  |  | 0.22 | 0.24 |  | 8 | 7 |  | 4 |  |
| 24 | PROCT 3d | 0 | 0 | 1 | 0 | 1 | 0 | 0 | 1 | 0 | 1 | 1 | 0 | 0 | 0 |  |  | 1 |  |  | 0.02 | 0.2 | -0.28 | 1 | 8 | -12 | 6 |  |
| 25 | PROCT 3d | 0 | 0 | 0 | 0 | 1 | 0 | 0 | 0 | 0 | 0 | 0 | 0 | 0 | 0 |  |  |  |  |  | 0.03 | 0.1 | 0.2 | 2 | 5 | 10 | 4 |  |
| 26 | STD | 1 | 0 | 0 | 0 | 1 | 1 | 0 | 0 | 1 | 0 | 0 | 0 |  |  |  |  |  | 1 | | -0.02 |  |  | -1 |  |  | 5 |  |
| 27 | STD | 0 | 0 | 0 | 0 | 1 | 0 | 0 | 0 | 0 | 0 | 0 | 0 | 0 | 0 | 1 | 1 | 1 |  |  | 0.12 | 0.06 | 0.15 | 3 | 1 | 4 | 7 |  |
| 28 | STD | 0 | 0 | 0 | 0 | 1 | 0 | 0 | 0 | 0 | 0 | 0 | 0 |  |  |  |  |  |  |  | 0.34 |  |  | 17 |  |  | 7 |  |
| 29 | STD | 0 | 0 | 0 | 0 | 0 | 0 | 0 | 0 | 0 | 0 | 0 | 0 | 0 | 0 |  |  |  |  |  | 0.36 | 0.34 | 0.24 | 13 | 12 | 7 | 6 |  |
| 30 | PROCT 10d | 1 | 0 | 0 | 0 | 0 | 1 | 0 | 0 | 1 | 0 | 0 | 0 |  |  | 1 |  |  |  |  | 0.06 |  |  | 3 |  |  | 9 |  |
| 31 | STD | 0 | 0 | 1 | 1 | 0 | 0 | 0 | 1 | 0 | 0 | 1 | 0 | 0 | 0 |  |  |  |  |  | 0.2 | 0.21 | 0.17 | 13 | 13 | 16 | 7 |  |
| 32 | PROCT 10d | 0 | 0 | 0 | 0 | 1 | 0 | 0 | 0 | 0 | 0 | 0 | 0 | 0 |  | 1 |  | 1 |  |  | 0.53 | 0.64 | 0.76 | 18 | 22 | 29 | 6 |  |
| 33 | PROCT 10d | 0 | 0 | 1 | 0 | 1 | 0 | 0 | 1 | 0 | 0 | 1 | 0 | 0 | 0 | 1 | 1 | 1 |  |  | -0.72 | -0.2 | -0.18 | -11 | -10 | -8 | 5 |  |
| 34 | STD | 0 | 0 | 0 | 0 | 1 | 0 | 0 | 0 | 0 | 0 | 0 | 0 | 0 | 0 |  |  |  |  |  | 0.56 | 0.25 | 0.65 | 20 | 9 | 23 | 6 |  |
| 35 | PROCT 3d | 0 | 0 | 0 | 0 | 1 | 0 | 0 | 0 | 0 | 0 | 0 | 0 | 0 | 0 |  | 1 |  |  |  | 0.24 | 0.04 | 0.87 | 8 | 1 | 30 | 6 |  |
| 36 | STD | 1 | 0 | 0 | 0 | 1 | 1 | 0 | 0 | 1 | 0 | 0 | 0 | 0 | 0 |  |  |  |  |  | 0.2 | -0.01 | -0.01 | 8 | 0 | -1 | 4 |  |
| 37 | STD | 1 | 0 | 0 | 0 |  | 1 | 0 | 0 | 1 | 0 | 0 | 0 |  |  |  |  |  |  |  |  |  |  |  |  |  |  |  |
| 38 | PROCT 10d | 0 | 0 | 0 | 0 | 1 | 0 | 0 | 0 | 0 | 0 | 0 | 0 | 0 | 0 |  |  |  |  |  | 0.75 | 0.37 | 0.74 | 25 | 12 | 25 | 2 |  |
| 39 | STD | 0 | 0 | 0 | 0 | 1 | 0 | 0 | 0 | 0 | 0 | 0 | 0 | 0 | 0 |  |  |  |  |  | 0.42 | 0.34 | 0.24 | 22 | 18 | 12 | 1 |  |
| 40 | PROCT 3d | 0 | 0 | 0 | 0 | 1 | 0 | 0 | 0 | 0 | 0 | 0 | 0 | 0 | 0 |  | 1 |  |  |  | 0.07 | -0.18 | -0.1 | 3 | -6 | -3 | 1 |  |
| 41 | STD | 0 | 0 | 0 | 0 | 1 | 0 | 0 | 0 | 0 | 0 | 0 | 0 |  |  |  |  |  |  |  |  |  |  |  |  |  | 6 |  |
| 42 | PROCT 3d | 0 | 1 | 0 | 1 | 1 | 0 | 0 | 0 | 0 | 0 | 0 | 0 | 0 |  |  |  |  |  |  | 0.23 |  |  | 9 |  |  | 5 |  |
| 43 | STD | 0 | 0 | 0 | 0 | 1 | 0 | 0 | 0 | 0 | 0 | 0 | 0 | 0 |  | 1 |  |  |  |  |  |  |  |  |  |  | 6 |  |
| 44 | PROCT 10d | 0 | 0 | 0 | 0 |  | 0 | 0 | 0 | 0 | 0 | 0 |  |  |  |  |  |  |  |  |  |  |  |  |  |  |  |  |
| 45 | PROCT 10d | 0 | 0 | 0 | 0 | 1 | 0 | 0 | 0 | 0 | 0 | 0 |  |  |  |  |  |  |  |  |  |  |  |  |  |  | 2 |  |
| 46 | PROCT 3d | 0 | 0 | 0 | 0 | 1 | 0 | 0 | 0 | 0 | 0 | 0 |  |  |  |  |  |  |  |  |  |  |  |  |  |  | 1 |  |
| 47 | STD | 0 | 0 | 0 | 0 | 1 | 0 | 0 | 0 | 0 | 0 | 0 |  |  |  |  |  |  |  |  |  |  |  |  |  |  | 4 |  |
| 48 | STD | 0 | 0 | 0 | 0 | 1 | 0 | 0 | 0 | 0 | 0 | 0 |  |  |  |  |  |  |  |  |  |  |  |  |  |  | 4 |  |
| 49 | STD | 0 | 0 | 0 | 0 | 1 | 0 | 0 | 0 | 0 | 0 | 0 | 0 | 0 | 0 |  |  |  |  |  | 0.22 | 0.1 | -0.06 | -33 | -38 | -45 | 5 | Cough, fever, dyspnea,  facial paralysis |
| 50 | STD | 0 | 1 | 0 | 1 | 0 | 0 | 0 | 0 | 0 | 0 | 0 | 0 | 0 | 0 |  | 1 |  |  |  | 0.49 | 0.42 | 0.49 | 17 | 14 | 17 | 5 |  |
| 51 | PROCT 3d | 0 | 0 | 0 | 0 | 1 | 0 | 0 | 0 | 0 | 0 | 0 | 0 | 0 | 0 |  |  |  |  |  | -0.22 | -0.27 | -0.14 | -9 | -11 | -6 | 6 |  |
| 52 | PROCT 10d | 0 | 1 | 0 | 1 | 1 | 0 | 0 | 0 | 0 | 0 | 0 | 0 | 0 | 0 |  | 1 | 1 |  |  | 53.53 | 0.01 | 0.09 | 3 | 0 | 5 | 1 |  |
| 53 | STD | 0 | 0 | 0 | 0 | 1 | 0 | 0 | 0 | 0 | 0 | 0 | 0 | 0 | 0 |  |  |  |  |  | -0.5 | -0.51 | -0.6 | -20 | -20 | -24 |  |  |
| 54 | PROCT 10d | 0 | 0 | 0 | 0 | 0 | 0 | 0 | 0 | 0 | 0 | 0 | 0 |  |  |  |  |  |  |  | 0.63 |  |  | 23 |  |  | 10 |  |
| 55 | PROCT 10d | 1 | 1 | 0 | 0 | 1 | 1 | 1 | 0 | 1 | 1 | 0 | 0 | 0 | 0 |  |  |  |  |  | -0.25 | -0.31 | -0.36 | -10 | -12 | -14 | 4 |  |
| 56 | STD | 0 | 0 | 0 | 0 | 0 | 0 | 0 | 0 | 0 | 0 | 0 |  |  |  |  |  |  |  |  |  |  |  |  |  |  | 7 |  |
| 57 | STD | 0 | 0 | 0 | 0 |  | 0 | 0 | 0 | 0 | 0 | 0 |  |  |  |  |  |  |  |  |  |  |  |  |  |  |  |  |
| 58 | STD | 0 | 0 | 0 | 0 |  | 0 | 0 | 0 | 0 | 0 | 0 |  |  |  |  |  |  |  |  |  |  |  |  |  |  |  |  |
| 59 | PROCT 3d | 0 | 0 | 0 | 0 | 1 | 0 | 0 | 0 | 0 | 0 | 0 | 0 | 0 | 0 |  |  |  |  |  | 0.56 | 0.32 | 0.27 | 23 | 14 | 10 | 8 |  |
| 60 | PROCT 3d | 0 | 0 | 0 | 0 |  | 0 | 0 | 0 | 0 | 0 | 0 |  |  |  |  |  |  |  |  |  |  |  |  |  |  |  |  |
| 61 | PROCT 3d | 1 | 0 | 0 | 1 | 0 | 0 | 0 | 0 | 0 | 0 | 0 | 0 | 0 | 0 |  |  | 1 |  |  | -0.06 | 0.64 | 0.02 | -2 | 26 | 0 | 7 |  |
| 62 | PROCT 10d | 0 | 0 | 0 | 0 | 0 | 0 | 0 | 0 | 0 | 0 | 0 | 0 | 0 | 0 | 1 | 1 | 1 |  |  | 0.21 | 0.18 | 0.04 | 5 | 4 | 1 | 7 |  |
| 63 | PROCT 10d | 0 | 0 | 0 | 0 | 1 | 0 | 0 | 0 | 0 | 0 | 0 | 0 | 0 | 0 |  |  |  |  |  | 0.28 | 0.41 | 0.58 | 12 | 17 | 24 | 7 |  |
| 64 | PROCT 10d | 0 | 0 | 1 | 1 | 1 | 0 | 0 | 0 | 0 | 1 | 0 |  | 0 | 0 |  |  |  |  |  |  | 0.13 | 0.2 |  | 7 | 7 | 5 |  |
| 65 | STD | 0 | 0 | 0 | 0 | 0 | 0 | 0 | 0 | 0 | 0 | 0 | 0 | 0 | 0 |  | 1 | 1 |  |  | 0.21 | 0.05 | 0.04 | 6 | 1 | 0 | 8 |  |
| 66 | PROCT 3d | 1 | 1 | 0 | 1 | 0 | 1 | 0 | 0 | 1 | 0 | 0 | 0 | 0 | 0 |  |  |  |  |  | 0.15 | 0.08 | 0.16 | 5 | 2 | 5 | 5 |  |
| 67 | PROCT 3d | 0 | 0 | 0 | 0 | 1 | 0 | 0 | 0 | 0 | 0 | 0 | 0 | 0 | 0 |  | 1 |  |  |  | -0.13 | -0.12 | -0.09 | -8 | -7 | -5 | 7 |  |
| 68 | PROCT 3d | 0 | 1 | 0 | 1 | 1 | 0 | 0 | 0 | 0 | 0 | 0 | 0 | 0 | 0 |  |  |  |  |  |  |  |  |  |  |  |  |  |
| 69 | PROCT 3d | 0 | 0 | 0 | 0 | 1 | 0 | 0 | 0 | 0 | 0 | 0 | 0 | 0 | 0 | 1 |  |  |  |  | -0.07 | 0.09 | 0.04 | -3 | 5 | 2 | 5 |  |
| 70 | STD | 0 | 0 | 0 | 0 | 1 | 0 | 0 | 0 | 0 | 0 | 0 |  |  |  |  |  |  |  |  |  |  |  |  |  |  | 10 |  |
| 71 | STD | 0 | 0 | 0 | 0 | 1 | 0 | 0 | 0 | 0 | 0 | 0 | 0 |  |  |  |  |  |  |  |  |  |  |  |  |  | 5 | High blood pressure levels |
| 72 | PROCT 3d | 0 | 1 | 1 | 1 | 1 | 0 | 0 | 0 | 0 | 0 | 0 | 0 | 0 | 0 |  |  |  |  |  | -0.02 | -0.04 | 0 | -1 | -1 | 1 | 2 |  |
| 73 | STD | 0 | 0 | 0 | 0 | 1 | 0 | 0 | 0 | 0 | 0 | 0 | 0 | 0 |  |  |  |  |  |  | 0.15 | 0.02 |  | 6 | 1 |  | 4 |  |
| 74 | PROCT 3d | 0 | 0 | 0 | 0 | 1 | 0 | 0 | 0 | 0 | 0 | 0 | 0 |  |  |  |  |  |  |  | 0.48 |  |  | 27 |  |  | 5 |  |
| 75 | PROCT 3d | 0 | 0 | 0 | 1 | 1 | 0 | 0 | 0 | 0 | 0 | 0 | 0 |  |  |  |  |  |  |  | -0.01 |  |  | 0 |  |  | 5 |  |
| 76 | STD | 0 | 1 | 0 | 1 | 1 | 0 | 0 | 0 | 0 | 0 | 0 | 0 | 0 | 0 | 1 | 1 | 1 |  |  | 0.15 | 0.11 | 0.11 | 6 | 4 | 4 | 2 |  |
| 77 | STD | 0 | 1 | 0 | 0 | 1 | 0 | 0 | 0 | 0 | 0 | 0 | 0 | 0 | 0 |  |  |  |  |  | -0.08 | 0.05 | 0.16 | -2 | 2 | 11 | 1 |  |
| 78 | STD | 0 | 0 | 0 | 0 | 1 | 0 | 0 | 0 | 0 | 0 | 0 | 0 | 0 | 0 |  |  |  |  |  | -0.07 | 0.1 | 0.1 | -2 | 4 | 4 | 7 |  |
| 79 | PROCT 3d | 0 | 0 | 0 | 0 | 1 | 0 | 0 | 0 | 0 | 0 | 0 | 0 | 0 | 0 |  |  |  |  |  | 0.95 | 1.08 | 0.34 | 32 | 37 | 12 | 5 |  |
| 80 | STD | 0 | 0 | 0 | 0 | 0 | 0 | 0 | 0 | 0 | 0 | 0 |  |  |  |  |  |  |  |  |  |  |  |  |  |  | 14 |  |
| 81 | PROCT 3d | 0 | 0 | 0 | 0 | 1 | 0 | 0 | 0 | 0 | 0 | 0 | 0 | 0 | 0 |  |  |  |  |  | 0.29 | 0.08 | -0.03 | 12 | 3 | -1 | 1 |  |
| 82 | PROCT 10d | 0 | 0 | 0 | 0 | 1 | 0 | 0 | 0 | 0 | 0 | 0 | 0 | 0 |  |  |  |  |  |  | 0.46 | 0.4 |  | 39 | 36 |  | 8 |  |
| 83 | STD | 0 | 1 | 1 | 1 | 1 | 0 | 0 | 0 | 0 | 0 | 0 | 0 | 0 | 0 |  |  |  |  |  | -0.03 |  |  | -4 |  |  |  |  |
| 84 | STD | 0 | 1 | 1 | 1 | 1 | 0 | 0 | 0 | 0 | 0 | 0 | 0 | 0 | 0 |  |  |  |  |  | 0.21 | 0.32 | 0.31 | 8 | 13 | 13 | 3 |  |
| 85 | PROCT 10d | 0 | 0 | 0 | 0 | 1 | 0 | 0 | 0 | 0 | 0 | 0 | 0 | 0 | 0 |  |  |  |  |  | -0.21 | -0.03 |  | -8 | -2 |  | 4 |  |
| 86 | STD | 0 | 0 | 0 | 0 | 0 | 0 | 0 | 0 | 0 | 0 | 0 |  |  |  |  |  |  |  |  |  |  |  |  |  |  | 1 | Pneumoniae  (SAE) |
| 87 | PROCT 3d | 0 | 0 | 0 | 1 | 1 | 0 | 0 | 0 | 0 | 0 | 0 | 0 |  |  |  |  |  |  |  |  |  |  |  |  |  | 9 |  |
| 88 | PROCT 10d | 0 | 0 | 0 | 0 | 0 | 0 | 0 | 0 | 0 | 0 | 0 |  |  |  |  |  |  |  |  |  |  |  |  |  |  | 3 |  |
| 89 | STD | 0 | 0 | 0 | 0 | 1 | 0 | 0 | 0 | 0 | 0 | 0 | 0 | 0 | 0 |  |  |  |  |  | -0.06 | -0.01 |  | -2 | 0 |  | 13 |  |
| 90 | STD | 0 | 0 | 0 | 0 | 1 | 0 | 0 | 0 | 0 | 0 | 0 | 0 | 0 | 0 |  |  | 1 |  |  | -0.16 | -0.19 | -0.23 | -7 | -9 | -17 | 2 |  |
| 91 | PROCT 10d | 0 | 0 | 0 | 0 | 1 | 0 | 0 | 0 | 0 | 0 | 0 |  |  |  |  |  |  |  |  |  |  |  |  |  |  | 4 |  |
| 92 | PROCT 10d | 0 | 1 | 0 | 1 | 1 | 0 | 0 | 0 | 0 | 0 | 0 | 0 | 0 | 0 |  |  |  |  |  | 0.02 | -0.24 | -0.28 | 0 | -8 | -11 |  |  |
| 93 | STD | 1 | 1 | 1 | 0 | 1 | 0 | 0 | 0 | 0 | 0 | 0 | 0 | 0 | 0 |  |  | 1 |  |  | 0.32 | 0.16 | 0.25 | 31 | 1 | 32 | 7 |  |
| 94 | PROCT 3d | 0 | 1 | 0 | 0 | 1 | 0 | 1 | 0 | 0 | 1 | 0 |  | 0 | 0 |  |  |  |  |  |  | -0.33 | -0.62 |  | -10 | -19 | 1 |  |
| 95 | STD | 0 | 1 | 1 | 0 | 1 | 0 | 0 | 0 | 0 | 0 | 0 | 0 | 0 | 0 | 1 |  |  |  |  | -0.14 | -0.13 | -0.03 | -5 | -4 | 0 | 3 |  |
| 96 | STD | 0 | 0 | 0 | 0 | 1 | 0 | 0 | 0 | 0 | 0 | 0 |  |  |  |  |  |  |  |  |  |  |  |  |  |  | 5 |  |
| 97 | PROCT 10d | 0 | 0 | 0 | 0 | 1 | 0 | 0 | 0 | 0 | 0 | 0 | 0 | 0 | 0 |  |  |  |  |  | -0.05 | -0.23 | 0.09 | 7 | -2 | 13 | 2 |  |
| 98 | PROCT 3d | 0 | 0 | 0 | 0 | 1 | 0 | 0 | 0 | 0 | 0 | 0 | 0 |  |  |  |  |  |  |  | 0.47 |  |  | 17 |  |  | 5 |  |
| 99 | STD | 0 | 0 | 0 | 1 | 0 | 0 | 0 | 0 | 0 | 0 | 0 | 0 | 0 | 0 |  |  | 1 |  |  | 0.06 | 0.11 | 0.09 | 1 | 4 | 3 | 7 |  |
| 100 | STD | 0 | 0 | 0 | 0 | 1 | 0 | 0 | 0 | 0 | 0 | 0 | 0 |  | 0 | 1 |  |  |  |  | 0.13 |  | 0.06 | 6 |  | 3 | 1 |  |
| 101 | PROCT 10d | 0 | 0 | 0 | 0 |  | 0 | 0 | 0 | 0 | 0 | 0 |  |  |  |  |  |  |  |  |  |  |  |  |  |  | 6 |  |
| 102 | PROCT 10d | 0 | 1 | 0 | 1 |  | 0 | 0 | 0 | 0 | 0 | 0 | 0 | 0 |  |  |  |  |  |  | 0.45 | 0 |  | 19 | 1 |  | 7 |  |
| 103 | STD | 0 | 0 | 0 | 0 | 1 | 0 | 0 | 0 | 0 | 0 | 0 | 0 | 0 | 0 |  |  |  |  |  | 0.09 | 0.16 | -0.05 | 0 | 6 | -2 | 7 |  |
| 104 | STD | 0 | 0 | 0 | 0 |  | 0 | 0 | 0 | 0 | 0 | 0 |  |  |  |  |  |  |  |  |  |  |  |  |  |  |  |  |
| 105 | STD | 0 | 0 | 0 | 0 | 1 | 0 | 0 | 0 | 0 | 0 | 0 | 0 | 0 |  |  |  |  |  |  | 0.28 | 0.07 |  | 11 | 4 |  | 7 |  |
| 106 | PROCT 3d | 0 | 0 | 0 | 0 | 1 | 0 | 0 | 0 | 0 | 0 | 0 | 0 | 0 |  |  |  |  |  |  | -0.21 | 0.04 |  | -5 | 5 |  | 7 |  |
| 107 | PROCT 10d | 0 | 0 | 0 | 0 | 1 | 0 | 0 | 0 | 0 | 0 | 0 | 0 |  |  |  |  |  |  |  | 0.12 |  |  | 3 |  |  | 3 |  |
| 108 | PROCT 3d | 0 | 0 | 0 | 0 |  | 0 | 0 | 0 | 0 | 0 | 0 |  |  |  |  |  |  |  |  |  |  |  |  |  |  |  |  |
| 109 | PROCT 3d | 0 | 0 | 0 | 0 | 1 | 0 | 0 | 0 | 0 | 0 | 0 | 0 | 0 |  | 1 | 1 |  |  |  |  |  |  |  |  |  | 6 |  |
| 110 | STD | 0 | 0 | 0 | 0 | 0 | 0 | 0 | 0 | 0 | 0 | 0 | 0 |  |  |  |  |  |  |  | 0.01 |  |  | 1 |  |  | 7 |  |
| 111 | PROCT 3d | 0 | 0 | 0 | 0 | 1 | 0 | 0 | 0 | 0 | 0 | 0 | 0 |  |  |  |  |  |  |  | 0.15 |  |  | 5 |  |  | 6 |  |
| 112 | STD | 0 | 0 | 0 | 0 | 0 | 0 | 0 | 0 | 0 | 0 | 0 |  |  |  |  |  |  |  |  |  |  |  |  |  |  | 7 |  |
| 113 | STD | 0 | 0 | 0 | 0 | 1 | 0 | 0 | 0 | 0 | 0 | 0 |  |  |  |  |  |  |  |  |  |  |  |  |  |  | 7 |  |
| 114 | PROCT 10d | 0 | 0 | 0 | 0 | 1 | 0 | 0 | 0 | 0 | 0 | 0 |  |  |  |  |  |  |  |  |  |  |  |  |  |  | 7 |  |
| 115 | PROCT 10d | 0 | 1 | 1 | 0 | 0 | 0 | 1 | 1 | 1 | 1 | 1 |  | 0 | 0 |  |  |  |  |  | 0.05 | -0.01 | 0 | 3 | 1 | 0 | 7 |  |
| 116 | PROCT 10d | 0 | 0 | 0 | 0 | 0 | 0 | 0 | 0 | 0 | 0 | 0 |  |  |  |  |  |  | 1 | |  |  |  |  |  |  | 7 | Death  (SAE) |
| 117 | STD | 0 | 0 | 0 | 0 | 0 | 0 | 0 | 0 | 0 | 0 | 0 | 0 | 0 | 0 |  |  |  |  |  |  |  |  |  |  |  | 7 |  |
| 118 | STD | 1 | 0 | 0 | 0 | 1 | 1 | 0 | 0 | 1 | 0 | 0 | 0 | 0 |  | 1 |  |  |  |  | 0.01 | 0.19 |  | 0 | 9 |  | 7 |  |
| 119 | PROCT 10d | 0 | 0 | 0 | 0 | 0 | 0 | 0 | 0 | 0 | 0 | 0 | 0 | 0 | 0 | 1 |  |  |  |  | 0.49 | 0.43 | 0.42 | 20 | 17 | 16 | 39 |  |
| 120 | STD | 0 | 0 | 0 | 0 | 0 | 0 | 0 | 0 | 0 | 0 | 0 | 0 | 0 | 0 | 1 |  |  |  |  | -0.16 | -0.2 | -0.08 | -5 | -6 | -2 | 5 |  |
| 121 | PROCT 10d | 0 | 0 | 0 | 0 | 0 | 0 | 0 | 0 | 0 | 0 | 0 | 0 | 0 | 0 | 1 |  |  |  |  |  | -0.01 | -0.01 |  | 0 | 0 | 7 |  |
| 122 | STD | 1 | 0 | 1 | 0 | 0 | 0 | 0 | 0 | 0 | 0 | 0 | 0 | 0 | 0 |  |  |  |  |  | 0.05 | 0.11 | 0.01 | 3 | 7 | -3 | 7 |  |
| 123 | STD | 0 | 0 | 1 | 1 | 1 | 0 | 0 | 1 | 0 | 0 | 1 | 0 | 0 | 0 |  | 1 | 1 |  |  | 0.16 | 0.09 | 0.08 | 3 | 5 | 6 | 5 |  |
| 124 | PROCT 3d | 0 | 0 | 0 | 0 | 1 | 0 | 0 | 0 | 0 | 0 | 0 |  |  |  |  |  |  | 1 | |  |  |  |  |  |  | 8 |  |
| 125 | STD | 0 | 0 | 0 | 0 |  | 0 | 0 | 0 | 0 | 0 | 0 |  | 0 |  |  |  |  |  |  |  | -0.19 |  |  | -8 |  | 8 |  |
| 126 | PROCT 3d | 1 | 1 | 0 | 1 |  | 0 | 0 | 0 | 0 | 0 | 0 | 0 | 0 |  |  |  |  |  |  | 0 | 0 |  | 0 | 0 |  | 12 |  |
| 127 | STD | 0 | 0 | 0 | 1 |  | 0 | 0 | 0 | 0 | 0 | 0 |  | 0 | 0 |  |  |  |  |  |  | -0.11 | 0.02 |  | -4 | -5 |  |  |
| 128 | STD | 0 | 0 | 0 | 0 |  | 0 | 0 | 0 | 0 | 0 | 0 |  |  |  |  |  |  |  |  |  |  |  |  |  |  | 11 |  |
| 129 | PROCT 10d | 0 | 0 | 0 | 0 | 1 | 0 | 0 | 0 | 0 | 0 | 0 | 0 | 0 | 0 | 1 | 1 |  |  |  | -0.04 | 0.11 | 0.15 | -1 | 7 | 9 | 8 |  |
| 130 | STD | 0 | 0 | 0 | 0 | 1 | 0 | 0 | 0 | 0 | 0 | 0 | 0 | 0 |  | 1 | 1 |  |  |  | 0.21 | 0.34 |  | 12 | 18 |  | 3 |  |
| 131 | PROCT 3d | 0 | 0 | 0 | 0 | 1 | 0 | 0 | 0 | 0 | 0 | 0 | 0 | 0 | 0 |  |  |  |  |  | 0.37 | 0.37 | 0.39 | 22 | 22 | 23 | 11 |  |
| 132 | STD | 0 | 0 | 0 | 0 | 1 | 0 | 0 | 0 | 0 | 0 | 0 | 0 | 0 | 0 |  |  |  |  |  | 0.28 | 0.36 | 0.28 | 10 | 13 | 10 | 11 |  |
| 133 | STD | 0 | 0 | 0 | 0 | 1 | 0 | 0 | 0 | 0 | 0 | 0 | 0 | 0 | 0 | 1 |  | 1 |  |  | 0.35 | 0.38 | 0.19 | 11 | 12 | 6 | 12 |  |
| 134 | STD | 0 | 0 | 0 | 0 | 1 | 0 | 0 | 0 | 0 | 0 | 0 |  |  |  |  |  |  |  |  |  |  |  |  |  |  | 6 |  |
| 135 | PROCT 3d | 0 | 0 | 0 | 0 | 1 | 0 | 0 | 0 | 0 | 0 | 0 | 0 | 0 | 0 |  |  |  |  |  | 0.33 | 0.33 | 0.41 | 26 | 26 | 32 | 4 |  |
| 136 | PROCT 10d | 0 | 0 | 0 | 0 |  | 0 | 0 | 0 | 0 | 0 | 0 |  |  |  |  |  |  |  |  |  |  |  |  |  |  |  |  |
| 137 | STD | 0 | 0 | 0 | 0 | 0 | 0 | 0 | 0 | 0 | 0 | 0 | 0 |  |  |  |  |  |  |  | 0.06 |  |  | 4 |  |  | 6 |  |
| 138 | STD | 0 | 0 | 0 | 1 | 1 | 0 | 0 | 0 | 0 | 0 | 0 | 0 | 0 | 0 |  |  |  |  |  | 0.05 | -0.1 | -0.02 | 2 | -5 | -2 | 5 |  |
| 139 | PROCT 10d | 0 | 0 | 0 | 0 | 1 | 0 | 0 | 0 | 0 | 0 | 0 | 0 | 0 | 0 |  |  |  |  |  | 0.12 | 0.07 | -0.09 | 6 | 4 | -3 | 13 |  |
| 140 | PROCT 10d | 0 | 0 | 0 | 0 | 0 | 0 | 0 | 0 | 0 | 0 | 0 | 0 | 0 | 0 | 1 |  |  |  |  | 0.13 | 0.27 | 0.23 | 4 | 7 | 7 | 7 |  |
| 141 | STD | 0 | 0 | 0 | 0 | 0 | 0 | 0 | 0 | 0 | 0 | 0 |  |  |  |  |  |  |  |  |  |  |  |  |  |  |  |  |
| 142 | STD | 0 | 0 | 0 | 0 | 0 | 0 | 0 | 0 | 0 | 0 | 0 | 0 | 0 | 0 |  |  |  |  |  | -0.08 | 0.33 | 0.07 | -3 | 14 | 3 | 7 |  |
| 143 | PROCT 3d | 0 | 0 | 0 | 0 | 0 | 0 | 0 | 0 | 0 | 0 | 0 | 0 | 0 | 0 |  |  |  |  |  | -0.01 | 0.01 | -0.02 | -1 | 0 | -1 | 12 |  |
| 144 | STD | 0 | 0 | 0 | 0 | 0 | 0 | 0 | 0 | 0 | 0 | 0 | 0 | 0 | 0 |  |  |  |  |  | 0.34 | 0.4 | 0.76 | 15 | 18 | 34 | 9 |  |
| 145 | PROCT 3d | 0 | 0 | 0 | 0 | 0 | 0 | 0 | 0 | 0 | 0 | 0 | 0 | 0 | 0 |  |  |  |  |  | 0.73 | 0.33 | 0.28 | 39 | 21 | 9 |  |  |
| 146 | PROCT 10d | 0 | 1 | 0 | 0 | 1 | 0 | 0 | 0 | 0 | 0 | 0 | 0 | 0 | 0 |  | 1 |  |  |  | -0.01 | -0.27 | -0.01 | 0 | -11 | 0 | 7 | Gastric pain, vomitus |
| 147 | PROCT 3d | 1 | 0 | 0 | 1 | 0 | 0 | 0 | 0 | 0 | 0 | 0 | 0 | 0 | 0 |  |  |  |  |  | -0.11 | 0.06 | -0.14 | -4 | 29 | -6 | 7 |  |
| 148 | STD | 0 | 0 | 1 | 1 | 0 | 0 | 0 | 1 | 0 | 0 | 1 | 0 | 0 | 0 | 1 |  | 1 |  |  | -0.14 | 0.1 | 0.26 | -4 | 4 | 10 | 7 |  |
| 149 | STD | 0 | 0 | 0 | 0 | 1 | 0 | 0 | 0 | 0 | 0 | 0 | 0 | 0 | 0 |  | 1 |  |  |  | 0.49 | 0.73 | 0.19 | 20 | 30 | 8 | 4 |  |
| 150 | STD | 0 | 1 | 0 | 0 | 1 | 0 | 0 | 0 | 0 | 0 | 0 | 0 | 0 | 0 |  | 1 |  |  |  | 0.06 | -0.07 | -0.11 | 2 | -3 | -5 | 5 |  |
| 151 | PROCT 3d | 0 | 0 | 0 | 0 | 1 | 0 | 0 | 0 | 0 | 0 | 0 | 0 | 0 | 0 |  |  |  |  |  | -0.24 | 0.1 | 0.06 | -11 | 6 | 5 | 8 |  |
| 152 | STD | 1 | 0 | 0 | 0 | 0 | 0 | 0 | 0 | 0 | 0 | 0 | 0 | 0 | 0 |  |  |  |  |  | -0.04 | 0.12 | 0 | -2 | 5 | 0 | 7 |  |
| 153 | PROCT 3d | 0 | 0 | 0 | 0 | 0 | 0 | 0 | 0 | 0 | 0 | 0 | 0 | 0 | 0 | 1 | 1 | 1 |  |  | 1.01 | -0.05 | 0.13 | 12 | -2 | 7 | 8 |  |
| 154 | PROCT 10d | 0 | 0 | 0 | 0 | 1 | 0 | 0 | 0 | 0 | 0 | 0 |  |  |  |  |  |  |  |  |  |  |  |  |  |  | 6 |  |
| 155 | PROCT 10d | 0 | 0 | 0 | 1 | 1 | 0 | 0 | 0 | 0 | 0 | 0 | 0 | 0 | 0 | 1 |  |  |  |  | -0.02 | 0.02 | 0.14 | -1 | 1 | 8 | 4 |  |
| 156 | STD | 1 | 0 | 0 | 1 | 1 | 0 | 0 | 0 | 0 | 0 | 0 | 0 | 0 | 0 | 1 |  |  |  |  | 0.01 | 0.28 | 0.39 | 0 | 11 | 16 | 5 |  |
| 157 | STD | 1 | 0 | 0 | 1 | 1 | 0 | 0 | 0 | 0 | 0 | 0 | 0 |  |  | 1 |  |  | 1 | | -0.08 |  |  | -3 |  |  | 4 | Death  (SAE) |
| 158 | STD | 0 | 0 | 0 | 0 | 0 | 0 | 0 | 0 | 0 | 0 | 0 | 0 | 0 | 0 |  |  |  |  |  | -0.12 | -0.12 | -0.14 | -5 | -5 | -6 | 8 |  |
| 159 | PROCT 10d | 0 | 0 | 0 | 0 | 1 | 0 | 0 | 0 | 0 | 0 | 0 | 0 |  |  |  |  |  |  |  | 0.39 |  |  | 18 |  |  | 8 |  |
| 160 | STD | 0 | 0 | 0 | 0 | 1 | 0 | 0 | 0 | 0 | 0 | 0 | 0 |  |  |  |  |  |  |  | 0.13 |  |  | 7 |  |  | 5 |  |
| 161 | PROCT 10d | 0 | 1 | 1 | 1 | 1 | 0 | 0 | 0 | 0 | 0 | 0 | 0 | 0 | 0 |  |  | 1 |  |  | 0.07 | -0.13 | -0.04 | 3 | -5 | -1 |  |  |
| 162 | STD | 0 | 0 | 0 | 0 | 1 | 0 | 0 | 0 | 0 | 0 | 0 |  |  |  |  |  |  |  |  |  |  |  |  |  |  | 5 |  |
| 163 | PROCT 10d | 1 | 0 | 0 | 0 | 1 | 1 | 0 | 0 | 1 | 0 | 0 | 1 |  |  |  |  |  | 1 | |  |  |  |  |  |  | 5 |  |
| 164 | PROCT 3d | 0 | 1 | 1 | 0 | 1 | 0 | 1 | 0 | 0 | 1 | 0 | 0 | 0 | 0 |  |  |  |  |  | -0.04 | -0.32 | -0.67 | -4 | -9 | -28 | 5 |  |
| 165 | PROCT 3d | 0 | 1 | 0 | 0 | 0 | 0 | 0 | 0 | 0 | 0 | 1 | 0 | 0 | 0 |  |  |  |  |  | -0.03 | -0.36 | -0.11 | -1 | -15 | -3 | 8 |  |
| 166 | STD | 0 | 1 | 1 | 0 | 1 | 0 | 0 | 0 | 0 | 0 | 0 | 0 | 0 | 0 |  |  |  |  |  | 0.11 | 0.12 | -0.2 | 4 | 4 | -4 | 6 |  |
| 167 | STD | 0 | 1 | 0 | 0 | 1 | 0 | 1 | 0 | 0 | 1 | 0 | 0 | 0 | 0 |  | 1 |  |  |  | 0.05 | 0.22 | -0.08 | 3 | 4 | -6 | 6 |  |
| 168 | STD | 0 | 0 | 0 | 0 | 0 | 0 | 0 | 0 | 0 | 0 | 0 | 0 |  |  |  |  |  |  |  | -0.25 |  |  | -7 |  |  | 8 |  |
| 169 | STD | 0 | 0 | 0 | 0 | 1 | 0 | 0 | 0 | 0 | 0 | 0 | 0 | 0 | 0 |  |  |  |  |  | 0.22 | -0.07 | -0.05 | 9 | -6 | -3 | 5 |  |
| 170 | STD | 0 | 0 | 0 | 0 | 0 | 0 | 0 | 0 | 0 | 0 | 0 | 0 |  |  |  |  |  |  |  |  |  |  |  |  |  | 8 |  |
| 171 | STD | 0 | 0 | 0 | 1 | 0 | 0 | 0 | 0 | 0 | 0 | 0 | 0 | 0 |  |  |  |  |  |  | 0.14 | 0.02 |  | 6 | 1 |  | 8 |  |
| 172 | PROCT 3d | 0 | 0 | 0 | 1 | 1 | 0 | 0 | 0 | 0 | 0 | 0 | 0 | 0 | 0 |  | 1 |  |  |  | 0.03 | -0.13 | -0.21 | 1 | -5 | -7 | 1 |  |
| 173 | PROCT 10d | 1 | 0 | 0 | 1 | 0 | 1 | 0 | 0 | 1 | 0 | 0 | 0 | 0 | 0 |  |  |  |  |  | -0.14 | -0.02 | -0.02 | -5 | 0 | 0 | 6 |  |
| 174 | PROCT 3d | 0 | 0 | 0 | 0 | 1 | 0 | 0 | 0 | 0 | 0 | 0 | 0 | 0 | 0 |  |  |  |  |  | 0.21 | 0.15 | 0.28 | 8 | 6 | 13 |  |  |
| 175 | PROCT 10d | 0 | 1 | 0 | 0 | 1 | 0 | 1 | 0 | 0 | 1 | 0 | 0 | 0 | 0 |  | 1 | 1 |  |  | 0.07 | 0.34 | 0.34 | -2 | 15 | 15 | 6 |  |
| 176 | STD | 0 | 0 | 0 | 0 | 1 | 0 | 0 | 0 | 0 | 1 | 0 | 0 | 0 | 0 |  |  |  |  |  | 0.27 | 0.09 | 0.27 | 15 | 5 | 15 | 3 |  |
| 177 | STD | 0 | 0 | 0 | 0 | 1 | 0 | 0 | 0 | 0 | 0 | 0 |  |  |  |  |  |  |  |  |  |  |  |  |  |  | 3 |  |
| 178 | STD | 0 | 0 | 0 | 0 | 1 | 0 | 0 | 0 | 0 | 0 | 0 | 0 | 0 | 0 |  |  |  |  |  | 0.06 | 0.06 | 0.01 | 18 | -2 | -3 | 5 |  |
