# Supplementary material for: Antibiotic Treatment of Severe Exacerbations of Chronic Obstructive Pulmonary Disease with Procalcitonin: A Randomized Noninferiority Trial
Source: PLoS One. 2015 Mar 11;10(3):e0118241. doi: 10.1371/journal.pone.0118241 (PMC4356612; doi:10.1371/journal.pone.0118241)
Supplement: S2 Table — (DOCX) [file pone.0118241.s007.docx]

**S2 Table**

| **Study center** | **Ethics Committee** | **Approval Number** | **Approval Date (d/m/y)** |
| --- | --- | --- | --- |
| University of Modena and Reggio Emilia  PI Leonardo M. Fabbri | Ethics Committee  Policlinico Modena | No. 118/06 | 05/09/2006 |
| University of Trieste  PI Marco Confalonieri | Ethics Committee  Ospedali Riuniti Trieste | No. 55/2006 | 20/12/2006 |
| University of Pavia  PI Ernesto Pozzi - Maurizio Luisetti | Ethics Committee  IRCCS Policlinico S. Matteo Pavia | No. 28602 | 13/11/2006 |
| University of Napoli  PI Serafino Marsico | Ethics Committee  Ospedale V. Monaldi Napoli | No. 32/06 | 15/11/2006 |
| University of Parma  PI Dario Olivieri | Ethics Committee  Azienda Ospedaliera-Universitaria Parma | No. 63/CE/2006 | 06/11/2006 |
| University of Genova  PI Giorgio W. Canonica | Ethics Committee  Ospedale S. Martino Genova | No. 161/2006 | 09/02/2007 |
| Fondazione Maugeri Cassano delle Murge (Bari)  PI Antonio Spanevello | Ethics Committee  IRCCS Fondazione Maugeri Pavia | No. CEC 451 | 08/01/2007 |
| Azienda USL Reggio Emilia  PI Luigi Zucchi | Ethics Committee  Azienda USL Reggio Emilia | No. 90/2006 | 25/09/2006 |
| University of Pisa  PI Pierluigi Paggiaro | Ethics Committee  Azienda Ospedaliero-Universitaria Pisa | No. 2218 | 14/12/2006 |
| University of Ferrara  PI Alberto Papi | Ethics Committee  Azienda Ospedaliero-Universitaria Ferrara | No. 061099 | 24/10/2006 |
| University of Foggia  PI Maria Pia Foschino Barbaro | Ethics Committee  Ospedali Riuniti Foggia | No. 44/CE/06 | 25/10/2006 |
| University of Catania  PI Nunzio Crimi | Ethics Committee  Azienda Ospedaliera P.O. Ascoli-Tomaselli Catania | No. 1415/CE | 29/12/2006 |
| IRCCS Istituto Clinico Humanitas  Rozzano (Milano)  PI Michele Ciccarelli | Ethics Committee  IRCCS Istituto Clinico Humanitas  Rozzano (Milano) | No. 1949 | 19/09/2006 |
| Ospedale San Bassiano  Bassano del Grappa (Vicenza)  PI Stefano Calabro | Ethics Committee  USL 3 Bassano del Grappa (Vicenza) | No. 22/06 | 20/12/2006 |
| Ospedali Riuniti  Bergamo  PI Andrea Rossi | Ethics Committee  Ospedali Riuniti Bergamo | No. 418 | 26/03/2007 |
| Ospedale Generale Regionale  Bolzano  PI Giulio Donazzan | Ethics Committee  Azienda Speciale USL Centro-Sud Bolzano | No. 38/2006 | 30/08/2006 |
| University of Padova  PI Renzo Zuin | Ethics Committee  Azienda Ospedaliero-Universitaria Padova | No. 1351P | 13/11/2006 |
| University of Firenze  PI Massimo Pistolesi | Ethics Committee  Ospedale Careggi Firenze | No. 543/2006 | 07/11/2006 |
