# Supplementary material for: Antibiotic Treatment of Severe Exacerbations of Chronic Obstructive Pulmonary Disease with Procalcitonin: A Randomized Noninferiority Trial
Source: PLoS One. 2015 Mar 11;10(3):e0118241. doi: 10.1371/journal.pone.0118241 (PMC4356612; doi:10.1371/journal.pone.0118241)
Supplement: S3 Table — (PDF) [file pone.0118241.s008.pdf]

S4 table

COMPARISON OF A SERUM PROCALCITONIN GUIDED TREATMENT PLAN WITH THE STANDARD GUIDELINE RECOMMENDED ANTIBIOTIC TREATMENT PLAN FOR PATIENTS HOSPITALIZED WITH A DIAGNOSIS OF EXACERBATION OF COPD

Center	No. of screened patients by center
1 Modena	19
2 Bassano	2
3 Genova	18
4 Milano	0
5 Trieste	9
6 Catania	16
7 Bolzano	4
8 Foggia	26
9 Napoli	18
10 Parma	6
11 Pisa	14
12 Ferrara	9
13 Firenze	0
14 Pavia	8
15 Bergamo	0
16 Cassano Murge	17
17 Reggio Emilia	17
18 Padova	0
